# Tailored [1,2,4]triazolo[1,5-*a*]pyrimidine hybrids as promising anti-inflammatory agents with COX/5-LOX/ROS/IL-6 multifunctional inhibitory activity: design, synthesis, SAR, and *in silico* study

**DOI:** 10.1039/d6ra04602a

**Published:** 2026-09-09

**Authors:** Esraa Elazony, Nermine A. Osman, Moataz A. Shaldam, Hanan A. Abdel-Fattah, Amany M. Ghanim

**Affiliations:** a Department of Pharmaceutical Organic Chemistry, Faculty of Pharmacy, Zagazig University Zagazig 44519 Egypt amanyghanim@yahoo.com AMMettwalli@pharmacy.zu.edu.eg; b Department of Pharmaceutical Chemistry, Faculty of Pharmacy, Kafrelsheikh University P. O. Box 33516 Kafrelsheikh Egypt; c Department of Pharmaceutical Chemistry, Faculty of Pharmacy, Alsalam University 31511 Tanta Egypt

## Abstract

To develop potent anti-inflammatory agents with safer profiles, a hybridization approach combining [1,2,4]triazolo[1,5-*a*]pyrimidine/chalcone (5a–g) and [1,2,4]triazolo[1,5-*a*]pyrimidine/pyrazole (6a–g) has been rationally designed to synthesize potential COX/5-LOX/ROS/IL-6 multifunctional inhibitors. All synthesized hybrids were evaluated *in vitro* using biological COX-1/COX-2 assays. Compounds 5a, 6c, and 6d displayed the highest COX-2 potency and selectivity (5a; IC_50_ = 1.361 µM, SI = 12.86, 6c; IC_50_ = 2.537 µM, SI = 18.05, and 6d; IC_50_ = 1.237 µM, SI = 8.917), outperforming the reference diclofenac sodium (IC_50_ = 4.812 µM, SI = 2.552). These promising derivatives were selected for further *in vitro* assays to evaluate their inhibitory effects on 5-LOX, ROS, and IL-6. The most potent COX-2 inhibitor 6d exhibited reasonable 5-LOX inhibitory activity (IC_50_ = 1.031 µM) in comparison to the selective 5-LOX inhibitor zileuton (IC_50_ = 0.386 µM). Moreover, 6d exhibited potent inhibitory activity against the proinflammatory cytokine IL-6 (90.9% inhibition) and ROS scavenging activity (IC_50_ = 34.45 µM), exceeding the two references, celecoxib and diclofenac sodium. Furthermore, compound 6c displayed substantial inhibition of TNF-α release (69.44%), approaching that of the reference drug diclofenac sodium (75.75%). Ultimately, docking simulations and drug-likeness studies provided insights into the potential binding scenarios within the active sites of COX-2 and 5-LOX, while also predicting the oral bioavailability and toxicity of the newly synthesized compounds. Based on these findings, the designed scaffolds can be considered as promising multitargeted anti-inflammatory candidates worthy of further exploration.

## Abstract 2

Triazolo[1,5-a]pyrimidine/chalcone (5a–g) and pyrazole (6a–g) hybrids were rationally designed and synthesized as safer, potent multifunctional COX/5-LOX/ROS/IL-6 inhibitors for development as anti-inflammatory agents.

## Graphical abstract

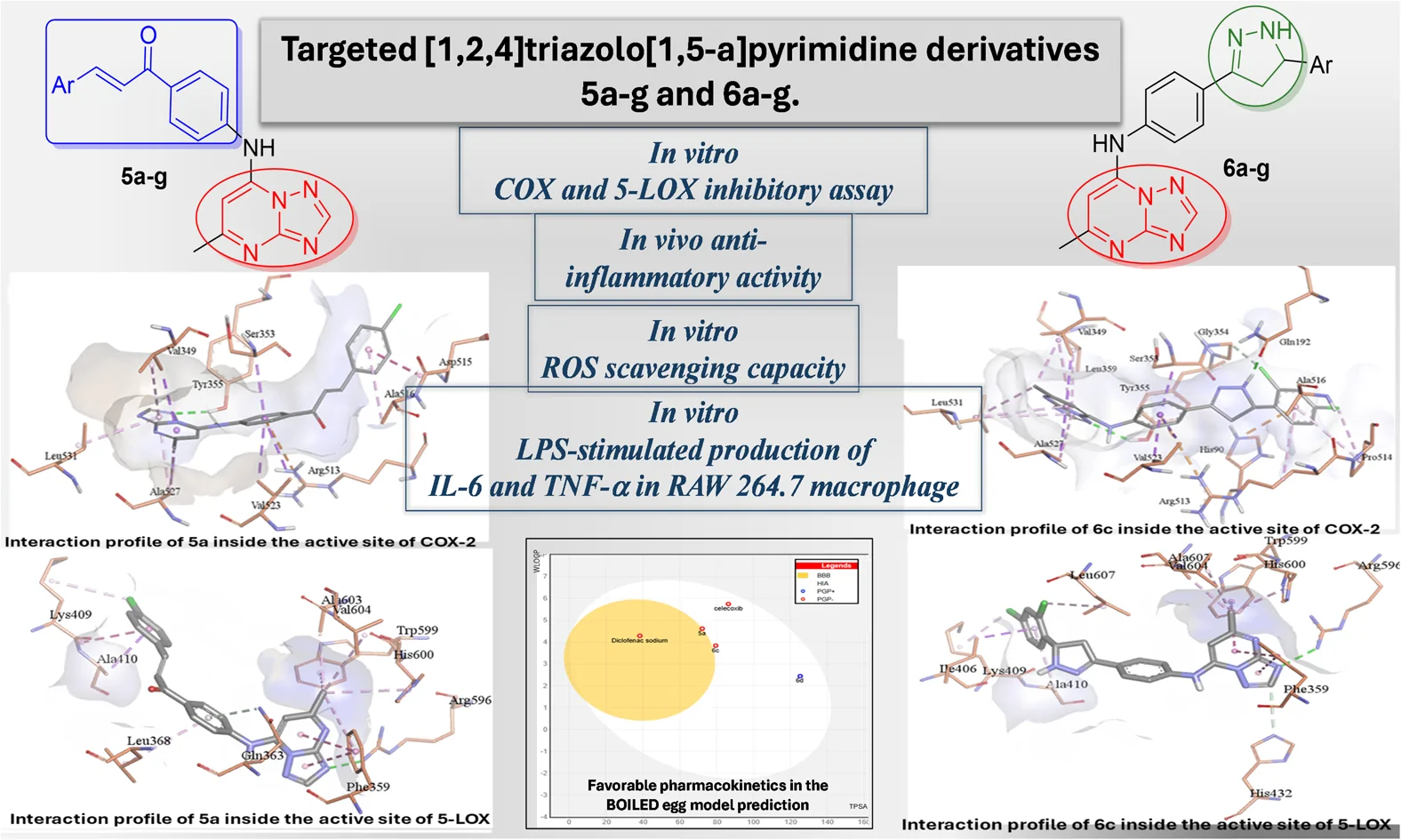

## 1 Introduction

Inflammation is an intrinsic defense mechanism of the body against any stimuli to maintain cellular homeostasis. However, any alteration to it may become detrimental.^1,2^ NSAIDs are commonly used to reduce inflammation by blocking cyclooxygenase (COX) enzymes. There are two main isoforms: COX-1, which is present in most cells, and COX-2, which is induced during inflammation and cancer development.^3^ COX enzymes activate the transformation of arachidonic acid into thromboxanes, prostaglandins (PGE_2_), and prostacyclins (PGI_2_). The beneficial effects of NSAIDs are credited to the lack of eicosanoids. However, their inhibition of COX-1 is responsible for gastrointestinal side effects.^1^ Traditional NSAIDs inhibit both COX-1 and COX-2, while selective COX-2 inhibitors (COXibs) target only COX-2 to reduce inflammation without gastrointestinal side effects. However, high selectivity for COX-2 can lead to cardiovascular issues, as COX-2 is needed for producing protective PGI_2_ in blood vessels. Inhibiting PGI_2_ with COXibs can result in serious cardiovascular events.^4,5^

Blocking the COX pathway shifts arachidonic acid metabolism towards the lipoxygenase pathway, leading to increased production of proinflammatory mediators like leukotrienes. This overproduction can contribute to inflammatory disorders such as asthma, rheumatoid arthritis, and various cancers.^6,7^ Designing drugs that block both cyclooxygenase and lipoxygenase pathways can achieve a more effective anti-inflammatory effect while ensuring safety.^8^ On the other hand, interleukin-6 (IL-6), a proinflammatory cytokine, plays a fundamental role in the pathogenesis of chronic inflammation^9^ and autoinflammatory diseases.^10^ The inflammatory process is also influenced by the excessive generation of reactive oxygen species (ROS). ROS cause the oxidation of biological macromolecules, damaging DNA, proteins, and lipids within cells. This oxidative stress can subsequently promote additional inflammatory reactions.^11^ Targeting ROS, along with the COX and LOX pathways, offers a more effective strategy for managing inflammation.

Research on the [1,2,4]-triazole core highlights its potential to downregulate inflammation by inhibiting COX and/or LOX pathways. The triazole I was approximately three times more potent than celecoxib, a typical COX-2 inhibitor (Fig. 1).^12^ The introduction of a furoxan moiety to the ester group resulted in the furoxan-[1,2,4]-triazole hybrid II, which demonstrated remarkable selective COX-2 inhibition (SI = 209) by enhancing interactions within the COX-2 active site compared to celecoxib. It also showed significant antioxidant activity by reducing NO in RAW264.7 cells (IC_50_ = 5.74 µM), surpassing celecoxib (Fig. 1).^13^

**Fig. 1:**
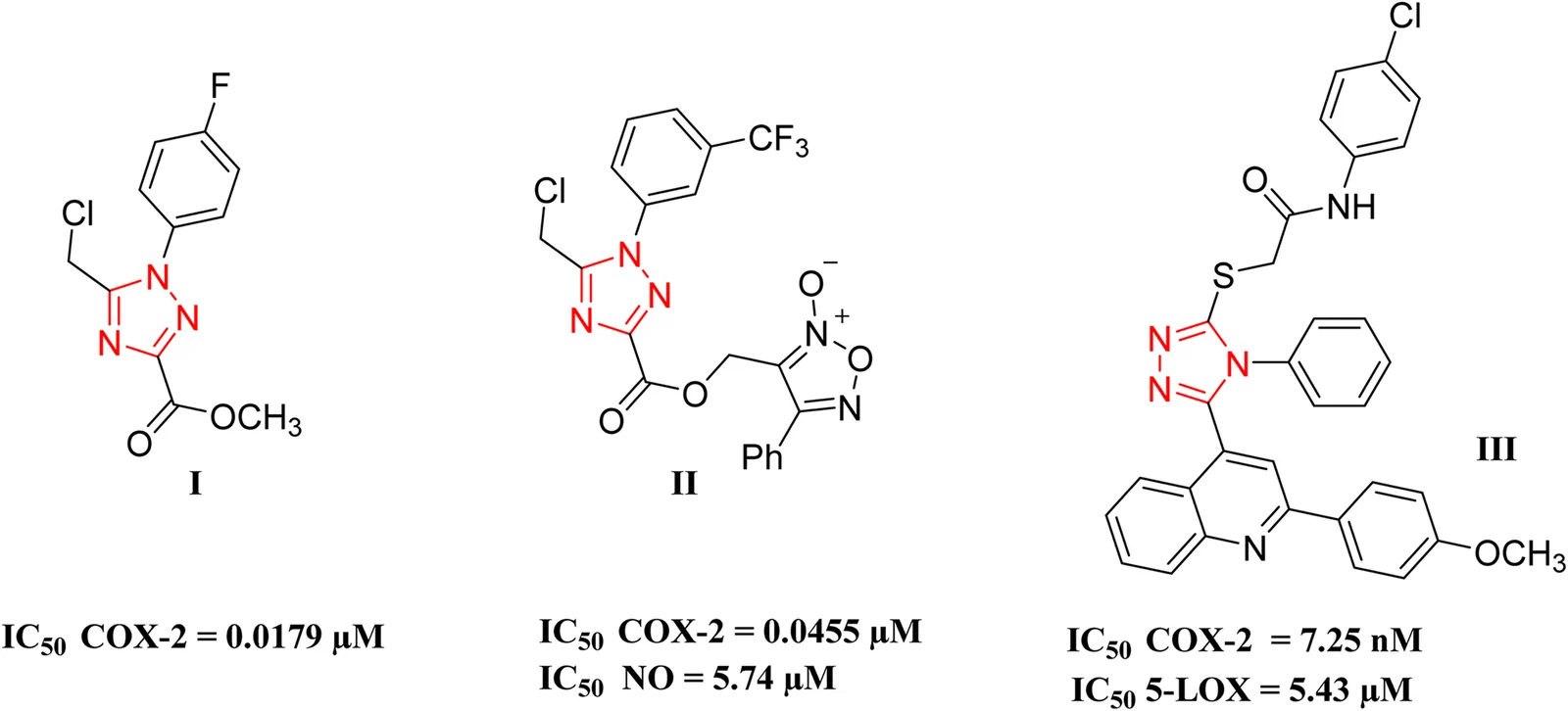
Reported [1,2,4]-triazole derivatives with remarkable activity against COX-2 and/or 5-LOX enzymes.

In another study, [1,2,4]-triazole-quinoline hybrid III exhibited a remarkable anti-inflammatory profile *via* dual COX-2/5-LOX inhibition, surpassing the reference drugs celecoxib and quercetin. Additionally, the preliminary test showed a notable reduction in serum pro-inflammatory cytokines (PGE_2_, TNF-α, and IL-6) (Fig. 1).^14^

The fused bicyclic [1,2,4]-triazole template is a promising scaffold for designing potent and safer anti-inflammatory/analgesic. The bioisosteric substitution of the carboxylic acid functionality in naproxen with a thiazolo[3,2-*b*]-[1,2,4]-triazole ring system resulted in the formation of hybrid IV (Fig. 2). This compound was proven to be more potent and selective compared to the standard drug indomethacin. Additionally, it demonstrated a significantly lower ulcerogenic risk than both indomethacin and naproxen.^15^ The triazolopyrimidine V (Fig. 2) showed effective COX-2 inhibition (IC_50_ = 10.50 µM) and a higher COX-2 selectivity (SI = 25) relative to the reference drug celecoxib. The anti-inflammatory activity was enhanced by 5-LOX inhibition, with an IC_50_ of 2.90 µM, which was lower than that of the reference drug quercetin.^5^

**Fig. 2:**
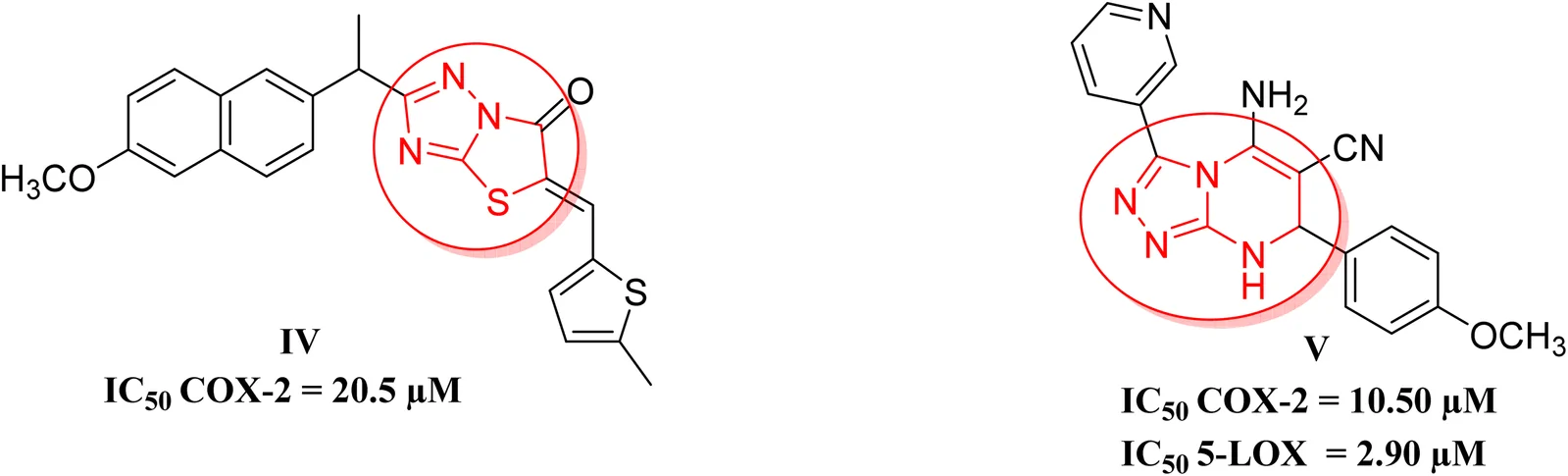
Reported fused bicyclic [1,2,4]-triazole-containing compounds with significant activity against COX-2 and/or 5-LOX enzymes.

Introducing chalcone or pyrazole linkers creates multitargeted drugs that inhibit pro-inflammatory enzymes COX and LOX, blocking the arachidonic acid pathway and modulating inflammatory cytokines. They also display antioxidant activity, enhancing their anti-inflammatory effects.^16–20^ The chalcone framework restricts access to the COX-1 pocket while fitting into the wider COX-2 channel. The lipophilic pyrazole scaffold is key for selective COX-2 inhibitors like celecoxib and valdecoxib, enhancing hydrophobic interactions in the COX-2 active site.^21^

For example, chalcone VI (Fig. 3) demonstrated promising anti-inflammatory activity by inhibiting both COX-2 and 5-LOX enzymes as well as reducing the production of the pro-inflammatory mediators PGE_2_ and TNF-α. Notably, the chalcone VI exhibited mild selectivity for COX-2 compared to celecoxib, resulting in a more favorable gastric profile. Moreover, it remarkably reduced the level of NO and iNOS.^22^ The halogenated chalcone VII (Fig. 3) effectively scavenged reactive oxygen species (ROS) (IC_50_ = 5.4 µg mL^−1^), outperforming ibuprofen. Notably, it inhibited the production of key proinflammatory mediators (IL-1β, IL-6, and TNF-α).^23^

**Fig. 3:**
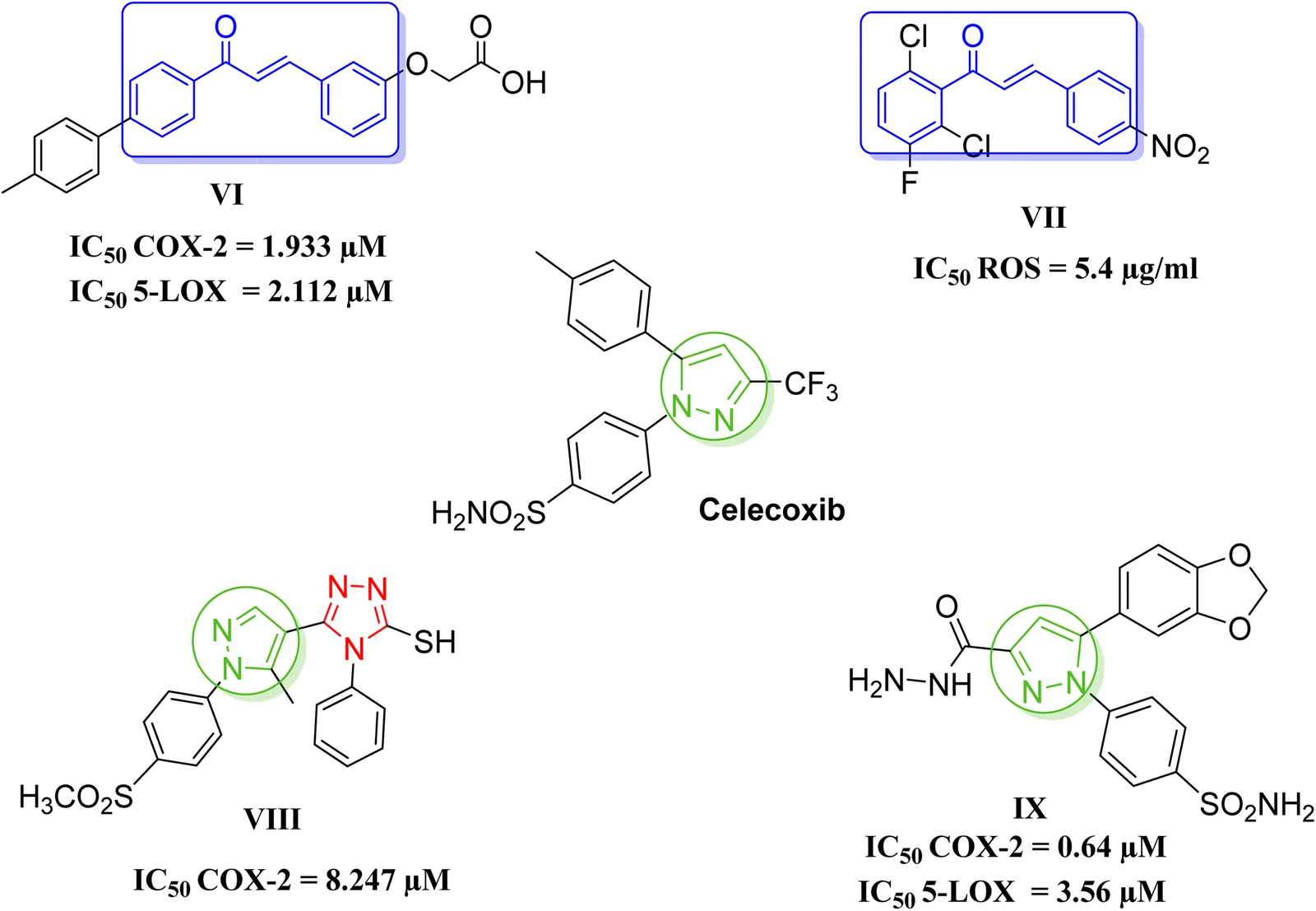
Reported chalcone and pyrazole derivatives as potential anti-inflammatory and antioxidant candidates.

Celecoxib is a selective COX-2 inhibitor used to manage arthritis pain. It has a tricyclic structure with a central pyrazole core, allowing it to fit into the unique COX-2 selectivity pocket, which is absent in COX-1.^24^ The triazole-pyrazole hybrid VIII (Fig. 3) demonstrated a high selectivity index (SI = 9.97) and had an IC_50_ lower than that of indomethacin, along with a higher *in vivo* anti-inflammatory profile with a better gastric profile than celecoxib.^25^ The pyrazole derivative IX (Fig. 3) exhibited significant *in vivo* analgesic and anti-inflammatory efficacy along with a high safety margin. Additionally, it exhibited dual COX-2/5-LOX inhibitory potential exceeding that of celecoxib and meclofenamate, respectively. The pyrazole moiety was an important pharmacophore, forming a hydrogen bond with Tyr341 and engaging in hydrophobic interactions within the COX-2 active site. Additionally, it showed greater free radical scavenging potential than ascorbic acid and significantly reduced TNF-α levels.^26^

Considering the existing literature, we designed our molecular hybridization architecture by combining two biologically active pharmacophores. Our approach involves incorporating a chalcone or a heterocyclic chalcone analogue, along with a pyrazole moiety, into the bicyclic [1,2,4]-triazolopyrimidine core (compounds 5a–g and 6a–g, respectively). This strategy aimed to enhance the overall anti-inflammatory activity and achieve multi-targeted action (Fig. 4). The designed hybrids were synthesized and assessed for their inhibitory potential on diverse inflammatory biotargets, COX1/2, 5-LOX, and IL-6. They were also evaluated for their activity to counteract ROS generation. Eventually, molecular modelling was utilized to emphasize the biological findings obtained.

**Fig. 4:**
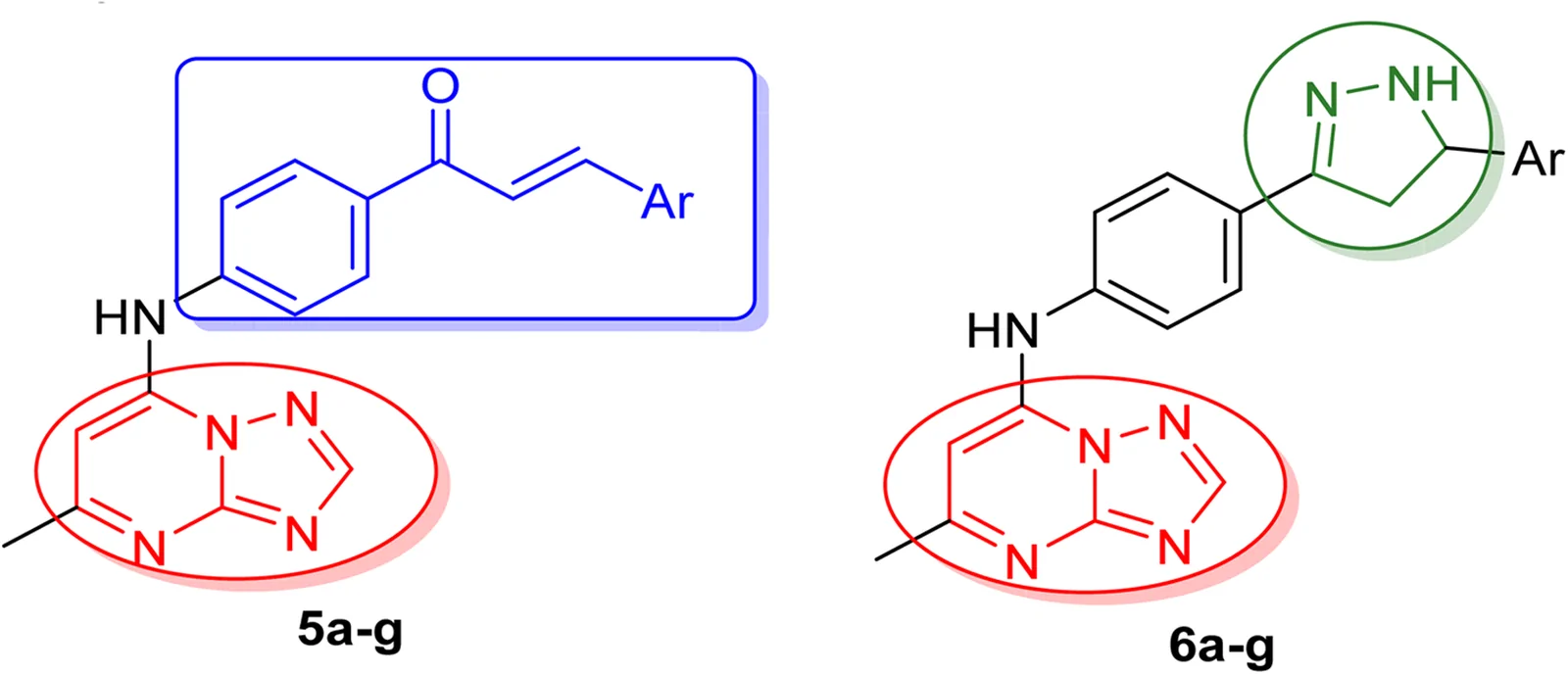
Graphical representation of designing our targeted [1,2,4]triazolo[1,5-*a*]pyrimidine derivatives 5a–g and 6a–g.

## 2. Experimental

### 2.1. Chemistry

All chemicals and solvents were sourced from well-known commercial suppliers. The Stuart SMP10 digital apparatus measured the melting points (°C). NMR spectra were obtained using a Bruker Avance III operating at 400 MHz for ^1^H NMR and at 101 MHz for ^13^C NMR and ^13^C APT NMR (Zagazig University). The IR spectra were detected using a Bruker ALPHA spectrometer equipped with a potassium bromide (KBr) beamsplitter (Zagazig University). The GC/MS Mat 112 S mass spectrometer was used to obtain the mass spectra *via* the EI^+^ ionization technique (Al-Azhar University). The Elementar Vario MICRO cube was used to determine the elemental analyses (Al-Azhar University). TLC (silica gel 60 GF245) was used to track the progress of each reaction, and a 254 nm UV lamp was used for visualization. Compounds 2 and 3 were prepared according to the reported procedure.^27^

#### 2.1.1. 1-(4-((5-Methyl-[1,2,4]triazolo[1,5-*a*]pyrimidin-7-yl)amino)phenyl)ethan-1-one(4)

A mixture of compound 3 (0.26 g, 1 mmol) and *p*-aminoacetophenone (0.13 g, 1 mmol) was dissolved in absolute ethanol (10 mL), followed by the addition of triethylamine (0.30 g, 3 mmol). The mixture was continuously stirred for five hours at room temperature. The resultant mixture was concentrated under reduced pressure to a residue, which was then subjected to column chromatographic purification. The column was packed with hexane and eluted with a hexane-ethyl acetate mixture (9 : 1). White solid (55% yield). M.p. 170–172 °C. IR: *ν*_max_/cm^−1^; 3198, 3077, 2965, 2835, 1670, 1593, 1355, 1252. ^1^H NMR (400 MHz, DMSO-*d*_6_) *δ* ppm: 10.48 (s, 1H, NH, exchangeable with D_2_O), 8.54 (s, 1H, C_2_–H of triazolopyrimidine), 8.04 (d, *J* = 8.6 Hz, 2H, Ar–H), 7.62 (d, *J* = 8.6 Hz, 2H, Ar–H), 6.69 (s, 1H, C_6_–H of triazolopyrimidine), 2.59 (s, 3H, **CH**_**3**_CO), 2.47 (s, 3H, CH_3_). ^13^C APT NMR (101 MHz, DMSO-*d*_6_) *δ* ppm: 196.7 (CO), 164.7 (C triazolopyrimidine), 155.5 (C triazolopyrimidine), 154.5 (CH triazolopyrimidine), 144.7 (C triazolopyrimidine), 141.7 (Ar–C), 133.4 (Ar–C), 129.7 (Ar–CH), 122.5 (Ar–CH), 90.5 (CH triazolopyrimidine), 26.6 (**CH**_**3**_CO), 24.8 (CH_3_). Analysis calcd for C_14_H_13_N_5_O: C, 62.85; H, 4.86; N, 26.19. Found: C, 62.71; H, 5.02; N, 26.37. MS (EI^+^), *m*/*z*:267.71 [M^+^].

#### 2.1.2. General synthetic procedure for (*E*)-3-(substituted phenyl)-1-(4-((5-methyl-[1,2,4]triazolo[1,5-*a*]pyrimidin-7-yl)amino)phenyl)prop-2-en-1-one (5a–g)

A mixture of compound 4 (1 g, 4 mmol) in an ethanol solution (70% w/v, 10 mL) and KOH (12 mmol) was stirred for 30 min. Then, a proper aldehyde (20 mmol) was added and stirred for 24 h at room temperature. The formed precipitate was filtered off and washed with petroleum ether to remove excess aldehyde, followed by washing with water. The separated products were air-dried, then recrystallized from ethanol to give compounds 5a–g.

##### 2.1.2.1 (*E*)-3-(4-chlorophenyl)-1-(4-((5-methyl-[1,2,4]triazolo[1,5-*a*]pyrimidin-7-yl)amino)phenyl)prop-2-en-1-one(5a)

Orange crystals (65% yield). M.p. 276–278 °C. IR: *ν*_max_/cm^−1^; 3375, 3059, 2923, 1661, 1595, 1558, 1329, 1254. ^1^H NMR (400 MHz, DMSO-*d*_6_) *δ* ppm: 10.58 (s, 1H, NH, exchangeable with D_2_O), 8.32 (s, 1H, C_2_–H of triazolopyrimidine), 8.20 (d, *J* = 6.9 Hz, 2H, Ar–H), 8.01 (d, *J* = 15.7 Hz, 1H, vinylic H), 7.94 (d, *J* = 6.9 Hz, 2H, Ar–H), 7.72 (d, *J* = 15.7 Hz, 1H, vinylic H), 7.53 (d, *J* = 7.3 Hz, 2H, Ar–H), 7.45 (d, *J* = 7.3 Hz, 2H, Ar–H), 6.42 (s, 1H, C_6_–H of triazolopyrimidine), 2.36 (s, 3H, CH_3_). ^13^C APT NMR (101 MHz, DMSO-*d*_6_) *δ* ppm: 187.3 (CO), 163.2 (C triazolopyrimidine), 156.2 (C triazolopyrimidine), 153.7 (CH triazolopyrimidine), 146.1 (C triazolopyrimidine), 146.0 (Ar–C), 141.8 (vinylic CH), 134.9 (Ar–C), 133.8 (Ar–C), 132.3 (Ar–C), 130.5 (Ar–CH), 130.3 (Ar–CH), 129.0 (Ar–CH), 122.9 (vinylic CH), 122.3 (Ar–CH), 90.1 (CH triazolopyrimidine), 24.6 (CH_3_). Analysis calcd for C_21_H_16_ClN_5_O: C, 64.64; H, 4.10; N, 17.96. Found: C, 64.50; H, 4.23; N, 18.05. MS (EI^+^), *m*/*z*: 389.52 [M^+^].

##### 2.1.2.2 (*E*)-3-(2-chlorophenyl)-1-(4-((5-methyl-[1,2,4]triazolo[1,5-*a*]pyrimidin-7-yl)amino)phenyl)prop-2-en-1-one (5b)

Orange crystals (89% yield). M.p. 271–273 °C. IR: *ν*_max_/cm^−1^; 3455, 3170, 2918, 1650, 1509, 1444, 1334, 1272. ^1^H NMR (400 MHz, DMSO-*d*_6_) *δ* ppm: 10.58 (s, 1H, NH, exchangeable with D_2_O), 8.22 (s, 1H, C_2_–H of triazolopyrimidine), 8.09 (d, *J* = 7.5 Hz, 2H, Ar–H), 8.00–7.96 (m, 3H, 2× Ar–H and vinylic H), 7.61–7.44 (m, 3H, 2× Ar–H and vinylic H), 7.16–7.06 (m, 2H, Ar–H), 5.92 (s, 1H, C_6_–H of triazolopyrimidine), 2.16 (s, 3H, CH_3_). ^13^C APT NMR (101 MHz, DMSO-*d*_6_) *δ* ppm: 186.4 (CO), 160.1 (C triazolopyrimidine), 157.4 (C triazolopyrimidine), 152.3 (CH triazolopyrimidine), 148.7 (C triazolopyrimidine), 140.3 (Ar–C), 136.7 (vinylic CH), 134.1 (Ar–C), 132.7 (Ar–C), 131.6 (Ar–CH), 130.5 (Ar–CH), 130.0 (Ar–CH), 129.0 (Ar–C), 128.5 (Ar-CH), 127.7 (Ar–CH), 125.2 (vinylic CH), 121.8 (Ar–CH), 88.8 (CH triazolopyrimidine), 24.4 (CH_3_). Analysis calcd for C_21_H_16_ClN_5_O : C, 64.64; H, 4.10; N, 17.96. Found: C, 64.89; H, 4.21; N, 18.15. MS (EI^+^), *m*/*z*: 389.71 [M^+^].

##### 2.1.2.3 (*E*)-3-(2,4-dichlorophenyl)-1-(4-((5-methyl-[1,2,4]triazolo[1,5-*a*]pyrimidin-7-yl)amino)phenyl)prop-2-en-1-one(5c)

Red crystals (95% yield). M.p. 258–260 °C. IR: *ν*_max_/cm^−1^; 3336, 3236, 3091, 2980, 1657, 1585, 1557, 1334, 1311. ^1^H NMR (400 MHz, DMSO-*d*_6_) *δ* ppm: 11.56 (s, 1H, NH, exchangeable with D_2_O), 8.36 (s, 1H, C_2_–H of triazolopyrimidine), 8.25 (d, *J* = 8.7 Hz, 1H, Ar–H), 8.20 (d, *J* = 8.4 Hz, 2H, Ar–H), 8.05 (d, *J* = 15.5 Hz, 1H, vinylic H), 7.94 (d, *J* = 15.5 Hz, 1H, vinylic H), 7.71 (d, *J* = 2.0 Hz, 1H, Ar–H), 7.53 (dd, *J* = 8.7, 2.0 Hz, 1H, Ar–H), 7.49 (d, *J* = 8.4 Hz, 2H, Ar–H), 6.49 (s, 1H, C_6_–H of triazolopyrimidine), 2.38 (s, 3H, CH_3_). ^13^C APT NMR (101 MHz, DMSO-*d*_6_) *δ* ppm: 187.0 (CO), 163.2 (C triazolopyrimidine), 156.1 (C triazolopyrimidine), 153.8 (CH triazolopyrimidine), 146.9 (C triazolopyrimidine), 146.0 (Ar–C), 136.5 (vinylic CH), 135.5 (Ar–C), 135.1 (Ar–C), 132.0 (Ar–C), 131.5 (Ar–C), 130.4 (Ar–CH), 129.8 (Ar–CH), 129.5 (Ar–CH), 127.9 (Ar–CH), 125.3 (vinylic CH), 122.3 (Ar–CH), 90.2 (CH triazolopyrimidine), 24.7 (CH_3_). Analysis calcd for C_21_H_15_Cl_2_N_5_O: C, 59.39; H, 3.53; N, 16.50. Found: C, 59.51; H, 3.70; N, 16.74. MS (EI^+^), *m*/*z*: 424.27 [M^+^].

##### 2.1.2.4 (*E*)-1-(4-((5-methyl-[1,2,4]triazolo[1,5-*a*]pyrimidin-7yl)amino)phenyl)-3-(4-nitrophenyl)prop-2-en-1-one(5d)

Brown crystals (85% yield). M.p. 268–270 °C. IR: *ν*_max_/cm^−1^; 3559, 3098, 2923, 1759, 1667, 1591, 1566, 1338, 1315. ^1^H NMR (400 MHz, DMSO-*d*_6_) *δ* ppm: 10.54 (s, 1H, NH, exchangeable with D_2_O), 8.40–8.25 (m, 4H, 1H C_2_–H of triazolopyrimidine, 2H Ar–H and 1H vinylic H), 8.24–8.14 (m, 4H, Ar–H), 7.80 (d, *J* = 15.9 Hz, 1H, vinylic H), 7.49–7.39 (m, 2H, Ar–H), 6.41 (s, 1H, C_6_–H of triazolopyrimidine), 2.34 (s, 3H, CH_3_). ^13^C APT NMR (100 MHz, DMSO-*d*_6_) *δ* ppm: 186.5 (CO), 160.6 (C triazolopyrimidine), 157.1 (C triazolopyrimidine), 152.5 (CH triazolopyrimidine), 148.5 (C triazolopyrimidine), 147.84 (Ar–C), 147.79 (Ar–C), 141.6 (Ar–C), 139.5 (vinylic CH), 130.5 (Ar–CH), 129.7 (Ar–CH), 129.4 (Ar–C), 126.5 (Ar–CH), 123.9 (vinylic CH), 122.0 (Ar–CH), 89.1 (CH triazolopyrimidine), 24.4 (CH_3_). Analysis calcd for C_21_H_16_N_6_O_3_ : C, 62.94; H, 4.00; N, 20.98. Found: C, 62.70; H, 4.23; N, 21.24. MS (EI^+^), *m*/*z*: 400.21 [M^+^].

##### 2.1.2.5 (*E*)-3-(4-bromophenyl)-1-(4-((5-methyl-[1,2,4]triazolo[1,5-*a*]pyrimidin-7-yl)amino)phenyl)prop-2-en-1-one(5e)

Yellow crystals (79% yield). M.p. 273–275 °C. IR: *ν*_max_/cm^−1^; 3312, 2989, 1660, 1621, 1585, 1330, 1317, 861. ^1^H NMR (400 MHz, DMSO-*d*_6_) *δ* ppm: 10.55 (s, 1H, NH, exchangeable with D_2_O), 8.40 (s, 1H, C_2_–H of triazolopyrimidine), 8.22 (d, *J* = 7.3 Hz, 2H, Ar–H), 8.03 (d, *J* = 15.6 Hz, 1H, vinylic H), 7.87 (d, *J* = 8.0 Hz, 2H, Ar–H), 7.73 (s, 1H, vinylic H), 7.68–7.66 (m, 2H, 2× Ar–H), 7.53 (d, *J* = 7.3 Hz, 2H, Ar–H), 6.54 (s, 1H, C_6_–H of triazolopyrimidine), 2.40 (s, 3H, CH_3_). ^13^C APT NMR (101 MHz, DMSO-*d*_6_) *δ* ppm: 187.4 (CO), 163.5 (C triazolopyrimidine), 156.0 (C triazolopyrimidine), 153.9 (CH triazolopyrimidine), 145.7 (C triazolopyrimidine), 145.6 (Ar–C), 142.0 (vinylic CH), 134.1 (Ar–C), 132.7 (Ar–C), 131.9 (Ar–CH), 130.7 (Ar–CH), 130.2 (Ar–CH), 123.8 (Ar–C), 122.9 (vinylic CH), 122.4 (Ar–CH), 90.3 (CH triazolopyrimidine), 24.7 (CH_3_). Analysis calcd for C_21_H_16_BrN_5_O: C, 58.02; H, 3.68; N, 16.12. Found: C, 58.29; H, 3.79; N, 16.30. MS (EI^+^), *m*/*z*: 433.88 [M^+^].

##### 2.1.2.6 (*E*)-1-(4-((5-methyl-[1,2,4]triazolo[1,5-*a*]pyrimidin-7-yl)amino)phenyl)-3-(thiophen-2-yl)prop-2-en-1-one(5f)

Yellow crystals (96% yield). M.p. 232–234 °C. IR: *ν*_max_/cm^−1^; 3521, 3302, 3063, 1653, 1589, 1504, 1345, 1325, 871. ^1^H NMR (400 MHz, DMSO-*d*_6_) *δ* ppm: 10.52 (s, 1H, NH, exchangeable with D_2_O), 8.54 (s, 1H, C_2_–H of triazolopyrimidine), 8.20 (d, *J* = 8.7 Hz, 2H, Ar–H), 7.94 (d, *J* = 15.3 Hz, 1H, vinylic H), 7.80 (d, *J* = 5.0 Hz, 1H, thienyl-H), 7.71 (d, *J* = 3.5 Hz, 1H, thienyl-H), 7.66 (d, *J* = 8.7 Hz, 2H, Ar–H), 7.61 (d, *J* = 15.3 Hz, 1H, vinylic H), 7.21 (dd, *J* = 5.0, 3.5 Hz, 1H, thienyl-H), 6.74 (s, 1H, C_6_–H of triazolopyrimidine), 2.48 (s, 3H, CH_3_). ^13^C APT NMR (101 MHz, DMSO-*d*_6_) *δ* ppm: 187.2 (CO), 164.6 (C triazolopyrimidine), 155.6 (C triazolopyrimidine), 154.4 (CH triazolopyrimidine), 144.7 (C triazolopyrimidine), 142.1 (Ar–C), 139.8 (thienyl-C), 136.6 (vinylic CH), 133.9 (Ar–C), 132.9 (Ar–CH), 130.5 (thienyl-CH), 130.0 (thienyl-CH), 128.8 (thienyl-CH), 122.6 (vinylic CH), 120.2 (Ar–CH), 90.7 (CH triazolopyrimidine), 24.8 (CH_3_). Analysis calcd for C_19_H_15_N_5_OS: C, 63.08; H, 4.15; N, 19.37. Found: C, 62.91; H, 4.28; N, 19.51. MS (EI^+^), *m*/*z*: 361.34 [M^+^].

##### 2.1.2.7 (*E*)-1-(4-((5-methyl-[1,2,4]triazolo[1,5-*a*]pyrimidin-7-yl)amino)phenyl)-3-(naphthalen-1-yl)prop-2-en-1-one(5g)

Yellow crystals (80% yield). M.p. 265–267 °C. IR: *ν*_max_/cm^−1^; 3214, 1644, 1621, 1585, 1405, 1350, 1318, 872. ^1^H NMR (400 MHz, DMSO-*d*_6_) *δ* ppm: 10.54 (s, 1H, NH, exchangeable with D_2_O), 8.59 (d, *J* = 15.2 Hz, 1H, vinylic H), 8.54 (s, 1H, C_2_–H of triazolopyrimidine), 8.32–8.27 (m, 4H, 3H naphthyl-H and 1H vinylic H), 8.08 (d, *J* = 7.5 Hz, 2H, Ar–H), 8.05–8.02 (m, 1H, naphthyl-H), 7.69 (d, *J* = 7.5 Hz, 2H, Ar–H), 7.66–7.60 (m, 3H, naphthyl-H), 6.74 (s, 1H, C_6_–H of triazolopyrimidine), 2.48 (s, 3H, CH_3_). ^13^C APT NMR (101 MHz, DMSO-*d*_6_) *δ* ppm: 187.6 (CO), 164.3 (C triazolopyrimidine), 155.7 (C triazolopyrimidine), 154.3 (CH triazolopyrimidine), 145.0 (C triazolopyrimidine), 144.9 (Ar–C), 139.6 (vinylic CH), 133.6 (Ar–C), 133.4 (Ar–C), 131.4 (Ar–C), 131.2 (Ar–C), 130.9 (Ar–CH), 130.5 (Ar–CH), 130.3 (Ar–CH), 128.8 (Ar–CH), 127.3 (Ar–CH), 126.4 (Ar–CH), 125.7 (Ar–CH), 124.5 (Ar–CH), 123.0 (vinylic CH), 122.6 (Ar–CH), 90.6 (CH triazolopyrimidine), 24.8 (CH_3_). Analysis calcd for C_25_H_19_N_5_O: C, 73.99; H, 4.69; N, 17.26. Found: C, 74.15; H, 4.82; N, 17.48. MS (EI^+^), *m*/*z*: 405.46 [M^+^].

#### 2.1.3 General synthetic procedure for *N*-(4-(5-(substituted phenyl)-4,5-dihydro-1*H*-pyrazol-3-yl)phenyl)-5-methyl-[1,2,4]triazolo[1,5-*a*]pyrimidin-7-amine(6a–g)

A mixture of the corresponding chalcones 5a–g (0.01 mol) was stirred in neat hydrazine hydrate (2 mL, 0.04 mol) at room temperature for 24 h. The reaction was quenched by pouring it into ice. The precipitate that formed was filtered out, thoroughly washed with water, and then crystallized from a methylene chloride/hexane mixture (10 : 1) to give compounds 6a–g. Final yields (50–67%) reflect the use of recrystallization from the methylene chloride/hexane mixture (10 : 1); while this method ensures excellent analytical purity, minor losses occurred during the washing and filtration stages.

##### 2.1.3.1 *N*-(4-(5-(4-chlorophenyl)-4,5-dihydro-1*H*-pyrazol-3-yl)phenyl)-5-methyl-[1,2,4]triazolo[1,5-*a*]pyrimidin-7-amine(6a)

Yellow crystals (50% yield). M.p. 180–182 °C. IR: *ν*_max_/cm^−1^; 3262, 3213, 2925, 1655, 1557, 1368, 1317, 1011, 770. ^1^H NMR (400 MHz, DMSO-*d*_6_) *δ* ppm: 10.11 (s, 1H, NH, exchangeable with D_2_O), 8.50 (s, 1H, C_2_–H of triazolopyrimidine), 7.70 (d, *J* = 6.1 Hz, 2H, Ar–H), 7.67 (s, 1H, NH, exchangeable with D_2_O), 7.47 (d, *J* = 6.1 Hz, 2H, Ar–H), 7.46–7.41 (m, 4H, Ar–H), 6.46 (s, 1H, C_6_–H of triazolopyrimidine), 4.93–4.77 (m, 1H, C_5_–H of pyrazole), 3.57–3.40 (m, 1H, C_4_-H_A_H_B_ of pyrazole), 2.85 (dd, *J* = 15.7, 11.6 Hz, 1H, C_4_-H_A_H_B_ of pyrazole), 2.42 (s, 3H, CH_3_). ^13^C NMR (101 MHz, DMSO-*d*_6_) *δ* ppm: 164.5 (C triazolopyrimidine), 155.6 (C triazolopyrimidine), 154.4 (CH triazolopyrimidine), 145.6 (C triazolopyrimidine), 144.8 (C pyrazole), 130.7 (Ar–C), 129.0 (Ar–C), 128.9 (Ar–C), 126.8 (Ar–C), 126.2 (Ar–CH), 124.5 (Ar–CH), 122.8 (Ar–CH), 122.7 (Ar–CH), 89.5 (CH triazolopyrimidine), 61.0 (CH pyrazole), 40.6 (CH_2_ pyrazole), 24.8 (CH_3_). Analysis calcd for C_21_H_18_ClN_7_ : C, 62.40; H, 4.46; N, 24.27. Found: C, 62.64; H, 4.53; N, 24.41. MS (EI^+^), *m*/*z*: 401.62 [M^+^].

##### 2.1.3.2 *N*-(4-(5-(2-chlorophenyl)-4,5-dihydro-1*H*-pyrazol-3-yl)phenyl)-5-methyl[1,2,4]triazolo[1,5-*a*]pyrimidin-7-amine(6b)

Yellow crystals (65% yield). M.p. 148–150 °C. IR: *ν*_max_/cm^−1^; 3324, 3226, 3066, 2923, 1681, 1556, 1506, 1368, 1316. ^1^H NMR (400 MHz, DMSO-*d*_6_) *δ* ppm: 10.22 (s, 1H, NH, exchangeable with D_2_O), 8.49 (s, 1H, C_2_–H of triazolopyrimidine), 7.71 (d, *J* = 7.6 Hz, 2H, Ar–H), 7.68 (s, 1H, NH, exchangeable with D_2_O), 7.57 (d, *J* = 6.7 Hz, 2H, Ar–H), 7.52–7.42 (m, 2H, Ar–H), 7.37–7.31 (m, 2H, Ar–H), 6.46 (s, 1H, C_6_–H of triazolopyrimidine), 5.17–5.12 (m, 1H, C_5_–H of pyrazole), 3.60 (dd, *J* = 16.4, 11.2 Hz, 1H, C_4_-H_A_H_B_ of pyrazole), 2.78 (dd, *J* = 16.4, 9.6 Hz, 1H, C_4_-H_A_H_B_ of pyrazole), 2.42 (s, 3H, CH_3_). ^13^C NMR (101 MHz, DMSO-*d*_6_) *δ* ppm: 155.6 (C triazolopyrimidine), 154.6 (C triazolopyrimidine), 148.5 (CH triazolopyrimidine), 147.9 (C triazolopyrimidine), 130.9 (C pyrazole), 130.7 (Ar–C), 129.6 (Ar–C), 129.1 (Ar–C), 127.9 (Ar–C), 127.6 (Ar–CH), 126.7 (Ar–CH), 125.1 (Ar–CH), 124.7 (Ar–CH), 124.2 (Ar–CH), 122.9 (Ar–CH), 89.7 (CH triazolopyrimidine), 60.6 (CH pyrazole), 41.0 (CH_2_ pyrazole), 24.9 (CH_3_). Analysis calcd for C_21_H_18_ClN_7_: C, 62.40; H, 4.46; N, 24.27. Found: C, 62.63; H, 4.57; N, 24.53. MS (EI^+^), *m*/*z*: 403.06 [M^+^].

##### 2.1.3.3 *N*-(4-(5-(2,4-dichlorophenyl)-4,5-dihydro-1*H*-pyrazol-3-yl)phenyl)-5-methyl-[1,2,4]triazolo[1,5-*a*]pyrimidin-7-amine(6c)

Yellow crystals (60% yield). M.p. 220–222 °C. IR: *ν*_max_/cm^−1^; 3337, 3270, 3091, 2923, 1620, 1580, 1559, 1373, 1337. ^1^H NMR (400 MHz, DMSO-*d*_6_) *δ* ppm: 10.26 (s, 1H, NH, exchangeable with D_2_O), 8.50 (s, 1H, C_2_–H of triazolopyrimidine), 7.71–7.69 (m, 3H, Ar–H), 7.64 (s, 1H, NH, exchangeable with D_2_O), 7.58 (d, *J* = 8.1 Hz, 1H, Ar–H), 7.47–7.45 (m, 3H, Ar–H), 6.46 (s, 1H, C_6_–H of triazolopyrimidine), 5.13–5.08 (m, 1H, C_5_–H of pyrazole), 3.61 (dd, *J* = 16.2, 11.1 Hz, 1H, C_4_-H_A_H_B_ of pyrazole), 2.78 (dd, *J* = 16.2, 9.7 Hz, 1H, C_4_-H_A_H_B_ of pyrazole), 2.42 (s, 3H, CH_3_). ^13^C NMR (101 MHz, DMSO-*d*_6_) *δ* ppm: 164.9 (C triazolopyrimidine), 156.0 (C triazolopyrimidine), 154.9 (CH triazolopyrimidine), 148.3 (C triazolopyrimidine), 146.0 (C pyrazole), 140.1 (Ar–CH), 137.3 (Ar–C), 133.2 (Ar–C), 132.9 (Ar–C), 130.9 (Ar–C), 129.7 (Ar–C), 129.2 (Ar–CH), 128.1 (Ar–CH), 127.1 (Ar–CH), 124.5 (Ar–CH), 90.0 (CH triazolopyrimidine), 60.5 (CH pyrazole), 40.9 (CH_2_ pyrazole), 25.2 (CH_3_). Analysis calcd for C_21_H_17_Cl_2_N_7_: C, 57.49; H, 3.88; N, 22.36. Found: C, 57.68; H, 4.01; N, 22.62. MS (EI^+^), *m*/*z*: 438.51 [M^+^].

##### 2.1.3.4 5-Methyl-*N*-(4-(5-(4-nitrophenyl)-4,5-dihydro-1*H*-pyrazol-3-yl)phenyl)-[1,2,4]triazolo[1,5-*a*]pyrimidin-7-amine(6d)

Yellow crystals (67% yield). M.p. 153–155 °C. IR: *ν*_max_/cm^−1^; 3560, 3233, 3073, 2840, 1622, 1589, 1560, 1509, 1367, 1316. ^1^H NMR (400 MHz, DMSO-*d*_6_) *δ* ppm: 10.19 (s, 1H, NH, exchangeable with D_2_O), 8.50 (s, 1H, C_2_–H of triazolopyrimidine), 8.24 (d, *J* = 7.9 Hz, 2H, Ar–H), 7.80 (s, 1H, NH, exchangeable with D_2_O), 7.71 (d, *J* = 8.5 Hz, 2H, Ar–H), 7.68 (d, *J* = 8.5 Hz, 2H, Ar–H), 7.48 (d, *J* = 7.9 Hz, 2H, Ar–H), 6.47 (s, 1H, C_6_–H of triazolopyrimidine), 5.07–5.01 (m, 1H, C_5_–H of pyrazole), 3.58 (dd, *J* = 16.0, 11.8 Hz, 1H, C_4_-H_A_H_B_ of pyrazole), 2.90 (dd, *J* = 16.0, 10.0 Hz, 1H, C_4_-H_A_H_B_ of pyrazole), 2.43 (s, 3H, CH_3_). ^13^C NMR (101 MHz, DMSO-*d*_6_) *δ* ppm: 164.9 (C triazolopyrimidine), 156.0 (C triazolopyrimidine), 155.9 (CH triazolopyrimidine), 154.9 (C triazolopyrimidine), 151.5 (C pyrazole), 148.6 (Ar–C), 147.1 (Ar–C), 145.9 (Ar–C), 130.9 (Ar–C), 128.4 (Ar–CH), 127.0 (Ar–CH), 124.5 (Ar–CH), 124.1 (Ar–CH), 90.0 (CH triazolopyrimidine), 63.4 (CH pyrazole), 41.1 (CH_2_ pyrazole), 25.2 (CH_3_). Analysis calcd for C_21_H_18_N_8_O_2_: C, 60.81; H, 4.34; N, 27.03. Found: C, 61.04; H, 4.51; N, 27.29. MS (EI^+^), *m*/*z*: 414.45 [M^+^].

##### 2.1.3.5 *N*-(4-(5-(4-bromophenyl)-4,5-dihydro-1*H*-pyrazol-3-yl)phenyl)-5-methyl-[1,2,4]triazolo[1,5-*a*]pyrimidin-7-amine(6e)

Yellow crystals (63% yield). M.p. 132–134 °C. IR: *ν*_max_/cm^−1^; 3213, 2921, 1621, 1587, 1556, 1368, 1316, 1007, 770. ^1^H NMR (400 MHz, DMSO-*d*_6_) *δ* ppm: 10.22 (s, 1H, NH, exchangeable with D_2_O), 8.50 (s, 1H, C_2_–H of triazolopyrimidine), 7.71–7.69 (m, 3H, 2 Ar–H and NH, exchangeable with D_2_O), 7.55 (d, *J* = 7.0 Hz, 2H, Ar–H), 7.47 (d, *J* = 7.0 Hz, 2H, Ar–H), 7.35 (d, *J* = 7.6 Hz, 2H, Ar–H), 6.47 (s, 1H, C_6_–H of triazolopyrimidine), 4.88–4.84 (m, 1H, C_5_–H of pyrazole), 3.48 (dd, *J* = 15.5, 11.3 Hz, 1H, C_4_-H_A_H_B_ of pyrazole), 2.85 (dd, *J* = 15.5, 10.5 Hz, 1H, C_4_-H_A_H_B_ of pyrazole), 2.43 (s, 3H, CH_3_). ^13^C NMR (101 MHz, DMSO-*d*_6_) *δ* ppm: 164.3 (C triazolopyrimidine), 155.6 (C triazolopyrimidine), 154.3 (CH triazolopyrimidine), 148.0 (C triazolopyrimidine), 145.6 (C pyrazole), 142.6 (Ar–C), 137.0 (Ar–C), 131.3 (Ar–C), 130.5 (Ar–C), 128.9 (Ar–CH), 126.5 (Ar–CH), 124.0 (Ar–CH), 120.1 (Ar–CH), 89.5 (CH triazolopyrimidine), 63.0 (CH pyrazole), 40.5 (CH_2_ pyrazole), 24.8 (CH_3_). Analysis calcd for C_21_H_18_BrN_7_: , 56.21; H, 4.01; N, 21.86. Found: C, 56.35; H, 4.20; N, 22.04. MS (EI^+^), *m*/*z*: 448.39 [M^+^].

##### 2.1.3.6 5-Methyl-*N*-(4-(5-(thiophen-2-yl)-4,5-dihydro-1*H*-pyrazol-3-yl)phenyl)-[1,2,4]triazolo[1,5-*a*]pyrimidin-7-amine(6f)

Yellow crystals (65% yield). M.p. 118–120 °C. IR: *ν*_max_/cm^−1^; 3262, 3087, 2923, 1669, 1620, 1556, 1505, 1367, 1315. ^1^H NMR (400 MHz, DMSO-*d*_6_) *δ* ppm: 10.26 (s, 1H, NH, exchangeable with D_2_O), 8.51 (s, 1H, C_2_–H of triazolopyrimidine), 7.73–7.71 (m, 3H, 2H Ar–H and 1H NH exchangeable with D_2_O), 7.48 (d, *J* = 8.6 Hz, 2H, Ar–H), 7.42 (dd, *J* = 5.1, 1.9 Hz, 1H, thienyl-H), 7.08 (d, *J* = 3.5 Hz, 1H, thienyl-H), 6.99 (dd, *J* = 5.1, 3.5 Hz, 1H, thienyl-H), 6.48 (s, 1H, C_6_–H of triazolopyrimidine), 5.16–5.10 (m, 1H, C_5_–H of pyrazole), 3.49 (dd, *J* = 16.3, 10.5 Hz, 1H, C_4_-H_A_H_B_ of pyrazole), 2.92 (dd, *J* = 16.3, 10.2 Hz, 1H, C_4_-H_A_H_B_ of pyrazole), 2.43 (s, 3H, CH_3_). ^13^C NMR (101 MHz, DMSO-*d*_6_) *δ* ppm: 164.9 (C triazolopyrimidine), 156.0 (C triazolopyrimidine), 154.9 (CH triazolopyrimidine), 149.1 (C triazolopyrimidine), 147.1 (C pyrazole), 145.9 (Ar–C), 137.3 (Ar–C), 131.0 (thienyl-C), 127.2 (Ar–CH), 127.0 (thienyl-CH), 125.2 (thienyl-CH), 125.1 (thienyl-CH), 124.5 (Ar–CH), 90.0 (CH triazolopyrimidine), 59.9 (CH pyrazole), 41.8 (CH_2_ pyrazole), 25.2 (CH_3_). Analysis calcd for C_19_H_17_N_7_S : C, 60.73; H, 4.53; N, 26.10. Found: C, 61.02; H, 4.67; N, 26.23. MS (EI^+^), *m*/*z*: 375.73 [M^+^].

##### 2.1.3.7 5-Methyl-*N*-(4-(5-(naphthalen-1-yl)-4,5-dihydro-1*H*-pyrazol-3-yl)phenyl)-[1,2,4]triazolo[1,5-*a*]pyrimidin-7-amine(6g)

Yellow crystals (55% yield). M.p. 225–227 °C. IR: *ν*_max_/cm^−1^; 3261, 3056, 2923, 1621, 1587, 1555, 1505, 1368, 1315. ^1^H NMR (400 MHz, DMSO-*d*_6_) *δ* ppm: 10.23 (s, 1H, NH, exchangeable with D_2_O), 8.50 (s, 1H, C_2_–H of triazolopyrimidine), 8.18 (d, *J* = 7.8 Hz, 1H, naphthyl-H), 7.96 (d, *J* = 7.3 Hz, 1H, naphthyl-H), 7.85 (d, *J* = 8.0 Hz, 1H, naphthyl-H), 7.77–7.69 (m, 3H, 2H Ar–H and 1H NH exchangeable with D_2_O), 7.68 (d, *J* = 7.3 Hz, 1H, naphthyl-H), 7.62–7.40 (m, 5H, 2H Ar–H and 3H naphthyl-H), 6.45 (s, 1H, C_6_–H of triazolopyrimidine), 5.64–5.59 (m, 1H, C_5_–H of pyrazole), 3.77 (dd, *J* = 16.0, 11.4 Hz, 1H, C_4_-H_A_H_B_ of pyrazole), 2.85 (dd, *J* = 16.0, 10.2 Hz, 1H, C_4_-H_A_H_B_ of pyrazole), 2.42 (s, 3H, CH_3_). ^13^C NMR (101 MHz, DMSO-*d*_6_) *δ* ppm: 164.9 (C triazolopyrimidine), 156.0 (C triazolopyrimidine), 154.9 (CH triazolopyrimidine), 148.3 (C triazolopyrimidine), 146.0 (C pyrazole), 139.1 (Ar–C), 137.0 (Ar–C), 134.0 (Ar–C), 131.2 (Ar–C), 131.0 (Ar–CH), 129.2 (Ar–CH), 128.0 (Ar–CH), 126.9 (Ar–CH), 126.6 (Ar–CH), 126.2 (Ar–CH), 126.0 (Ar–CH), 124.5 (Ar–CH), 124.1 (Ar–C), 123.6 (Ar–CH), 90.0 (CH triazolopyrimidine), 61.0 (CH pyrazole), 40.9 (CH_2_ pyrazole), 25.2 (CH_3_). Analysis calcd for C_25_H_21_N_7_: C, 71.52; H, 5.00; N, 23.36. Found: C, 71.35; H, 4.89; N, 23.52. MS (EI^+^), *m*/*z*: 419.19 [M^+^].

### 2.2. Biological activity

#### 2.2.1. *In vitro* inhibition assay of COX-1 and COX-2

The activity of the target compounds in inhibiting cyclooxygenase enzymes was evaluated using ovine COX-1 and human recombinant COX-2. According to the fluorometric assay protocol illustrated in BioVision's COX-1 inhibitor screening kit leaflet (Catalog #K548 100, BioVision, Milpitas, California, USA) and the COX-2 inhibitor screening kit leaflet (Catalog #K547 100, BioVision, Milpitas, California, USA).^28,29^

To ensure a direct, molecule-for-molecule potency comparison, all synthesized target compounds and reference drugs were normalized to identical molar concentrations and tested across a final in-well concentration range of 0.001 to 10 µM. The step-by-step procedure is detailed extensively in SI Material.

#### 2.2.2. *In vivo* anti-inflammatory activity

The acute carrageenan-induced paw oedema standard method in rats was utilized to evaluate the *in vivo* anti-inflammatory effects of the tested compounds.^30^

#### 2.2.3. *In vitro* inhibition assay of 5-LOX

The 5-LOX suppressive effect was measured using a recombinant human 5-LOX enzyme screening kit (Catalog #K980 100, BioVision, Milpitas, California, USA), adhering to the manufacturer's manual.^28^ (Detailed procedure in SI Material).

#### 2.2.4. ROS scavenging assay

The scavenging activity of the superoxide anion free radical was measured by the pyrogallol autoxidation method. The compounds were evaluated across a range of concentrations to uncover their potential effects, and the change in absorbance was utilized to calculate the percentage of scavenging activity.^31,32^

#### 2.2.5. Inhibition of IL-6 production

The Colorimetric mouse IL-6 ELISA kit (Abcam, ab100712) was used to investigate the inhibitory potential of the tested compounds on the production of IL-6 in lipopolysaccharide (LPS)-activated RAW 264.7 macrophages. The level of the inflammatory mediator IL-6 was measured in LPS-induced RAW 264.7 macrophage cells in the absence and presence of the selected compounds and the reference drug.^33^

#### 2.2.6. Inhibition of TNF-α production

The concentration of TNF-α in the culture supernatants of LPS-stimulated RAW264.7 macrophages was quantified using the Mouse TNF-α SimpleStep ELISA Kit (ab208348, Abcam, Cambridge, UK) according to the manufacturer's instructions.^34,35^

#### 2.2.7. Cell viability assay (MTT assay)

The cytotoxicity of the tested compounds at different concentrations was assessed using the MTT assay on RAW264.7 macrophages.^36^

#### 2.2.8. Statistical analysis

All fluorometric enzymatic inhibition assays were performed in independent triplicates (*n* = 3). The half-maximal inhibitory concentrations (IC_50_) were calculated using non-linear regression analysis (sigmoidal dose–response curve with variable slope) and are reported as Mean ± Standard Deviation (SD). To validate the potency of the synthesized molecules, statistical significance between the IC_50_ values of the test compounds and the standard reference drugs (diclofenac, celecoxib, and zileuton) was determined using a one-way analysis of variance (ANOVA) followed by Dunnett's multiple comparison test with the significance level set at 0.05, denoted by corresponding significance symbols (*, #, •) within the results tables.

### 2.3. *In silico* study

#### 2.3.1. Molecular modeling

The RCSB PDB supplied the 3D structures of two target enzymes, COX-2 protein (1CX2 (ref. 37)) and the 5-LOX protein (6n2w^38^) in PDB format. AutoDock Vina was used to investigate binding affinities and forecast protein-ligand interactions.^39^

The molecular docking protocol was validated by redocking the co-crystallized ligands (SC-558 and natural inhibitor NDGA) into their respective active sites (COX-2 and 5-LOX, respectively). The reproduced binding modes yielded Root Mean Square Deviation (RMSD) values well below the acceptable threshold of 2.0 Å (specifically 0.56 and 1.97 Å, respectively), confirming the reliability of the binding parameters. Comprehensive details, metrics, and overlay figures for this benchmarking are provided in the SI Material.

#### 2.3.2. Molecular dynamics simulation

CHARMM-GUI solution builder^40,41^ was utilized to generate the requisite input files for COX-2. Force field parameters and molecular topologies for the selected ligands were assigned using the CGenFF server.^42,43^

#### 2.3.3. Drug-likeness, physicochemical properties

The molecular structures of the synthesized compounds were converted to Simplified Molecular Input Line Entry System (SMILES) format to enable comprehensive evaluation of their ADMET properties. The evaluation was carried out using the online interface available at https://www.swissadme.ch/.^44^

#### 2.3.4. *In silico* toxicology

Protox-3 (ref. 45) and ADMETlab 3.0 (ref. 46) online prediction platforms were utilized to predict the *in silico* toxicity profile of the synthesized compounds. Chemical structures were converted into SMILES format and uploaded to the server. The analysis included the prediction of acute oral toxicity (LD_50_), organ toxicity, and toxicological endpoints.

## 3 Results and discussion

### 3.1. Chemistry

The synthetic pathway used to prepare 5a–g and 6a–g is illustrated in Schemes 1 and 2. The synthesis of 5-methyl-[1,2,4]triazolo[1,5-*a*]pyrimidin-7(4*H*)-one (2) and 7-chloro-5-methyl-[1,2,4]triazolo[1,5-*a*]pyrimidine (3) were carried out starting from 4*H*-1,2,4-triazol-3-amine (1) according to the reported procedures.^27^ The nucleophilic substitution reaction of 3 with *p*-aminoacetophenone produced the starting compound 4 in a good yield, which, in turn, underwent base-catalyzed aldol condensation with different aldehydes to afford the targeted chalcones 5a–g (Scheme 1). The resulting chalcones were synthesized in *E* configuration as indicated by the high coupling constant (*J* = 15.2–15.9 Hz) of the two doublets corresponding to vinylic protons of the propenone linkage.

**Scheme 1:**
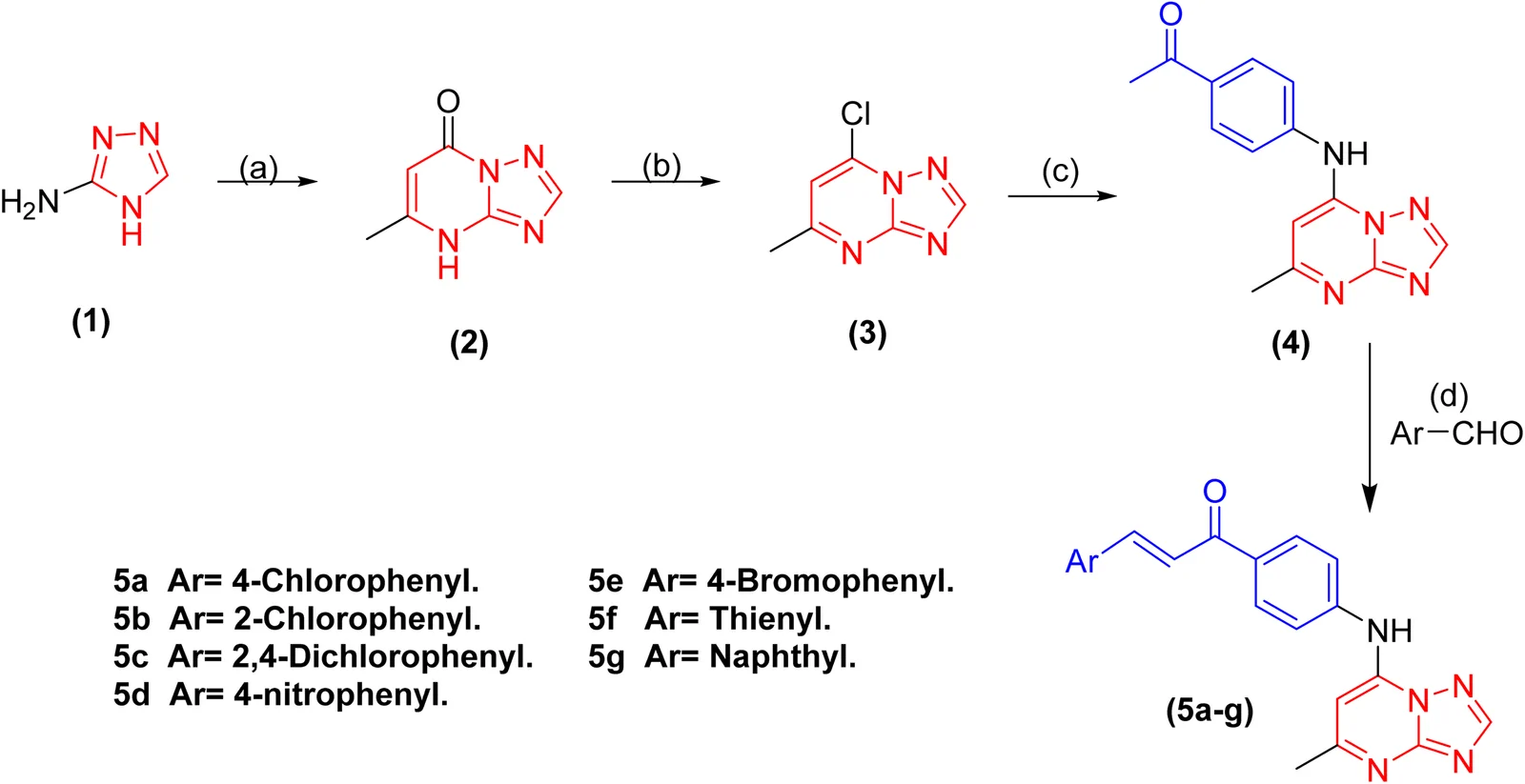
Synthesis of chalcone derivatives 5a–g. Reagents and conditions: (a) ethyl acetoacetate, acetic acid, reflux 6 h. (b) excess POCl_3_, reflux 5 h. (c) *p*-aminoacetophenone, ethanol, TEA, rt, 5 h. (d) aromatic aldehyde derivatives, ethanol, KOH, rt.

**Scheme 2:**
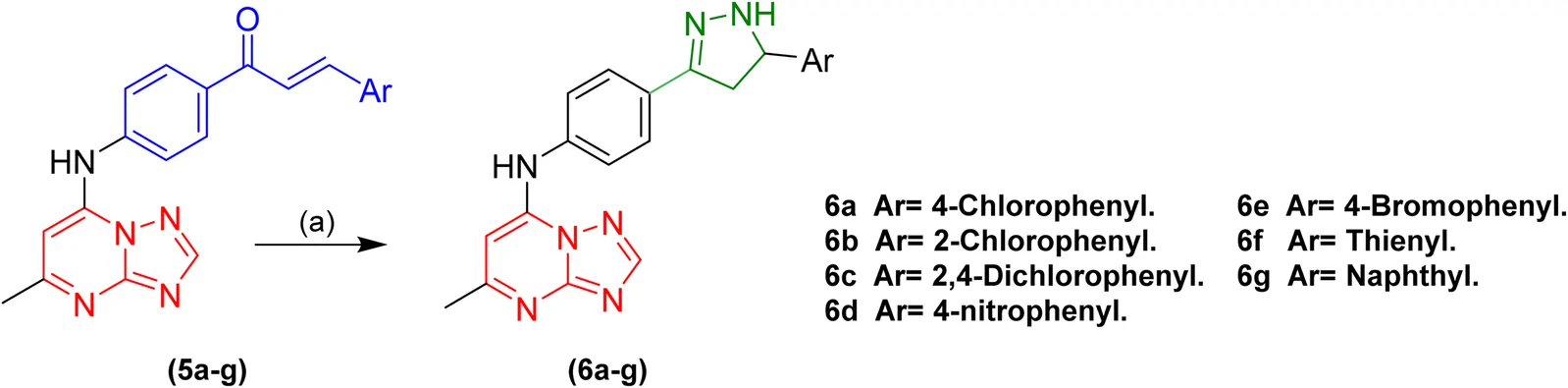
Synthesis of 4,5-dihydro-1*H*-pyrazole derivatives 6a–g. Reagents and conditions: (a) NH_2_NH_2_ H_2_O, rt, 24 h.

The reaction of chalcones 5a–g with hydrazine hydrate gave the corresponding 4,5-dihydro-1*H*-pyrazoles 6a–g (Scheme 2).

The ^1^H NMR supported the ABX system of pyrazoline ring protons. While each of HA and HB appeared as a doublet of doublets at ≈ 2.8 and 3.6 ppm, respectively, HX appeared as a multiple peak at ≈ 5 ppm (see Fig. S1 in the SI Materials).

### 3.2. Biological activity

#### 3.2.1. *In vitro* inhibition assay of COX-1 and COX-2

Chalcone series 5a–g and pyrazole series 6a–g were examined for their potential activity against COX enzymes with celecoxib and diclofenac sodium as positive controls. The screening results were expressed as IC_50_ (µM), and the selectivity towards COX-2 was determined as COX-1 IC_50_/COX-2 IC_50_. Table 1 summarizes the results, revealing that both series demonstrated a promising suppressive effect against both enzymes. The title compounds 5a, e, and 6c, d, f exhibited superior COX-2 inhibition activity compared to diclofenac sodium.

**Table 1:** Evaluation of inhibitory activities against COX-1 and COX-2 enzymes and selectivity indices of the synthesized triazolo[1,5-*a*]pyrimidine derivatives and reference compounds^a^

Compound	bCOX-1 IC_50_(µM)	bCOX-2 IC_50_(µM)	cCOX-2 SI
5a	17.5 ± 0.5	1.361 ± 0.05*	12.86
5b	34.02 ± 1.25^#^*	12.28 ± 0.4^#^*	2.77
5c	8.127 ± 0.3^#^	40.44 ± 1.33^#^*	0.201
5d	3.709 ± 0.11^#^*	5.49 ± 0.19^#^	0.676
5e	19.55 ± 0.72	3.42 ± 0.11	5.716
5f	340 ± 12.4^#^*	110.7 ± 3.64^#^*	3.071
5g	79.21 ± 2.27^#^*	23.36 ± 0.8^#^*	3.391
6a	39.08 ± 1.43^#^*	16.15 ± 0.53^#^*	2.42
6b	12.99 ± 0.37	53.86 ± 1.85^#^*	0.241
6c	45.79 ± 1.68^#^*	2.537 ± 0.08	18.05
6d	11.03 ± 0.4^#^	1.237 ± 0.04*	8.917
6e	22.03 ± 0.63*	8.002 ± 0.28^#^*	2.753
6f	1.11 ± 0.03^#^*	2.752 ± 0.09	0.403
6g	34.08 ± 1.25^#^*	24.63 ± 0.81^#^*	1.384
Celecoxib	19.67 ± 0.72	0.799 ± 0.03	24.62
Diclofenac	12.28 ± 0.45	4.812 ± 0.16	2.552

a ^#^ *p* < 0.05 *vs.* celecoxib. *p < 0.05 *vs.* Diclofenac.

b IC_50_ in (µM) concentration is calculated from three independent replicates and is presented as the mean ± SD.

c Selectivity index is calculated as COX-1 IC_50_/COX-2 IC_50_.

The pyrazole derivative 6d demonstrated the highest potency against COX-2 (IC_50_ = 1.237 µM, SI = 8.917), approximately 4-fold more potent and selective than diclofenac sodium. This could provide a potential advantage for 6d in reducing the risk of gastrointestinal complications associated with COX-2 non-selective inhibitors. Furthermore, pyrazole 6c showed the maximum affinity for COX-2 (SI = 18.05). Moreover, derivatives 5a and 6d showed a comparable COX-2 potency and milder selectivity (5a; IC_50_ COX-2 = 1.361 µM, SI = 12.86. 6d; IC_50_ COX-2 = 1.237 µM, SI = 8.917) compared to the standard COX-2 selective inhibitor celecoxib (IC_50_ COX-2 = 0.799 µM, SI = 24.62). The mild selectivity of COX-2 in compounds 5a and 6d, while retaining promising potency against COX-2, may help reduce harmful cardiovascular events often seen with highly selective COX-2 inhibitors.

According to the assessment outcomes, several structure–activity relationships (SARs) have been identified (Fig. 5). Compounds with *para*-chloro-substitution in both series (5a, and 6a) had better COX-2 potency and selectivity (5a; IC_50_ COX-2 = 1.361 µM, SI = 12.86, 6a; IC_50_ COX-2 = 16.15 µM, SI = 2.42) compared to *ortho*-chloro-substituted analogues (5b, and 6b) (5b; IC_50_ COX-2 = 12.28 µM, SI = 2.77, 6b; IC_50_ COX-2 = 53.86 µM, SI = 0.241). The mono-halogenated phenyl derivatives in the chalcone series (5a, b, e) showed a favorable COX-2 inhibition potential and selectivity (5a; IC_50_ COX-2 = 1.361 µM, SI = 12.86, 5b; IC_50_ COX-2 = 12.28 µM, SI = 2.77, 5e; IC_50_ COX-2 = 3.42 µM, SI = 5.716) when compared to their analogues in the pyrazole series namely 6a, b, e (6a; IC_50_ COX-2 = 16.15 µM, SI = 2.42, 6b; IC_50_ COX-2 = 53.86 µM, SI = 0.241, 6e; IC_50_ COX-2 = 8.002 µM, SI = 2.753). On the other hand, the di-halogenated phenyl derivative in the pyrazole series (6c) had better COX-2 inhibition and selectivity (6c; IC_50_ COX-2 = 2.537 µM, SI = 18.05) than its corresponding chalcone derivative 5c (5c; IC_50_ COX-2 = 40.44 µM, SI = 0.201).

**Fig. 5:**
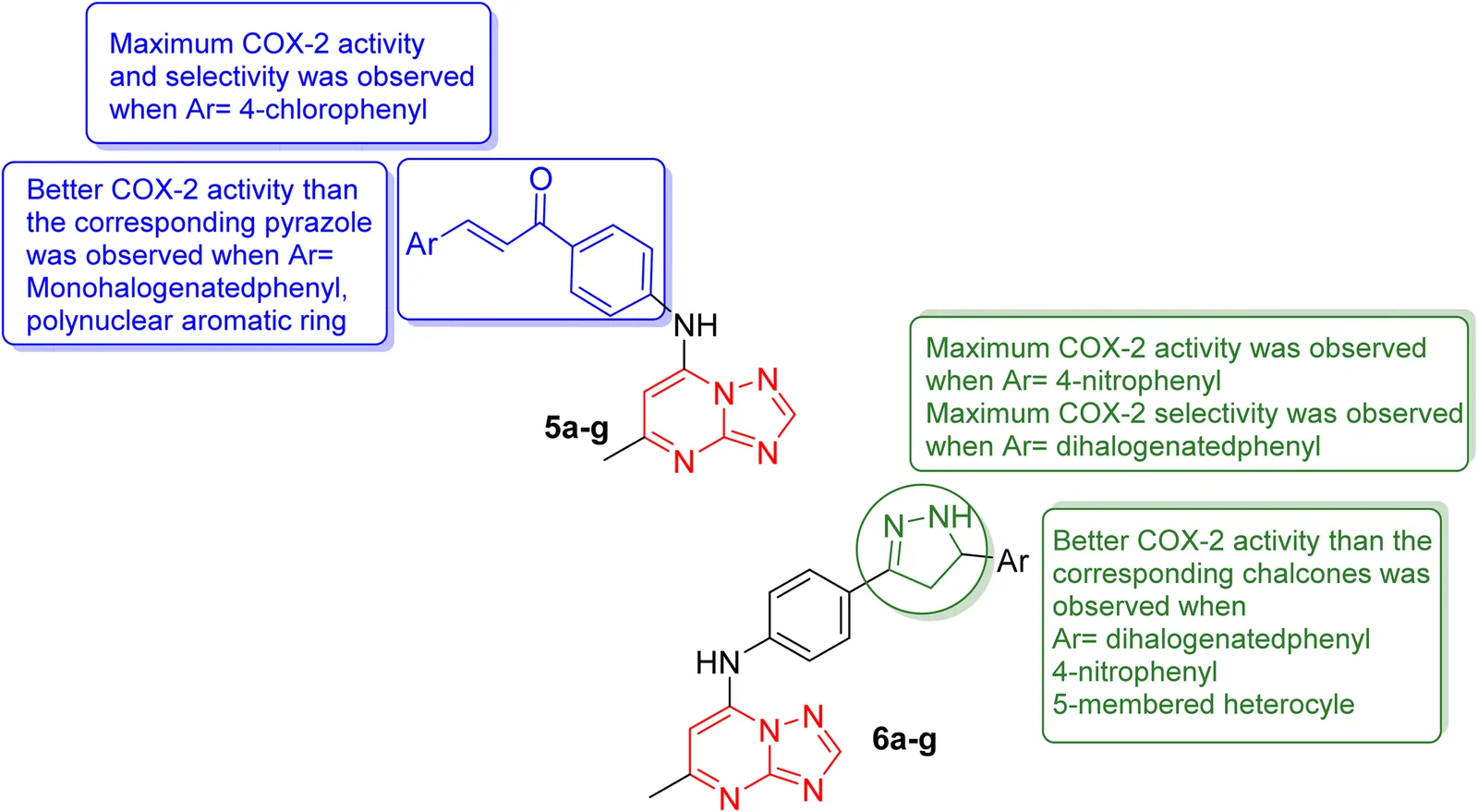
General representation of SAR analysis for the newly synthesized COX-2 inhibitors.

Additionally, replacing the substituted phenyl groups in the pyrazole tail with a five-membered thienyl ring in 6f demonstrated better activity against COX-2 but lower selectivity (IC_50_ COX-2 = 2.752 µM, SI = 0.403) compared to the corresponding chalcone 5f (IC_50_ = 110.7 µM, SI = 3.071). Furthermore, replacing the substituted phenyl groups in the chalcone tail with a naphthalene in 5g demonstrated better COX-2 activity and selectivity (IC_50_ COX-2 = 23.36 µM, SI = 3.391) compared to the corresponding pyrazole 6g (IC_50_ = 24.63 µM, SI = 1.384).

A detailed structural rationalization of these electronic and steric differences, backed by energy scores, is fully elucidated in the subsequent Molecular Docking Section.

#### 3.2.2. *In vivo* anti-inflammatory activity

The *in vivo* anti-inflammatory effects of compounds 5a, 6c, and 6d, identified as the most potent and selective derivatives from previous *in vitro* evaluations (Table 1), were assessed using the carrageenan-induced rat paw oedema model.^30^ The results are presented in Table 2.

**Table 2:** Anti-inflammatory properties at 10 mg kg^−1^ (rat body weight) diclofenac sodium mol equivalent^a^

Compounds	Mean oedema thickness “mm” (% inhibition of oedema ± SE) with % inhibition (±SE) in parentheses			
	2 h	3 h	4 h	24 h
Control	0.717 ± 0.03 (0.00 ± 0.00)	0.833 ± 0.04 (0.00 ± 0.00)	0.956 ± 0.07 (0.00 ± 0.00)	0.633 ± 0.08 (0.00 ± 0.00)
Diclofenac sodium	0.567 ± 0.02** (20.9 ± 1.5)	0.489 ± 0.01** (41.3 ± 1.8)	0.411 ± 0.01** (57.0 ± 2.0)	0.289 ± 0.01** (54.3 ± 1.7)
5a	0.600 ± 0.02* (16.3 ± 1.3)	0.567 ± 0.02** (31.9 ± 1.6)	0.556 ± 0.01** (41.8 ± 1.9)	0.308 ± 0.03** (51.3 ± 2.1)
6c	0.610 ± 0.01* (14.9 ± 1.2)	0.560 ± 0.03** (32.8 ± 1.7)	0.571 ± 0.01** (40.3 ± 1.5)	0.370 ± 0.05* (46.6 ± 1.8)
6d	0.633 ± 0.02 (11.7 ± 1.1)	0.556 ± 0.02** (33.3 ± 1.5)	0.511 ± 0.05** (46.6 ± 2.2)	0.344 ± 0.07** (45.7 ± 2.4)

a Statistical evaluations were carried out by one-way ANOVA *p < 0.05, **p < 0.001.

The tested compounds exhibited notable anti-inflammatory activity at various time points. The maximum anti-inflammatory effect of all compounds was observed between 3 and 4 hours post-administration, with % inhibition values of 41.8% (5a), 40.3% (6c), and 46.6% (6d), compared to 57.0% produced by diclofenac sodium. The tested compounds exhibited weak to moderate inhibition at early time points (2 h), with inhibition increasing over time, revealing their time-dependent anti-inflammatory potential. Overall, 5a, 6c, and 6d demonstrated promising activity comparable to diclofenac sodium, suggesting their potential as anti-inflammatory agents.

The relative potencies of the tested compounds compared to diclofenac sodium are presented in Fig. 6. Compound 5a showed consistently high potency across all time points, reaching 94.5% at 24 h, though it was slightly lower than diclofenac sodium at earlier time points (78.0% at 2 h, 77.2% at 3 h, and 73.3% at 4 h). Compound 6c exhibited moderate potency, ranging from 71.3% at 2 h to 85.8% at 24 h, with the highest relative activity at 3 h (79.4%). Compound 6d displayed a time-dependent increase in potency, starting at 56.0% at 2 h, and peaking at 81.8% at 4 h, maintaining 84.2% at 24 h. These results indicate that the three COX-2 inhibitors displayed a substantial anti-inflammatory effect comparable to diclofenac sodium.

**Fig. 6:**
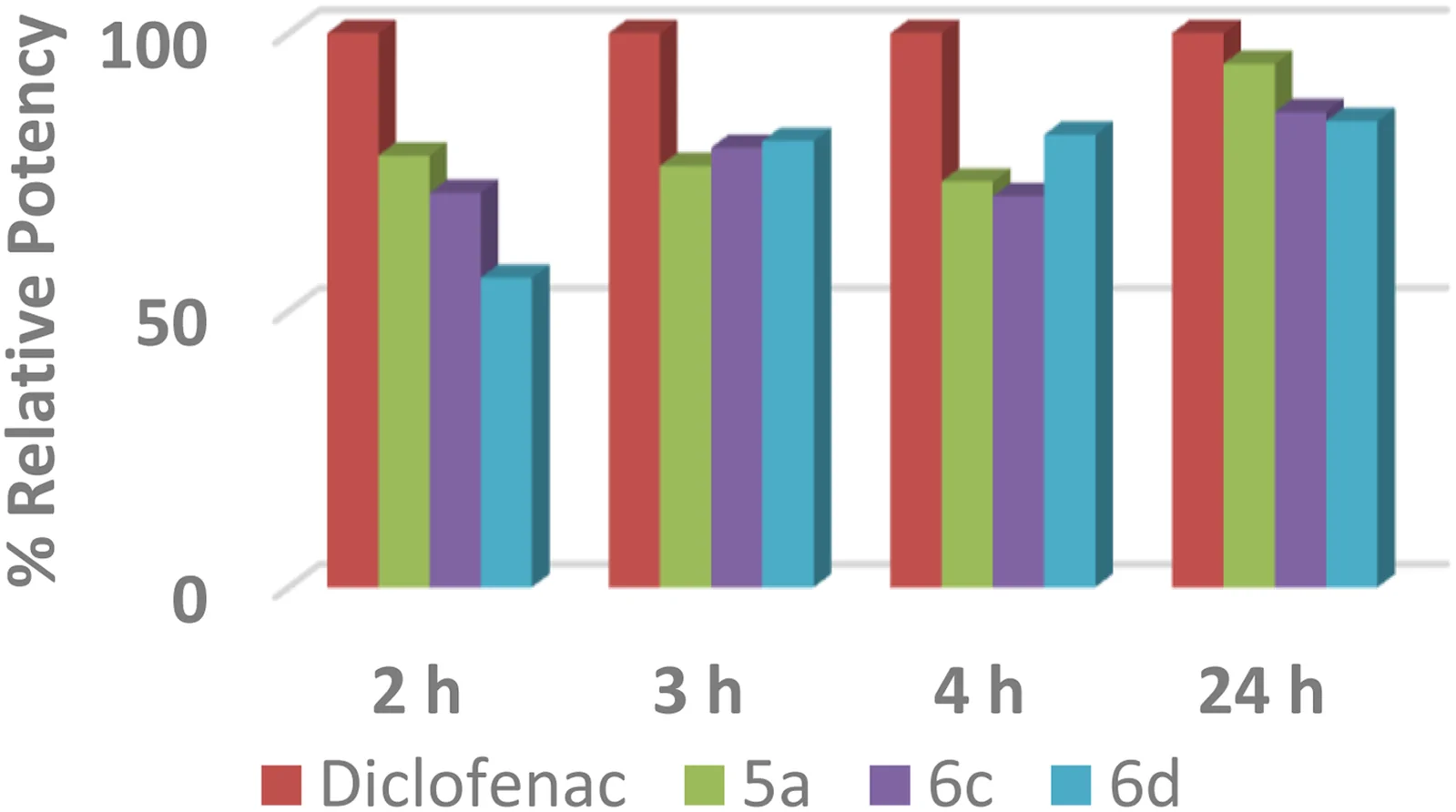
Relative potencies of the tested compounds compared to diclofenac sodium.

#### 3.2.3. *In vitro* inhibition assay of 5-LOX

5-Lipoxygenase (5-LOX) is a non-heme iron-containing dioxygenase that catalyzes the synthesis of epoxides (leukotrienes) from unsaturated fatty acids (arachidonic acid). It serves a critical function in the propagation of the inflammatory process and is overexpressed in a variety of cancer cells. Therefore, developing a 5-LOX inhibitor with therapeutic potential for a variety of inflammatory diseases becomes necessary.^8^ We further estimated the inhibitory potential of promising compounds 5a, 6c, and 6d against 5-LOX using zileuton as a reference. The screening results are expressed in Table 3. The results revealed that pyrazole 6d and chalcone 5a exhibited significant inhibition of the 5-LOX enzyme (IC_50_ = 1.031 µM and 1.706 µM, respectively) relative to zileuton, which had an IC_50_ of 0.386 µM.

**Table 3:** 5-LOX suppressive activity and ROS production of the targeted triazolopyrimidines and references^a^

Compound	5-LOX IC_50_ (µM)	ROS scavenging capacity IC_50_ (µM)
5a	1.706 ± 0.055^#^	64.02 ± 2.4*^•^
6c	3.222 ± 0.104^#^	131.36 ± 4.9*^•^
6d	1.031 ± 0.033^#^	34.45 ± 1.3*^•^
Zileuton	0.386 ± 0.012	—
Celecoxib	—	122.19 ± 4.5
Diclofenac	—	89.59 ± 3.3

a IC_50_ in (µM) concentration is calculated from three independent replicates and is presented as the mean ± SD. ^#^*p* < 0.05 *vs.* Zileuton. *p < 0.05 *vs.* celecoxib. ^•^*p* < 0.05 *vs.* diclofenac.

#### 3.2.4. ROS scavenging assay

Reactive oxygen species (ROS) have been implicated in the genesis and progression of various inflammatory diseases and conditions, such as arthritis, atherosclerosis, cancer, heart attack, and Alzheimer's disease, by inducing oxidative stress.^32^ So, there is a need for antioxidant potential to protect against oxidative stress. Compounds 5a, 6c, and 6d were investigated for their free radical scavenging activity by the superoxide radical scavenging assay.^31,32^ The findings are detailed in Table 3. 6d and 5a demonstrated remarkable inhibitory activity with IC_50_ values of 34.45 µM and 64.02 µM, respectively, surpassing the reference celecoxib (IC_50_ = 122.19 µM) and diclofenac sodium (IC_50_ = 89.59 µM). Moreover, 6c exhibited comparable inhibitory potential to celecoxib, with an IC_50_ value of 131.36 µM.

Compounds with chalcone and pyrazoline fragments have been reported to exhibit antioxidant activity.^47–49^ However, in many cases, the pyrazoline derivatives exhibited more antioxidant activity when compared to the corresponding chalcones.^50^ The mechanism of radical scavenging in the used pyrogallol autoxidation assay is based on proton transfer from the tested compound to neutralize the formed radicals and break the radical chain reaction.^51^ Thus, the NH group of pyrazole derivatives is essential for attenuating oxidative stress.^52^ The measured activity is influenced by both the kind of heterocyclic pharmacophore and the substituent present on the attached aromatic ring. The nitro group on the phenyl substituent in 6d, the most potent antioxidant, is involved in extended conjugation, which could lower the energy barrier for proton transfer and improve the antioxidant activity, as previously reported.^53^

#### 3.2.5. Inhibition of IL-6 production

IL-6 is an essential multifunctional proinflammatory cytokine involved in the pathogenesis of many inflammatory diseases. It contributes to the initiation and extension of the inflammatory process. Inflammatory stimuli activate COX-2, which converts arachidonic acid into prostaglandin *E*_2_ (PGE_2_), triggering IL-6 expression in macrophages and fibroblasts.^54,55^ So considered an important molecular target in designing anti-inflammatory drugs. To assess the anti-inflammatory efficacy of the most promising derivatives, we quantified their inhibitory effect on IL-6 production in lipopolysaccharide (LPS)-stimulated RAW 264.7 macrophages. The results are shown in Table 4. Chalcone 5a demonstrated potent IL-6 inhibition with a percentage of inhibition (84.6%) surpassing the standard drug diclofenac sodium (71.4%) and similar to celecoxib (85.8%). Meanwhile, pyrazole 6d exhibited the highest inhibition value (90.9%), exceeding both celecoxib and diclofenac sodium.

**Table 4:** *In vitro* suppression activity on LPS-induced IL-6 expression^a^

Compound	IL-6 (pg mL^−1^)	% Inhibition
5a	32.34 ± 1.11	84.6
6c	122.41 ± 4.19	41.9
6d	19.09 ± 0.65	90.9
Celecoxib	29.98 ± 1.02	85.8
Diclofenac	60.13 ± 2.06	71.4
LPS	210.52 ± 7.22	—

a Concentration is represented as the mean ± SD, *n* = 2. 
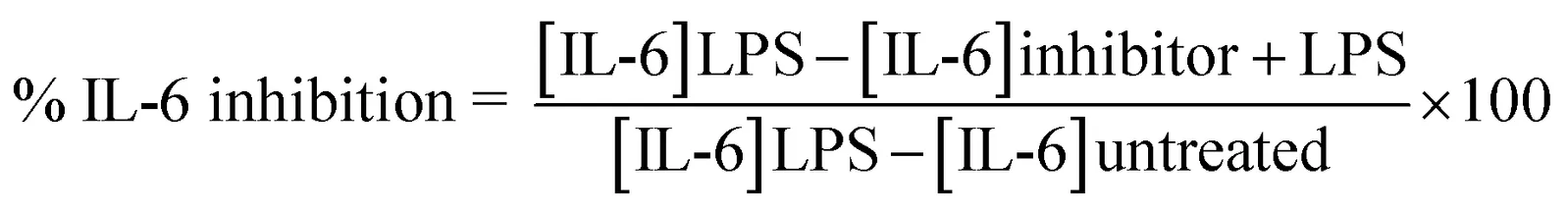

#### 3.2.6. Inhibition of TNF-α production

Since TNF-α is a pivotal mediator of inflammation, the inhibitory activity of the most active candidate against TNF-α generation in LPS-stimulated RAW 264.7 macrophages was evaluated to further assess their anti-inflammatory potential, and the screening results are represented in Table 5. Compound 6c showed comparable inhibition to diclofenac sodium, achieving 69.44% inhibition *versus* 75.75% for the reference drug. Moreover, compounds 5a and 6d displayed moderate inhibitory effects with inhibition percentages of 55.61% and 58.62%, respectively.

**Table 5:** *In vitro* suppression activity on LPS-induced TNF-α expression^a^

Compound	TNF-α (pg mL^−1^)	% Inhibition
5a	1010 ± 67.8*^•^	55.61
6c	695.7 ± 42.8^•^	69.44
6d	941.7 ± 133.7^•^	58.62
Diclofenac	551.7 ± 23.8^•^	75.75
LPS	2276 ± 46.3*	—

a Concentration is represented as the mean ± SD, *n* = 3. *p < 0.05 *vs.* diclofenac. ^•^*p* < 0.05 *vs.* LPS. 
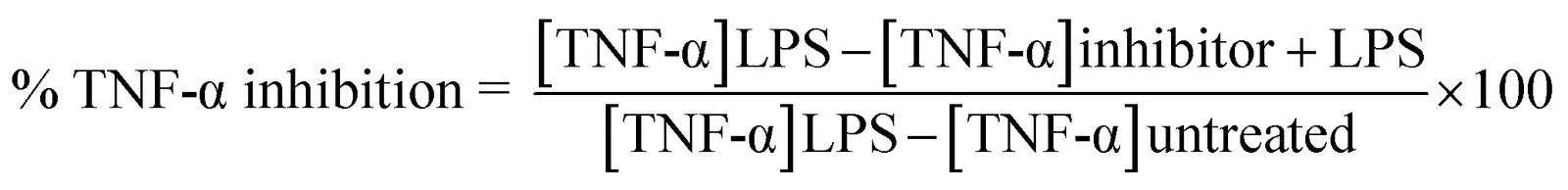

#### 3.2.7. Cell viability assay (MTT assay)

To assess whether the observed decrease in IL-6 and TNF-α levels is a genuine anti-inflammatory effect rather than simply a consequence of cytotoxicity, we measured the cytotoxicity of 5a, 6c, and 6d at different concentrations (1000 µM, 250 µM, 63 µM, 16 µM, and 4 µM) (Fig. 7). The cell viability assay was performed by the use of the MTT assay on RAW264.7 macrophages.^56^ The results show no cytotoxicity in RAW264.7 macrophages at 16 µM and 4 µM for both the positive control and tested compounds, confirming that the decrease in IL-6 levels is a true anti-inflammatory effect.

**Fig. 7:**
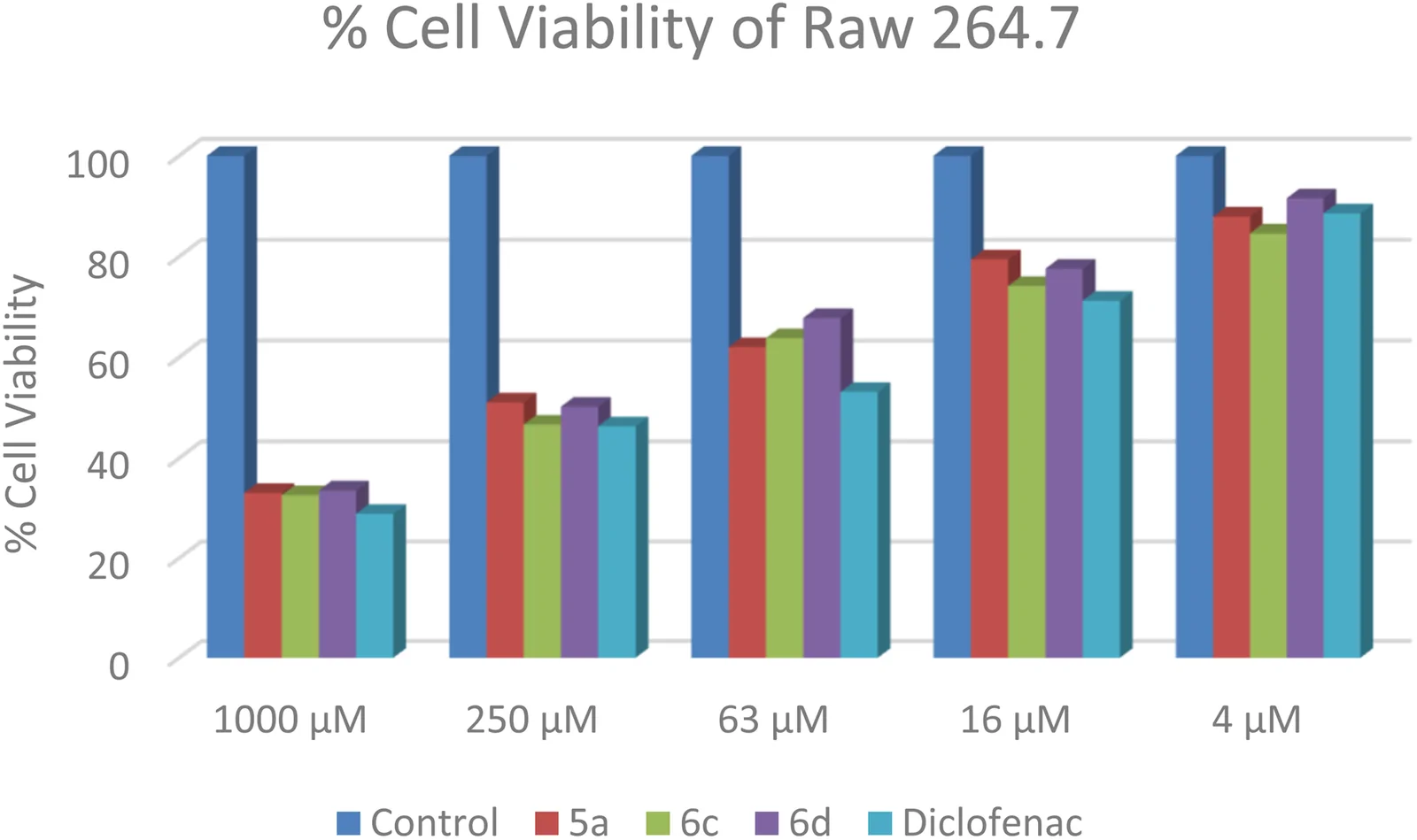
After pre-treating LPS-stimulated RAW264.7 macrophages with the tested compounds (5a, 6c, and 6d) and the positive control, diclofenac sodium, for 24 h, cellular viability was assessed using the MTT assay. Values are mean ± SD (*n* = 3).

This study shows target engagement and key downstream responses (*in vitro* and *in vivo*), but a limitation is that specific intracellular signaling pathways were not fully mapped. Future research with advanced cellular models will help clarify these molecular mechanisms.

### 3.3. *In silico* study

#### 3.3.1. Molecular modeling

Compounds 5a–g and 6a–g were subjected to molecular docking simulations within the active site of COX-2 in complex with SC-558 (PDB ID : 1CX2).^37^ The full dataset is listed in SI Table S1. Fig. 8 illustrates the binding modes of the most active triazolopyrimidines, 5a, 6c, and 6d. Additionally, molecular docking of these compounds into the 5-LOX-NDGA complex (PDB ID: 6n2w)^38^ was performed to evaluate binding interactions (Fig. 9).

**Fig. 8:**
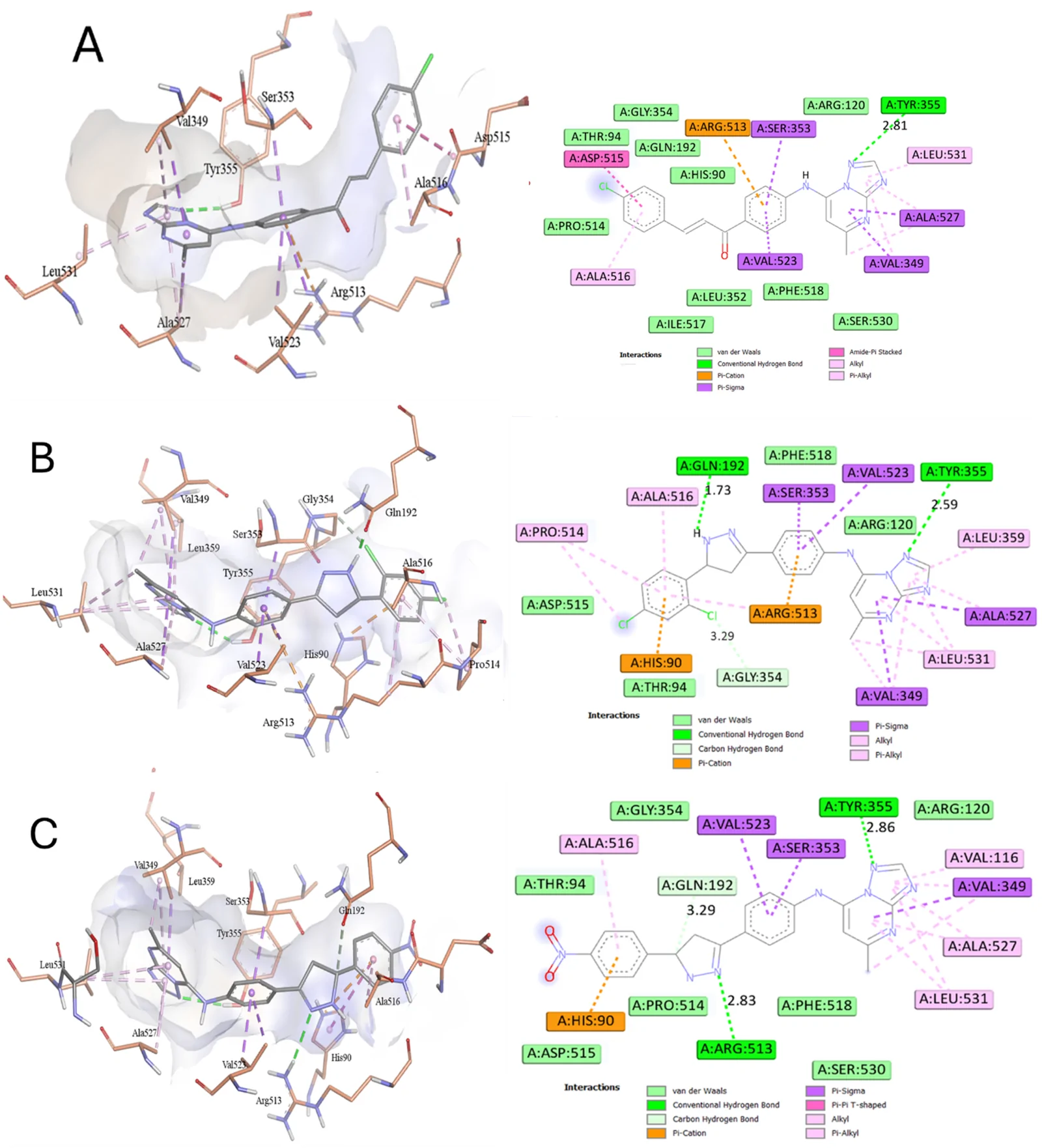
Evaluation of the COX-2 active site binding affinities for compounds (A) 5a, (B) 6c, and (C) 6d.

**Fig. 9:**
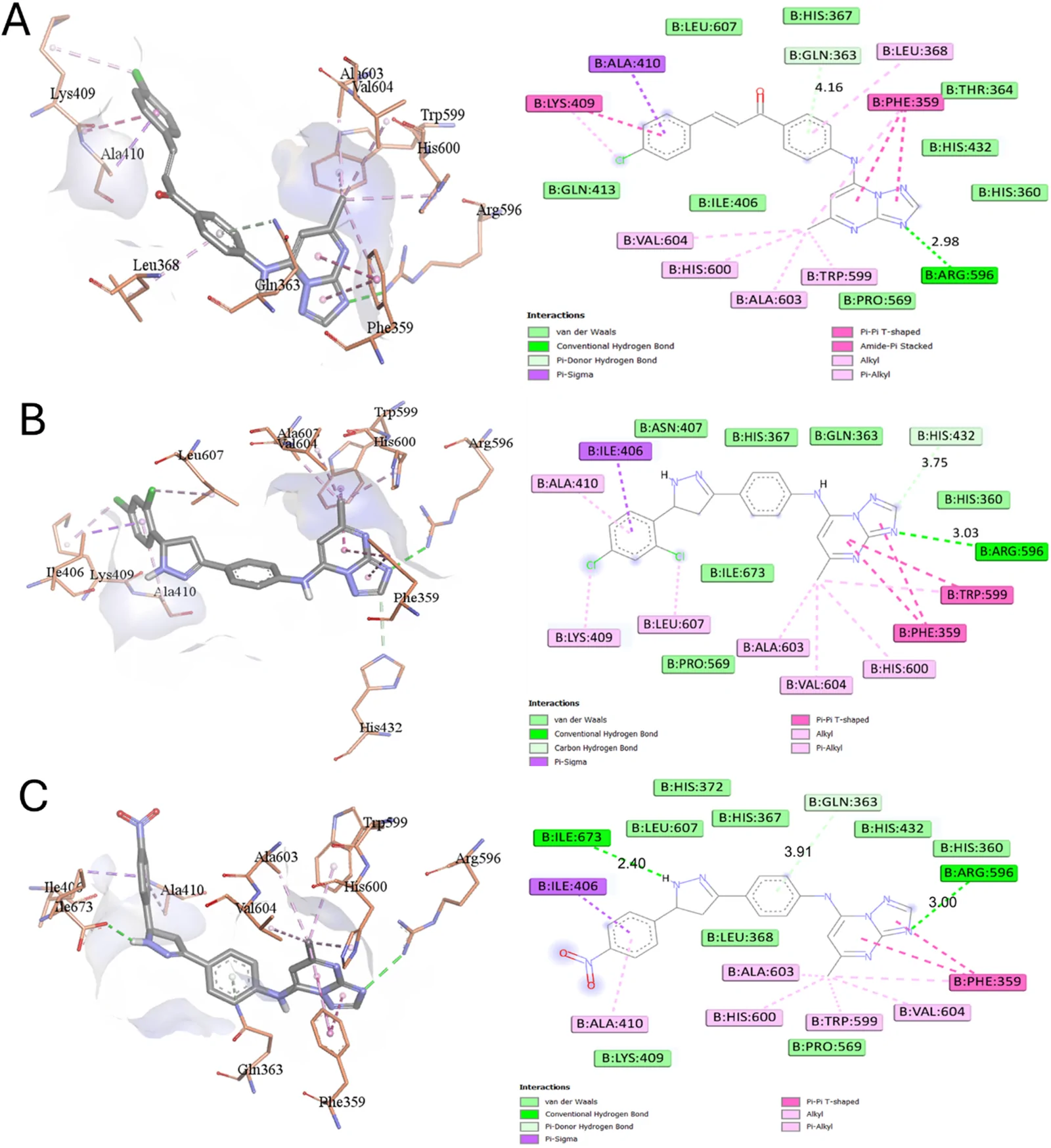
Evaluation of the 5-LOX active site binding affinities for compounds (A) 5a, (B) 6c, and (C) 6d.

##### 3.3.1.1. Docking into the COX-2

The key distinction between the COX-1 and COX-2 binding domains lies in the bigger overall dimensions of COX-2. This increased size, driven by an extra side pocket, acts as a critical binding region for many selective COX-2 inhibitors. Mutating residues Ile-434, His-513, and Ile-523 in COX-1 to the sterically smaller Val-434, Arg-513, and Val-523 in COX-2 forms this distinct "selectivity" pocket. The side pocket of COX-2 enzyme is composed of amino acids Ala516, Arg513, Gln192, His90, Ile517, Leu352, Phe518, Ser353, and Val523.^57^

The compounds 5a–g and 6a–g had docking scores varying from −9.3 to −6.2 Kcal mol^−1^. All examined compounds were able to reach the additional side pocket and form interactions – except 5d – with Arg513 *via* the central phenyl ring or 4,5-dihydropyrazolyl ring as in 6d and 6f (Table S1). Additionally, all compounds – except 6a – successfully established hydrophobic interactions with Ser353 (a pivotal amino acid determining the binding topology of the SC-558 inhibitor) and Val523 through their central phenyl ring. The triazolo[1,5-*a*]pyrimidine ring participated in hydrophobic interactions with Leu531, Ala527, Val349, and Leu359 in nearly all compounds. Compounds 5a–g, 6a, 6c, and 6d formed hydrogen bonds with Tyr355 *via* the nitrogen of the triazole ring (SI Table S1).

The most effective compounds, 5a, 6c, and 6d, exhibited these general interactions as illustrated in Fig. 8. The 4-chlorophenyl ring of compound 5a engaged in a pi–amide interaction with Asp515 and a hydrophobic interaction with Ala516. On the other hand, compounds 6c and 6d participated in pi–cation interactions with His90 and hydrophobic interactions with Ala516. Additionally, compound 6c demonstrated a hydrophobic interaction between the dichlorophenyl ring and Pro514 and Arg513, along with a hydrogen bond between the NH of the 4,5-dihydropyrazolyl ring and Gln192.

Notably, compound 6d, which exhibited the highest COX-2 inhibition, achieved an impressive docking score of −8.2 and was capable of interacting with 11 amino acids that define the COX-2 binding cavity.

It was notable that *para*-chloro-substituted chalcone 5a had a better docking score (−9.3 Kcal mol^−1^) than the *ortho*-chlorinated analog 5b (−7.4 Kcal mol^−1^). This higher affinity indicated a stronger binding with the target COX-2 and rationalized the obtained *in vitro* results. In the pyrazole series, mono-chlorinated derivatives 6a and 6b had similar docking scores (−6.4 Kcal mol^−1^ and −6.3 Kcal mol^−1^, respectively) and an equal number of hydrophobic interactions at the COX-2 active site. However, derivative 6a, with a *para*-chlorophenyl group, formed two H-bonds compared to one in 6b, leading to better binding and explaining the superior COX-2 potency and selectivity of 6a.

It was remarkable that the mono-halogenated phenyl derivatives in the chalcone series (5a, b, e) had better docking scores (−9.3, −7.4, and −7.2 Kcal mol^−1^, respectively) compared to their analogues in the pyrazole series (6a, b, e) with docking scores of −6.4, −6.3, and −6.6 Kcal mol^−1^, respectively. These docking results were consistent with those obtained from the *in vitro* assay. The di-chlorinated derivatives 5c and 6c had comparable docking scores (−8.7 and −7.2 Kcal mol^−1^, respectively). However, the more potent and selective COX-2 inhibitor, pyrazole 6c, effectively interacted with the COX-2 active site *via* three H-bonds with Tyr355, Gly354, and Gln192 (one of the key amino acids in the selectivity pocket) compared to one in 5c with Tyr355.

Although the most potent compounds established all the essential interactions similar to other derivatives, the specific substituents—such as chlorine atoms in 5a and 6c and the nitro group in 6d—exert electronic effects that enhance favorable interactions with active-site residues, thereby conferring superior activity compared to the other compounds.

##### 3.3.1.2. Docking into the 5-LOX

5-LOX is an iron-containing dioxygenase that contributes significantly to the biosynthesis of leukotrienes from arachidonic acid. Unlike the ref. 5-LOX inhibitor zileuton, NDGA, the inhibitor housed in the co-crystal structure, does not bind the active-site iron; instead, it is oriented such that one of its catechol rings lies directly over the metal center, thus preventing access to the open coordination site on the iron ion.^38,58^

NDGA exhibited a binding affinity of −7.1 kcal mol^−1^, stabilized by two pi–pi stacking interactions between the ligand's phenyl rings and residues His372 and Phe359. Additionally, the complex was anchored by a hydrogen bond with His600 and multiple hydrophobic contacts involving Leu607, Ala410, and Trp599.

The compounds tested, 5a, 6c, and 6d, achieved docking scores of −7.8, −7.9, and −8.0 kcal mol^−1^, respectively (Table S2). Interestingly, the tested 5-LOX inhibitors did not interact with the iron cofactor; however, they obstructed the pathway to access iron, thereby inhibiting its activity, like NDGA. Remarkably, all three compounds were able to form pi–pi stacking with Phe359 through the triazolo[1,5-*a*]pyrimidine ring (Fig. 9). Additionally, the triazolo[1,5-*a*]pyrimidine ring participated in hydrogen bonding with Arg596 and engaged in hydrophobic interactions with Val604, Ala603, His600, and Trp599. In addition.

Compound 5a, *via* its chlorophenyl ring, formed pi-sigma and pi-amide interactions with Ala410 and Lys409, respectively. Furthermore, the central phenyl rings of 5a and 6d made a carbon–hydrogen bond with Gln363. Moreover, compound 6c, characterized by its dichlorophenyl ring, was involved in a pi-sigma interaction with Ile406 and participated in hydrophobic interactions with Leu607, Lys409, and Ala410.

The most potent 5-LOX inhibitor 6d had a docking score of −8.0 kcal mol^−1^ and formed a hydrogen bond *via* its 4,5-dihydropyrazolyl (NH) group with Ile673 in addition to a pi-sigma interaction with Ile406.

#### 3.3.2. Molecular dynamics simulation

The structural stability of the enzyme–ligand complex was evaluated by applying molecular dynamics (MD) simulations on the optimal position of the most potent inhibitor 6d inside the COX-2 active site. Thus, a 100 ns MD simulation at a physiological temperature of 310.15 K was used to evaluate and compare the COX-2/6d complex system to the unbound COX-2. First, the convergence of the complex system was verified using the simulation's constant pressure, constant potential energy, and consistent temperatures. The differences between the target COX-2 with and without compound 6d were examined by monitoring the drift of the root mean square deviation (RMSD) across the simulation runs. As seen in Fig. 10, the RMSD values for the complex and the unbound COX-2 displayed a similar pattern, indicating that the target COX-2 had stabilized. Furthermore, the overall compactness and solvent exposure were very similar to those of the unbound COX-2, indicating that no significant conformational changes occurred, and the complex with compound 6d is stable. Additionally, the average distance between 6d and the COX-2 remained constant during the MD simulation. The stability of the COX-2/6d complex was further confirmed by the lower variance in the complex's RMSF when compared to the free COX-2 (Fig. 10).

**Fig. 10:**
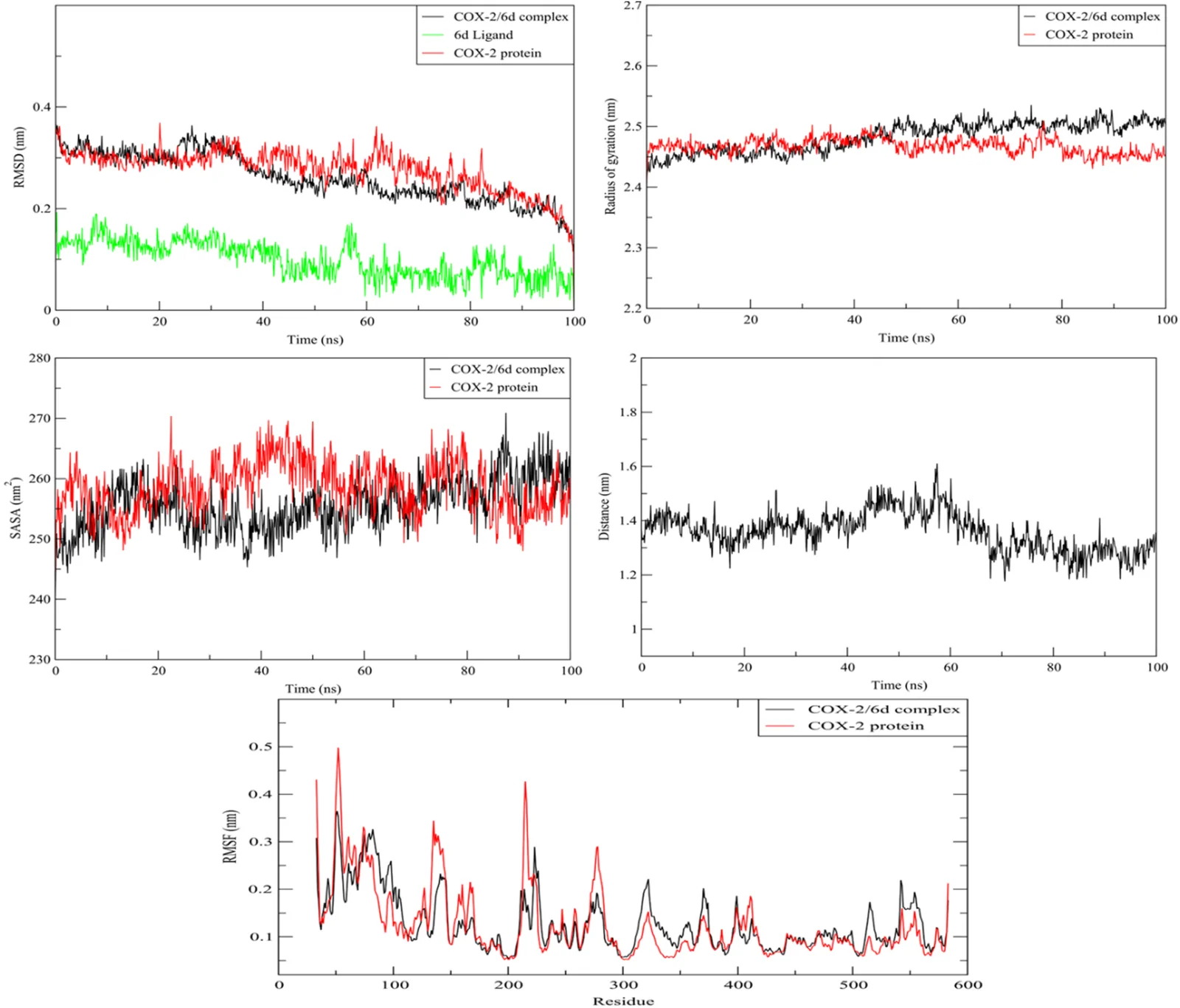
Plots of MD analysis for COX-2/6d complex over 100 ns, including root mean square deviation (RMSD), ligand-receptor distance, solvent-accessible surface area (SASA), Radius of gyration (*R*_g_), and Root Mean Square Fluctuation (RMSF).

The stability of the important interactions identified in the molecular docking study was validated by consistent binding interactions, including Pi-cation interactions with His90, hydrogen bonds with Tyr355 and Arg513, and hydrophobic interactions with the target protein throughout the MD trajectories (Fig. 11).

**Fig. 11:**
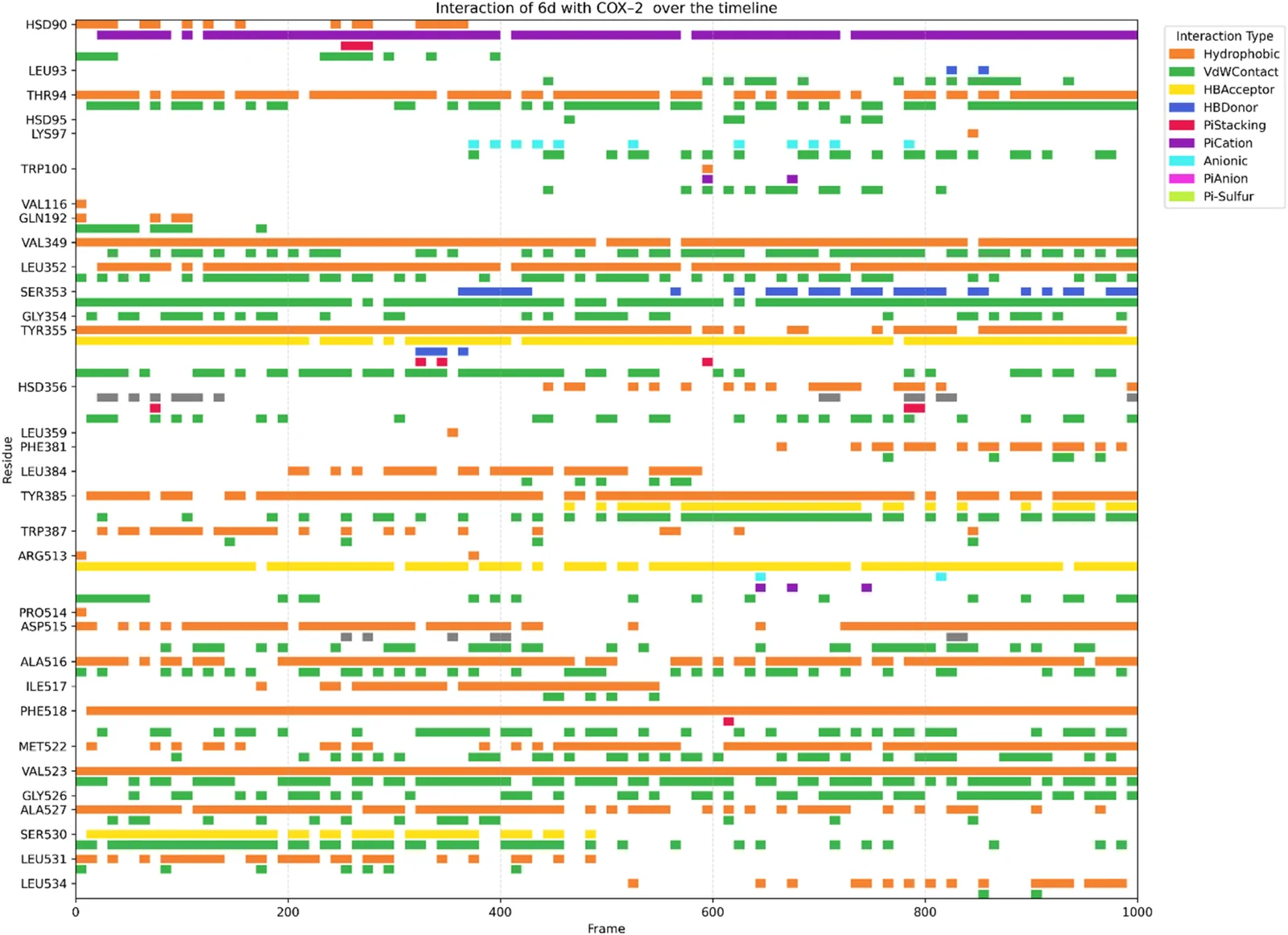
MD analysis plots for the COX-2/6d complex over 100 ns, showing different interaction types involving the involved amino acids.

#### 3.3.3. Drug-likeness, physicochemical properties

The most potent anti-inflammatory compounds 5a, 6c, and 6d were selected for further bioinformatic prediction using the SWISSADME web tool developed by the Swiss Institute of Bioinformatics.^59,60^ Furthermore, the results obtained were compared with those of celecoxib and diclofenac sodium (Table S3).

The results revealed that all examined compounds obey both Lipinski's rule of five (Mwt < 500, Mlog *P* < 5, number of hydrogen bond donors < 5, and number of hydrogen bond acceptors < 10) and Veber's rule (number of rotatable bonds < 10, and Total Polar Surface Area < 140 Å^2^) without any violations. The aqueous solubility of the most active inhibitors was evaluated, and the identified candidates were predicted to exhibit Log S values ranging from −4.85 to −5.98, indicating moderate to low-moderate solubility, which is consistent with the requirements for oral bioavailability in drug-like molecules.

According to the oral bioavailability spider plots (Fig. 12), the tested derivatives, celecoxib and diclofenac sodium, were expected to exhibit a single violation associated with the fraction of sp^3^-hybridized carbons.

**Fig. 12:**
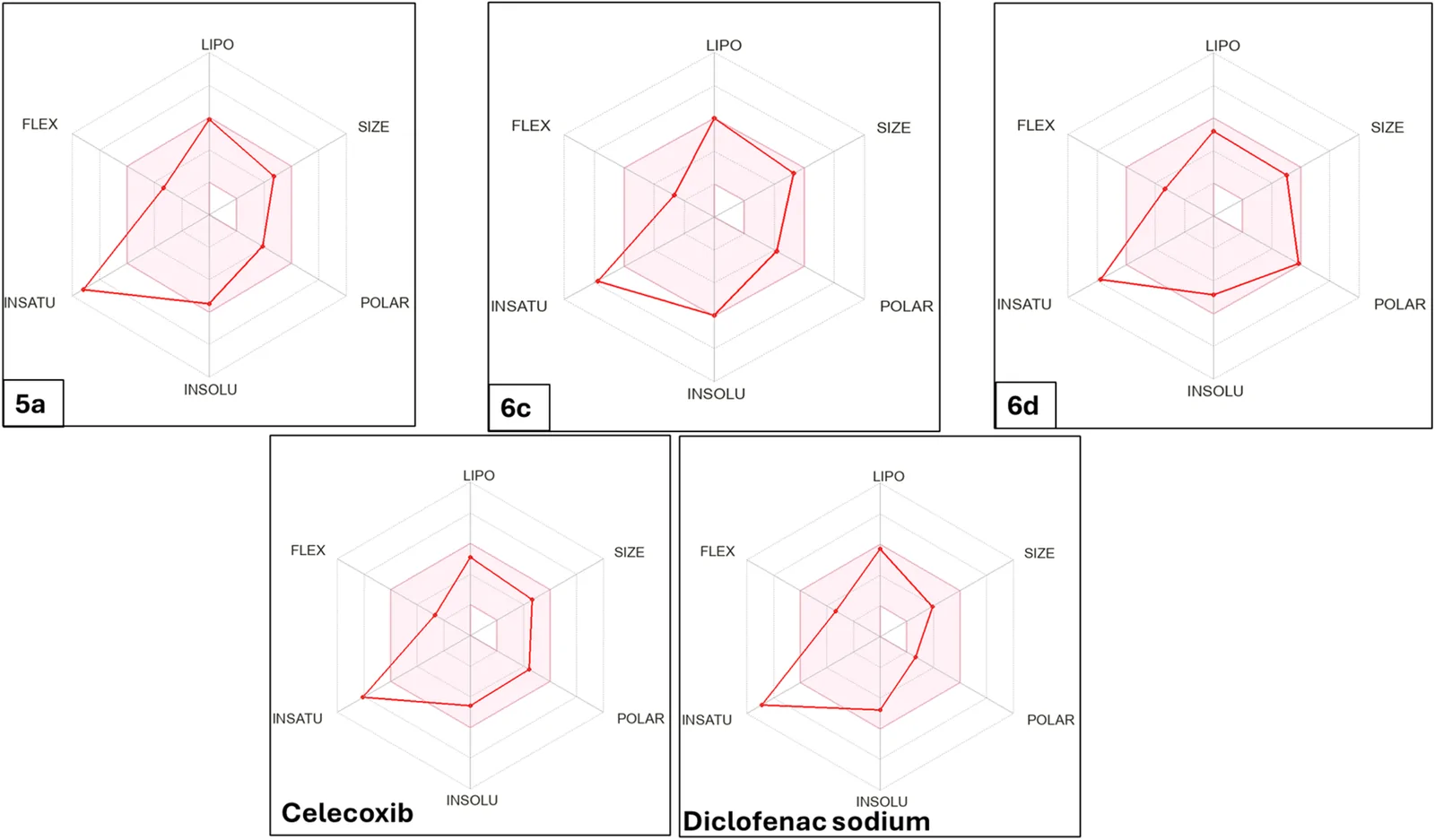
Oral bioavailability spider plots for the tested derivatives (5a, 6c, and 6d) and positive control drugs.

Interestingly, given their structural features, the evaluated derivatives were not anticipated to exhibit any PAIN alerts. The pharmacokinetics prediction showed that the newly synthesized derivatives, along with the reference drugs, were expected to have high gastrointestinal absorption. Additionally, 6c and 6d were likely to have no BBB permeability, while 5a and 6c were probably not affecting P-glycoprotein-mediated drug efflux. The BOILED egg model prediction (Fig. 13) illustrated favorable pharmacokinetics, with all the promising derivatives located within the white zone. This suggests energy-independent movement of molecules from the gut lumen into the bloodstream, similar to reference celecoxib.

**Fig. 13:**
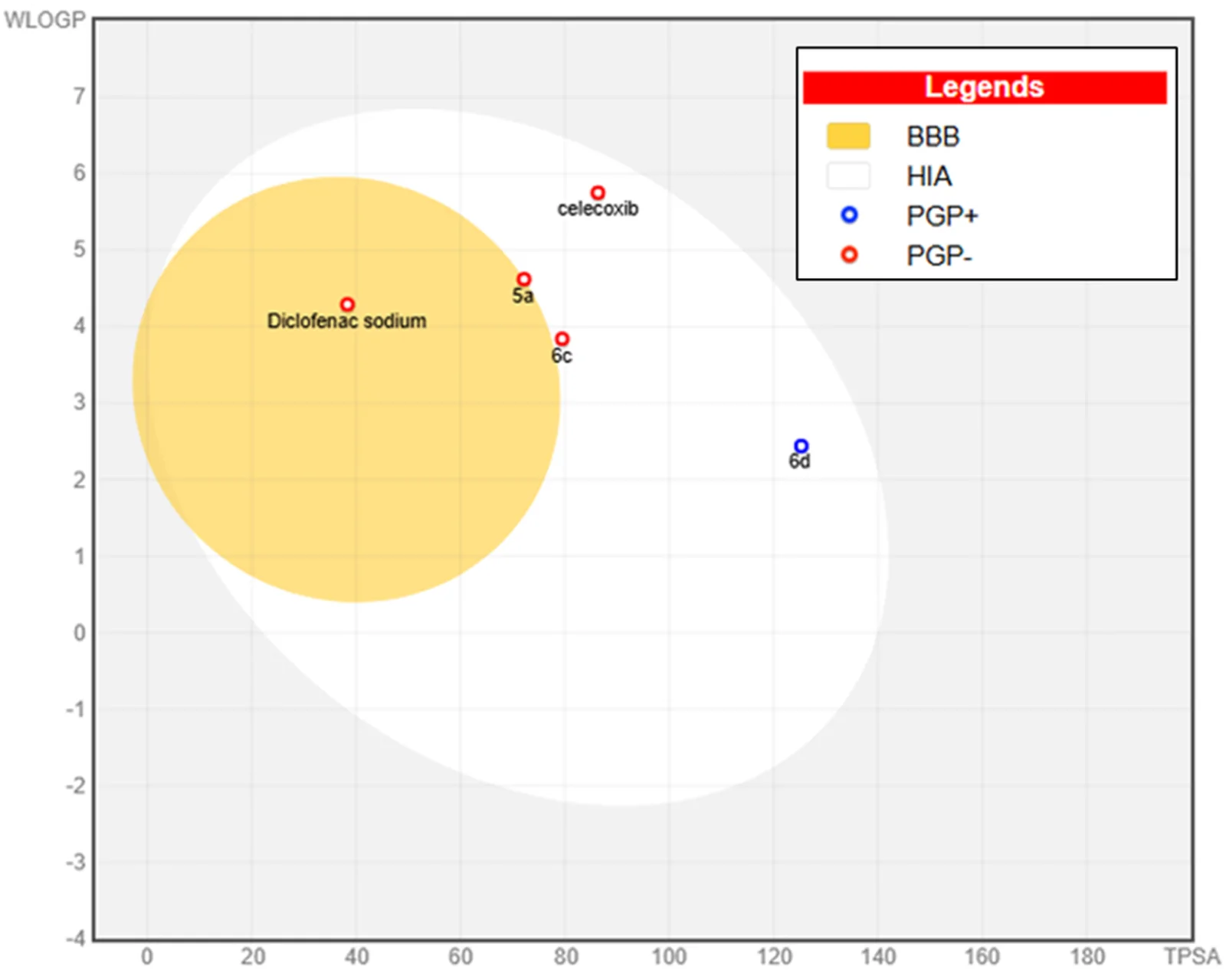
BOILED-Egg Prediction for 5a, 6c, 6d, and reference drugs.

#### 3.3.4. *In silico* toxicology

The safety profile of the most active derivatives was predicted using the Protox-3 web tool, which was created and is sustained by the Structural Bioinformatics Group at Charité – University Medicine Berlin, Germany^61^ compared to the references, celecoxib and diclofenac sodium. The expected data were presented in Table S4. In comparison to celecoxib (LD_50_ = 1400 mg kg^−1^), compounds 5a, 6c, and 6d were expected to have lower LD_50_ (300, 1000, and 800 mg kg^−1^, respectively). On the other hand, these results were much higher than those anticipated for diclofenac (56 mg kg^−1^).

These expected LD_50_ values suggest a low incidence of acute toxicity in animal models. The results implied that all the newly synthesized derivatives would be inactive for nephrotoxicity, cardiotoxicity, cytotoxicity, and nutritional toxicity. Additionally, they were predicted to display inactivity against the estrogen receptor alpha, the androgen receptor, and aromatase. Further toxicological evaluations were carried out using the ADMETlab 3.0 platform (Table 6).^62^ The results anticipated that 5a, 6c, as well as celecoxib, had a moderate probability of causing genetic mutations.

**Table 6:** ADMETlab 3.0 prediction of safety and excretion profiles for the most potent derivatives and reference drugs

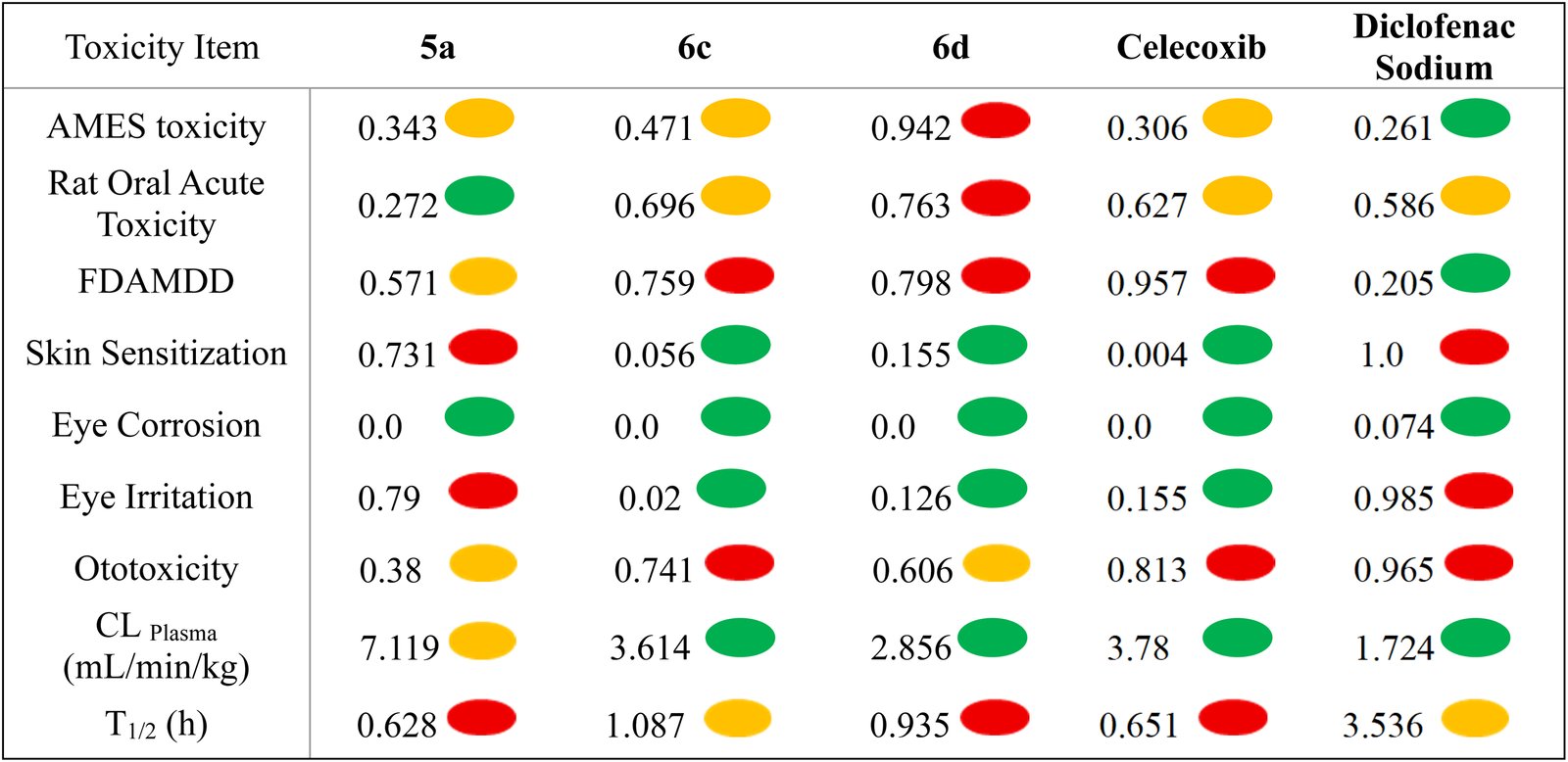

The pyrazole derivatives 6c and 6d were expected to have an encouraging risk-benefit profile regarding skin sensitization and eye irritation. Remarkably, all tested derivatives were expected not to be eye corrosive (*P* = 0.00). Compounds 6c and 6d suggested slow elimination properties (3.614 and 2.856 mLmin^−1^kg, respectively), similar to those of celecoxib and diclofenac sodium (3.78 and 1.724 mLmin^−1^kg, respectively). On the other hand, 5a, 6d, and celecoxib were anticipated to have extremely short half-lives (*T*_1/2_) with values of 0.628, 0.935, and 0.651 h, respectively. While 6c and diclofenac sodium were suggested to display short half-lives (*T*_1/2_) (1.087 and 3.536 h, respectively).

## 4. Conclusion

In summary, two series of [1,2,4]triazolo[1,5-*a*]pyrimidine-based hybrids 5a–g and 6a–g were synthesized, characterized, and assessed for their inhibitory activity against COX-1 and COX-2. Among the compounds tested, 5a, 5e, and 6c, 6d, and 6f exhibited remarkable efficacy, particularly 6d (IC_50_ COX-2 = 1.237 µM), which had four times the activity of diclofenac sodium and was comparable to celecoxib. Additionally, the most promising candidates, 5a, 6c, and 6d, were screened for their *in vivo* anti-inflammatory efficacy and demonstrated a time-dependent anti-inflammatory potential. These compounds were also tested for their activity against the 5-LOX enzyme and the pro-inflammatory cytokines IL-6 and TNF-α, highlighting the notable efficacy of compound 6d (IC_50_ 5-LOX = 1.031 µM and 90.9% inhibition of IL-6). Additionally, the antioxidant activity against ROS generation showed outstanding scavenging potential for 5a and 6d (IC_50_ = 64.02 µM and 34.45 µM, respectively), exceeding those of celecoxib and diclofenac sodium (IC_50_ = 122.19 µM and 89.59 µM, respectively). The molecular docking simulation achieved a comprehensive elucidation of the molecular binding modes and affinities for COX-2 and 5-LOX. Ultimately, internet-based platforms offered computational predictions for essential pharmacokinetic parameters.

## 5. Ethical statement

All animal procedures comply with the guidelines for the use of laboratory animals of Zagazig University and were approved by the Research Ethics Committee, Zagazig University, Egypt (Approval number: ZU-IACUC/3/F/316/2026).

## Author contributions

CRediT: Esraa Elazony: methodology, validation, writing – original draft, formal analysis, data curation, resources. Nermine A. Osman: conceptualization, writing – review & editing, supervision, resources. Moataz A. Shaldam: methodology, software, writing – review & editing. Hanan A. Abdel-Fattah: conceptualization, writing – review & editing, supervision. Amany M. Ghanim: validation, writing – original draft, formal analysis, data curation, writing – review & editing, supervision, resources, software, conceptualization.

## Conflicts of interest

The authors report no conflicts of interest.

## Supplementary Material

RA-016-D6RA04602A-s001

## Data Availability

The authors confirm that the data supporting the findings of this study are available within the article [and/or] its supplementary information (SI). Supplementary information is available. See DOI: https://doi.org/10.1039/d6ra04602a.
