# Supplementary material for: Tailored [1,2,4]triazolo[1,5-a]pyrimidine hybrids as promising anti-inflammatory agents with COX/5-LOX/ROS/IL-6 multifunctional inhibitory activity: design, synthesis, SAR, and in silico study
Source: RSC Adv. 2026 Sep 9;16(43):53756–75. doi: 10.1039/d6ra04602a (PMC13557024; doi:10.1039/d6ra04602a)
Supplement: Supplementary file 1 [file RA-016-D6RA04602A-s001.pdf]

Supplementary Information (SI) for RSC Advances.
This journal is © The Royal Society of Chemistry 2026

Tailored [1,2,4]Triazolo[1,5-a]Pyrimidine Hybrids as Promising Anti-inflammatory Agents with COX/5-LOX/ROS/IL-6 Multifunctional Inhibitory Activity: Design, Synthesis, SAR, and In-Silico Study

Esraa Elazony[a], Nermine A. Osman[a], Moataz A. Shaldam[b,c], Hanan A. Abdel-Fattah[a], Amany M. Ghanim[a,*].

[a]*Department of Pharmaceutical Organic Chemistry, Faculty of Pharmacy, Zagazig University, Zagazig 44519, Egypt.*
[b]*Department of Pharmaceutical Chemistry, Faculty of Pharmacy, Kafrelsheikh University, P.O. Box 33516, Kafrelsheikh, Egypt.*
[c]*Department of Pharmaceutical Chemistry, Faculty of Pharmacy, Alsalam University, Kafr Alzayat, Egypt*

Corresponding author: Amany M. Ghanim, email: amanyghanim@yahoo.com, AMMettwalli@pharmacy.zu.edu.eg
ORCID: https://orcid.org/0000-0002-3791-5154

Fig. S1. The ABX system of the pyrazoline ring in compound **6c**.

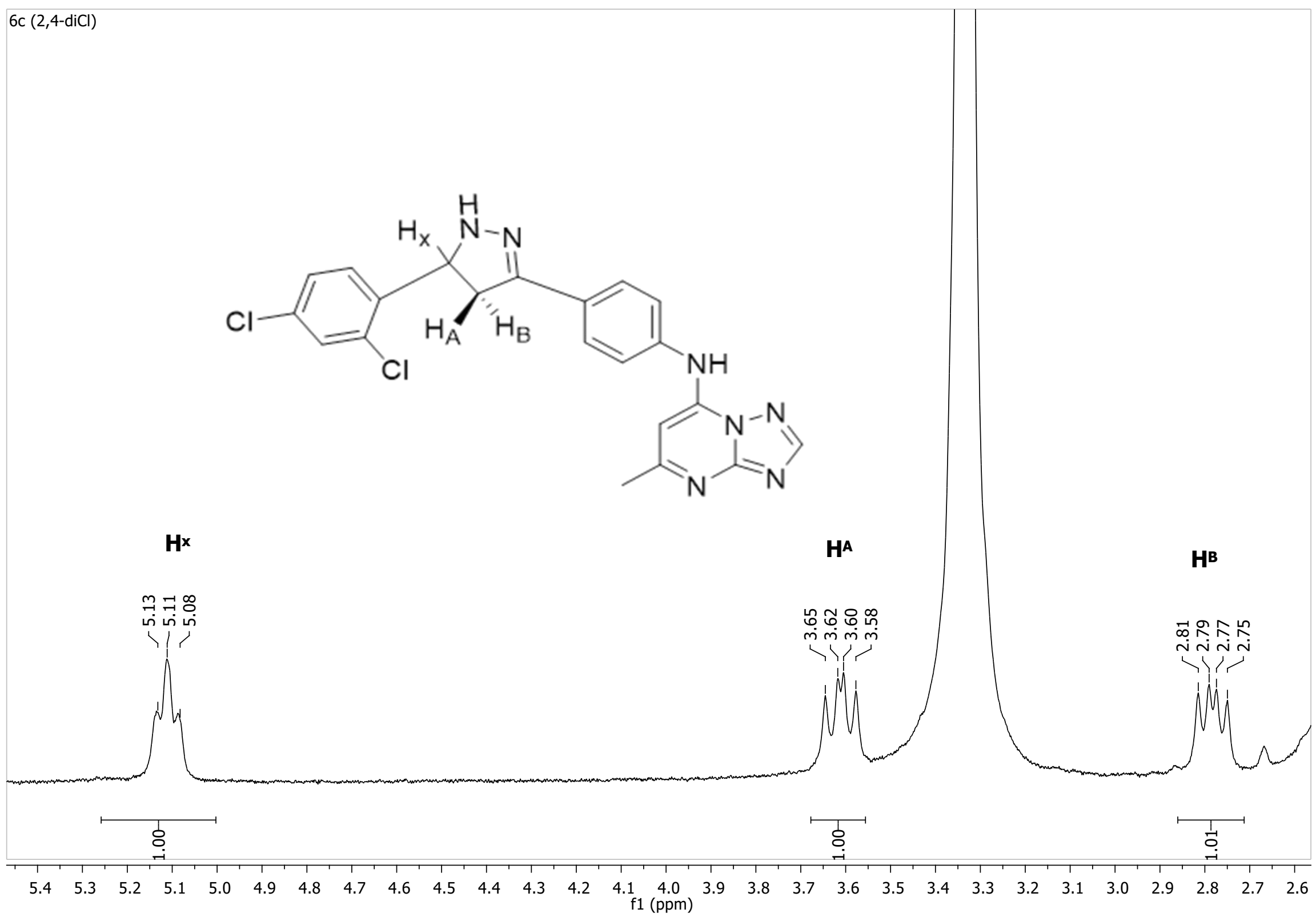

Biological activity

In vitro inhibition assay of COX-1 and COX-2

The test samples were dissolved in DMSO, and the desired test concentration was diluted to 10X with COX assay buffer before use. A volume of 10 µl of diluted test inhibitor or assay buffer was added to the assigned wells as a sample screen or enzyme control, respectively. For the inhibitor control, 2 µl of the provided COX inhibitor and 8 µl of COX assay buffer were added to one of the wells. For reaction preparation, the COX cofactor was diluted 200 times just before use by adding 2 µl of COX cofactor to 398 µl of COX assay buffer, then mixing well. The solution of arachidonic acid (AA) was prepared through the addition of 5 µl of the supplied AA to 5 µl of NaOH, then diluted 10 times by adding 90 µl ddH_2O. In each well, 80µl of the master mixture was added (containing 76 µl COX assay buffer, 1µl COX probe, 2 µl diluted COX cofactor, and 1 µl COX). Then, 10 µl of diluted AA/NaOH solution was added using a multi-channel pipette to initiate the reactions at the same time.

Plate Setup: a serial dilution (100 µM, 10 µM, 1 µM, 0.1 µM, and 0.01 µM) of each sample. Each concentration is allocated to its own independent well on the plate.

The fluorescence (Ex/Em = 535/587 nm) was measured at 25°C for 10 min. [1,2]

The COX inhibitory activity of SC560 and Celecoxib, standard inhibitors of COX-1 and COX-2, respectively, was also determined. The change in fluorescence between two points, t_1 and t_2, was determined, and relative inhibition was calculated as follows:

%Inhibition = (change of N - change of S)/change of N x 100.

All bioactivity data processing was performed using GraphPad Prism 8.0 (La Jolla, CA, USA). The sigmoidal dose−response model was obtained using the nonlinear regression model log(inhibitor) vs variable slope to determine the IC_{50} Michaelis constant (K_M) values of the compounds, and maximal velocity (V_{MAX}) of ACE were determined by Lineweaver−Burk plots using the Pharmacological and biochemistry transform and simple linear regression functions of GraphPad Prism 8.0. Statistical evaluation was done by one-way ANOVA using Dunnett's multiple comparison test with the significance level set at 0.05.

In vivo anti-inflammatory activity

The acute carrageenan-induced paw oedema standard method in rats was utilized to evaluate the anti-inflammatory activity of the tested compounds *in vivo.*[3] Adult Wister rats of either sex (pregnant female animals were excluded from the study), weighing 120–150 g, were organized into five groups, with each group consisting of six animals. DMSO was used to dissolve the tested compounds at a dose of 10 mg kg^{-1} (rat body weight), diclofenac sodium mol equivalent, were given intraperitoneally 1 h before the induction of inflammation. The control group was treated with DMSO only. Paw edema was induced by subcutaneous injection of a freshly prepared 1% solution of carrageenan in saline (0.9%, 0.1 ml per rat) into the right hind paw subplantar tissue of rats. The thickness of the paw was measured (in mm) after successive time intervals (2, 3, 4, and 24 h) and compared with the original hind paw thickness of each rat for determining the edema thickness. Data were assembled, assessed, revised, and analysed (SPSS 16). Quantitative variables from a normal distribution were presented as means ± SE (standard error). The anti-inflammatory response was expressed as percentage inhibition of edema thickness in treated animals compared to the control group according to Eqn. (1).

$$\%\ Inhibition\ of\ edema = \frac{Vc - Vt}{Vc} \times 100 \quad \ldots\ldots\ldots\ldots\ldots\ldots\ldots (1)$$

Where V_c and V_t are the means of oedema paw thickness for the control and tested compound-treated animal groups, respectively.

The potency of the tested compounds was expressed as % inhibition of edema thickness for the tested compounds relative to % inhibition of edema for diclofenac sodium "reference standard" at 2 h, 3 h, 4 h, and 24 h effects "diclofenac sodium reveals its maximum bio-properties at the mentioned time" according to eqn. (2).

$$\% \text{ Potency} = \frac{\% \, inhibition \, of \, edema \, for \, the \, tested \, compound \, treated \, group}{\% \, inhibition \, of \, edema \, for \, indomethacin \, treated \, group} \text{ x } 100 \ldots \quad (2)$$

In vitro inhibition assay of 5-LOX

The test samples were dissolved in DMSO at the desired concentration, where the volume does not exceed 2% of the final reaction volume per well. For solvent control, 2 µl of DMSO at its final concentration was added to the test wells. For inhibitor control, 2 µl of zileuton was used. Then, the volume was brought up to 40 µl in each well by adding 38 µl of LOX assay buffer. For enzyme control, 40 µl of LOX assay buffer was added to a well. Then, 40 µl of the reaction mix (containing 34 µl LOX assay buffer, 2 µl LOX probe, and 4 µl 5-LOX enzyme) was added to the wells and incubated at room temperature for 10 minutes before adding the LOX substrate. The LOX substrate was diluted (12500 X) in LOX assay buffer by using a 1:25 dilution factor to get a 500 X solution, then re-diluted in the LOX assay buffer at 1:100 to obtain a 5X solution. Finally, a volume of 20 µl of 5X solution will be needed per reaction. After the addition of the substrate, the fluorescence (Ex/Em = 500/536 nm) was measured at the second minute with 30 s intervals for 30-60 min. [1]

The plate setup and subsequent calculation of percentage inhibition and IC_{50} values were carried out exactly as described for the COX assay.

ROS scavenging assay

A volume of 0.100 mL of the tested compound at different concentrations (0.010 to 0.100 mg/mL) was completely mixed with 0.880 mL of tris-HCl buffer (0.05 M, pH 7.4) and 0.02 mL of pyrogallol (60 mM). The absorbance (A) was recorded at 325nm every 30 seconds for 3 minutes. The change in absorbance (ΔA) in 3 minutes was utilized to calculate the percentage of scavenging activity.[4,5]

Inhibition of IL-6 production

RAW 264.7 (ATCC TIB-71) cells used in this study were macrophage cell lines derived from a mouse tumor purchased from the American Type Culture Collection (ATCC) in the United States. The cell culture supernatants were incubated in a 96-well plate coated with IL-6 antibodies for 2.5 hours at room temperature. After washing, the prepared biotin anti-mouse IL-6 antibodies were added. Next, the unbound antibody was washed away, and HRP-conjugated streptavidin was added, followed by another wash. A TMB substrate solution was added, and the color developed. Then the stop solution was added, and the amount of IL-6 was measured by measuring absorbance at 450 nm. The level of the inflammatory mediator IL-6 was measured in LPS-induced RAW 264.7 macrophage cells in the absence and presence of the selected compounds and the reference drug.[6]

The inhibition percentage was calculated from the following equation

$$\% \text{ inhibition} = \frac{[IL-6]LPS - [IL-6]inhibitor + LPS}{[IL-6]LPS - [IL-6]Untreated} \times 100$$

Inhibition of TNF-α production

RAW 264.7 murine macrophages (ATCC TIB-71) were purchased from the ATCC in the United States. Briefly, 50 µL of standards, diluted samples, or controls were added to the designated wells, followed by 50 µL of the antibody cocktail. The plate was incubated for 1 h at room temperature with shaking (400 rpm), washed three times with 1× Wash Buffer, and incubated with 100 µL of TMB development solution for 10 min in the dark at room temperature. The reaction was terminated by adding 100 µL of stop solution, and the absorbance was measured at 450 nm using a microplate reader. TNF-α concentrations were calculated from the standard curve and expressed as pg/mL.[7,8]

The percentage of inhibition was calculated according to the following equation

$$\% \text{ TNF-}\alpha \text{ inhibition} = \frac{[TNF-\alpha]LPS - [TNF-\alpha]inhibitor + LPS}{[TNF-\alpha]LPS - [TNF-\alpha]Untreated} \times 100$$

Cell viability assay (MTT assay)

3-(4,5-Dimethylthiazol-2-yl)-2,5-diphenyltetrazolium bromide (MTT) was obtained from Sigma-Aldrich (USA). MTT was dissolved in phosphate-buffered saline (PBS) (5 mg/ml) and used according to Mosmann.[9] A total of 100 µL of Raw264.7 cells were seeded at a density of 1 × 10^6 cells per well in 96-well plates and incubated for 24 hours. After this incubation period, the cells were treated with various concentrations of the test compounds for an additional 24 hours. Following treatment, 10 µL of freshly prepared MTT solution was added to each well, and the plates were incubated again for 4 hours at 37 °C. After incubation, the formazan crystals formed were dissolved in 300 µL of DMSO, and then 100 µL of this solution was transferred back to a 96-well plate. The absorbance was measured using a multi-well plate reader (ROBONIK P2000 Eia reader). The cytotoxicity of the test compounds was assessed by comparing the results to the control group treated with the vehicle alone, and the data were expressed as a percentage relative to the negative control. All assays were conducted in triplicate and repeated three times for reliability.[9]

Molecular modeling

The RCSB PDB supplied the 3D structures of two target enzymes, COX-2 protein (1CX2 [10]) and the 5-LOX protein (6n2w [11]) in PDB format. Water and other non-protein components were eliminated using AutoDockTools, and hydrogen atoms were incorporated.[12] The 2D and 3D geometric representations of the ligand compounds were created using Marvin Sketch.[13] In this docking study, AutoDock Vina was used to forecast protein-ligand interactions and binding affinities using an exhaustiveness parameter of 18, and generating 10 binding poses per ligand.[14] The grid box, measuring 20.9Å x 16.2Å x 19.2Å, was established by the center of the co-crystallized ligand (x, y, z; 23.9, 21.6, 15.4) for COX-2, while the grid box, measuring 14.5Å x 16.0Å x 24.0Å, was defined by the center of the co-crystallized ligand (x, y, z; 35.9, 65.3, 38.3) for 5-LOX. For each ligand, the docking pose with the lowest binding energy was selected for subsequent interaction analysis. Discovery Studio Visualizer was utilized to examine and present the docking results.[15] To confirm the accuracy of our docking protocol settings, the co-crystallized ligand SC-558 was redocked into the COX-2 active site, and the natural inhibitor NDGA was redocked into the 5-LOX active site, successfully reproducing their original conformations (RMSD: 0.56 and 1.97 with scores of -11.0 and -7.1, respectively).

Molecular dynamics simulation

CHARMM-GUI solution builder [16,17] was used to create the input files for MD calculations using the CHARMM force field parameters for COX-2. CHARMM General Force Field (CGenFF) was used to generate topologies of the chosen ligands using the CGenFF server. There are five steps in the CHARMM-GUI solution builder. The tool reads the coordinates of the protein-ligand combination in the first stage. In the second phase, the protein-ligand combination is solvated, and the system's size and structure are ascertained. To neutralize the system, Na^+ and Cl^- ions are introduced in this phase. The third phase establishes Periodic Boundary Conditions (PBCs), which approximate a large system using a unit cell that is repeated in all directions. Only the atoms within the PBC box are included in the simulation. Complexes with dimensions of 77 Å, 77 Å, and 77 Å for the PBC box in x, y, and z, respectively, have been neutralized by adding Na^+ ions. [18] In this stage, brief minimization is used to eliminate bad contacts. System and production equilibration are the fourth and fifth steps. To make sure the system has reached the appropriate temperature and pressure, equilibration is carried out in two stages: NPT ensemble and NVT ensemble. Following the download of the equilibration and production input files, the desired modifications are made, including the number of MD run steps, the frequency of trajectory saving, the energy calculation, etc. Long-range electrostatic interactions were handled using the particle mesh Ewald (PME) approach, while non-bonded interactions were dealt with a 12 Å cutoff distance and the neighbor seeking list was buffered using the Verlet cutoff-scheme. The system's energy was minimized using the steepest descent algorithm (5000 steps) prior to production simulation. Then, utilizing 400 kJ mol^{-1} nm^{-2} and 40 kJ mol^{-1} nm^{-2} positional constraints on the backbone and side chains, respectively, the complex was equilibrated for stabilizing its temperature and pressure by exposing it to NVT and NPT ensembles and simulating for 125 ps at 310.15 K temperature. The complex is then put through a 100 ns production simulation run in the NPT ensemble at 310.15 K and 1 bar. Nose-Hoover thermostats were used to regulate the temperature, while Parrinello-Rahman barostats were employed to regulate the pressure. CHARMM-GUI inputs were utilized to limit H-bonds utilizing the LINCS technique. At 310.15 K, the V-rescale thermostat was employed with a coupling constant of 1 ps. Every two ps, the trajectories were saved. For the manufacturing step, 100 ns NPT assembly simulations were run. The complexes underwent a 100ns MD simulation using the GROMACS 2020.2 program. [19] The trajectories of the MD simulations were analysed using GROMACS tools.

References:

1. Bar FMA, Sameti M, Foudah AI, Haque A, Elsbaey M. In vitro and in silico inhibition of COX-2 and 5-LOX by beta-carboline alkaloids from the seeds of Peganum harmala L. *South African Journal of Botany*. 2022;147:926-936. doi:10.1016/j.sajb.2022.03.044
2. Agbadua OG, Kúsz N, Berkecz R, et al. New Insights into the French Paradox: Free Radical Scavenging by Resveratrol Yields Cardiovascular Protective Metabolites. *J Med Chem*. 2025;68(10):10031-10047. doi:10.1021/acs.jmedchem.4c03061
3. Winter CA, Risley EA, Nuss GW. Carrageenin-Induced Edema in Hind Paw of the Rat as an Assay for Antiinflammatory Drugs. *Proc Soc Exp Biol Med*. 1962;111:544-547. doi:10.3181/00379727-111-27849.

4. Zhang QA, Wang X, Song Y, Fan XH, García-Martín JF. Optimization of pyrogallol autoxidation conditions and its application in evaluation of superoxide anion radical scavenging capacity for four antioxidants. *J AOAC Int*. 2016;99(2):504-511. doi:10.5740/jaoacint.15-0223
5. Richards A, Chaurasia S. Antioxidant Activity and Reactive Oxygen Species (ROS) Scavenging Mechanism of Eriodictyon californium, an Edible Herb of North America. *J Chem*. 2022;2022:6980121. doi:10.1155/2022/6980121
6. Litman K, Bouch S, Litvack ML, Post M. Therapeutic characteristics of alveolar-like macrophages in mouse models of hyperoxia and LPS-induced lung inflammation. *Am J Physiol Lung Cell Mol Physiol*. 2024;327(3):L269-L281. doi:10.1152/ajplung.00270.2023
7. Du Y, Fan Y, Li X, Chen F. Novel anti-inflammatory properties of mannose oligosaccharides in the treatment of inflammatory bowel disease via LGALS3 modulation. *NPJ Biofilms Microbiomes*. 2025;11(1):26. doi:10.1038/s41522-025-00648-3
8. Jin F, Lin H, Pan S. Novel therapeutic strategy: Nrf2 activation in targeting senescence-related changes in chronic obstructive pulmonary disease. *J Thorac Dis*. 2025;17(2):623-640. doi:10.21037/jtd-24-710
9. Mosmann T. Rapid Colorimetric Assay for Cellular Growth and Survival: Application to Proliferation and Cytotoxicity Assays. *Journal oflmmunologicalMethods*. 1983;65:55-63. doi:10.1016/0022-1759(83)90303-4
10. Kurumbail RG, Stevens AM, Gierse JK, et al. Structural basis for selective inhibition of cyclooxygenase-2 by anti-inflammatory agents. *Nature*. 1996;384:644-648. doi:10.1038/384644a0
11. Gilbert NC, Gerstmeier J, Schexnaydre EE, et al. Structural and mechanistic insights into 5-lipoxygenase inhibition by natural products. *Nat Chem Biol*. 2020;16(7):783-790. doi:10.1038/s41589-020-0544-7
12. Morris GM, Ruth H, Lindstrom W, et al. Software news and updates AutoDock4 and AutoDockTools4: Automated docking with selective receptor flexibility. *J Comput Chem*. 2009;30(16):2785-2791. doi:10.1002/JCC.21256
13. MarvinSketch (version 23.17, by ChemAxon), Accessed on 5 Jan 2024.
14. Trott O, Olson AJ. Software News and Update AutoDock Vina: Improving the Speed and Accuracy of Docking with a New Scoring Function, Efficient Optimization, and Multithreading. *J Comput Chem*. 2010;31:455-461. doi:10.1002/jcc.21334
15. BIOVIA, Dassault Systèmes, Discovery Studio Visualizer, V21.1.0, San Diego: Dassault Systèmes, 2021.
16. Brooks BR, Brooks CL, Mackerell AD, et al. CHARMM: The biomolecular simulation program. *J Comput Chem*. 2009;30(10):1545-1614. doi:10.1002/jcc.21287
17. Lee J, Cheng X, Swails JM, et al. CHARMM-GUI Input Generator for NAMD, GROMACS, AMBER, OpenMM, and CHARMM/OpenMM Simulations Using the CHARMM36 Additive Force Field. *J Chem Theory Comput*. 2016;12(1):405-413. doi:10.1021/acs.jctc.5b00935
18. Vanommeslaeghe K, Hatcher E, Acharya C, et al. CHARMM general force field: A force field for drug-like molecules compatible with the CHARMM all-atom additive biological force fields. *J Comput Chem*. 2010;31(4):671-690. doi:10.1002/jcc.21367
19. Abraham MJ, Murtola T, Schulz R, et al. Gromacs: High performance molecular simulations through multi-level parallelism from laptops to supercomputers. *SoftwareX*. 2015;1-2:19-25. doi:10.1016/j.softx.2015.06.001

Docking supporting material

Table (S1): Molecular docking details of target compounds with 1CX2					
**Code**	**Docking scores (Kcal/mol)**	**Amino acids interactions**	**Binding group**	**Type of bond**	**H- bond length (Å)**
**5a**	-9.3	Tyr355	triazole (N=)	H-bond	2.81
		Arg513	central phenyl ring	Pi-cation	-
		Val523	central phenyl ring	Pi-sigma	-
		Ser353	central phenyl ring	Pi-sigma	-
		Leu531	triazolo[1,5-a]pyrimidine	Pi-alkyl	-
		Val349	triazolo[1,5-a]pyrimidine	Pi-sigma	-
		Ala527	triazolo[1,5-a]pyrimidine	Pi-sigma	-
		Asp515	styryl ring	Pi-amide	-
		Ala516	styryl ring	Pi-alkyl	-
**5b**	-7.4	Tyr355	triazole (N=)	H-bond	2.81
		Arg513	central phenyl ring	Pi-cation	-
			chlorine	Pi-alkyl	-
		Val523	central phenyl ring	Pi-sigma	-
		Ser353			

		Leu531	central phenyl ring	Pi-sigma	-
		Val349	triazolo[1,5-a]pyrimidine	Pi-alkyl	-
		Ala527	triazolo[1,5-a]pyrimidine	Pi-sigma	-
		Asp515	triazolo[1,5-a]pyrimidine	Pi-sigma	-
		Ala516	styryl ring	Pi-amide	-
			styryl ring	Pi-alkyl	-
		Pro514	chlorine	Pi-alkyl	-
		His90	chlorine	Pi-alkyl	-
			chlorine	Pi-alkyl	-
**5c**	-8.7	Tyr355	triazole (N=)	H-bond	2.83
		Arg513	central phenyl ring	Pi-cation	-
			chlorine	Pi-alkyl	-
		Val523	central phenyl ring	Pi-sigma	-
		Ser353	central phenyl ring	Pi-sigma	-
		Leu531	triazolo[1,5-a]pyrimidine	Pi-alkyl	-
		Val349	triazolo[1,5-a]pyrimidine	Pi-sigma	-
		Ala527	triazolo[1,5-a]pyrimidine	Pi-sigma	-
		Asp515	styryl ring	Pi-amide	-

		Ala516	styryl ring	Pi-alkyl	-
			chlorine	Pi-alkyl	-
		Pro514	chlorine	Pi-alkyl	-
		His90	chlorine	Pi-alkyl	-
**5d**	-7.2	Tyr355	triazole (N=)	H-bond	2.86
		Arg120	triazolo[1,5-a]pyrimidine	Pi-cation	-
		Val523	central phenyl ring	Pi-sigma	-
		Ser353	central phenyl ring	Pi-sigma	-
		Leu531	triazolo[1,5-a]pyrimidine	Pi-alkyl	-
		Val349	triazolo[1,5-a]pyrimidine	Pi-sigma	-
		Ala527	triazolo[1,5-a]pyrimidine	Pi-sigma	-
		Asp515	styryl ring	Pi-amide	-
		Ala516	styryl ring	Pi-alkyl	-
**5e**	-7.2	Tyr355	triazole (N=)	H-bond	2.83
		Arg513	central phenyl ring	Pi-cation	-
		Val523	central phenyl ring	Pi-sigma	-
		Ser353	central phenyl ring	Pi-sigma	-
		Leu531	triazolo[1,5-a]pyrimidine	Pi-alkyl	-

		Val349	triazolo[1,5-a]pyrimidine	Pi-sigma	-
		Ala527	triazolo[1,5-a]pyrimidine	Pi-sigma	-
		Asp515	styryl ring	Pi-amide	-
		Ala516	styryl ring	Pi-alkyl	-
**5f**	-6.2	Tyr355	triazole (N=)	H-bond	2.81
		Arg513	central phenyl ring	Pi-cation	-
		Val523	central phenyl ring	Pi-sigma	-
		Ser353	central phenyl ring	Pi-sigma	-
		Leu531	triazolo[1,5-a]pyrimidine	Pi-alkyl	-
		Val349	triazolo[1,5-a]pyrimidine	Pi-sigma	-
		Ala527	triazolo[1,5-a]pyrimidine	Pi-sigma	-
		Asp515	thienyl ring	Pi-amide	-
		Ala516	thienyl ring	Pi-alkyl	-
**5g**	-7.8	Tyr355	triazole (N=)	H-bond	2.77
		Arg513	central phenyl ring	Pi-cation	-
		Val523	central phenyl ring	Pi-sigma	-
		Ser353	central phenyl ring	Pi-sigma	-
		Ile517	Central phenyl(C=O	H-bond	2.90

		Leu531	triazolo[1,5-a]pyrimidine	Pi-alkyl	-
		Val349	triazolo[1,5-a]pyrimidine	Pi-sigma	-
		Ala527	triazolo[1,5-a]pyrimidine	Pi-sigma	-
		Asp515	naphthyl ring	Pi-amide	-
		Ala516	naphthyl ring	Pi-alkyl	-
		Pro514	naphthyl ring	Pi-alkyl	-
		Thr94	naphthyl ring	Pi-sigma	-
**6a**	-6.4	Tyr355	triazole (N=)	H-bond	2.76
		Arg513	Central phenyl ring	Pi-cation	-
		Val523	Central phenyl ring	Pi-sigma	-
		Leu531	triazolo[1,5-a]pyrimidine	Pi-alkyl	-
		Val349	triazolo[1,5-a]pyrimidine	Pi-sigma/ Pi-alkyl	-
		Val116	triazolo[1,5-a]pyrimidine	Pi-alkyl	-
		Leu359	triazolo[1,5-a]pyrimidine	Pi-alkyl	-
		Ala527	triazolo[1,5-a]pyrimidine	Pi-alkyl	-
		His90	Chlorophenyl ring	Pi-cation	-
		Ala516	Chlorophenyl ring	Pi-alkyl	-
		Gln192	4,5-dihydropyrazolyl	H-bond	1.88

**6b**	-6.3	Arg513	Central phenyl ring	Pi-cation	-
			Chlorine	Pi-alkyl	-
		Val523	Central phenyl ring	Pi-sigma	-
		Ser353	Central phenyl ring	Pi-sigma	-
		Leu531	triazolo[1,5-a]pyrimidine	Pi-alkyl	-
		Val349	triazolo[1,5-a]pyrimidine	Pi-sigma/ Pi-alkyl	-
		Ala527	triazolo[1,5-a]pyrimidine	Pi-sigma/ Pi-alkyl	-
		His90	Chlorine	Pi-alkyl	-
		Pro514	Chlorine	Pi-alkyl	-
		Ala516	Chlorophenyl ring	Pi-alkyl	-
			Chlorine	Pi-alkyl	-
		Gln192	4,5-dihydropyrazolyl	H-bond	1.80
**6c**	-7.2	Tyr355	triazole (N=)	H-bond	2.59
		Arg513	Central phenyl ring	Pi-cation	-
			Chlorophenyl ring	Pi-alkyl	-
		Val523	Central phenyl ring	Pi-sigma	-
		Ser353	Central phenyl ring	Pi-sigma	-
		Leu359	triazolo[1,5-a]pyrimidine	Pi-alkyl	-

		Leu531	triazolo[1,5-a]pyrimidine	Pi-alkyl	-
		Val349	triazolo[1,5-a]pyrimidine	Pi-sigma/ Pi-alkyl	-
		Ala527	triazolo[1,5-a]pyrimidine	Pi-sigma/ Pi-alkyl	-
		His90	Chlorophenyl ring	Pi-cation	-
		Pro514	Chlorophenyl ring	Pi-alkyl	-
			Chlorine	Pi-alkyl	-
		Ala516	Chlorophenyl ring	Pi-alkyl	-
		Gly354	chlorine	H-bond	3.29
		Gln192	4,5-dihydropyrazolyl	H-bond	1.73
**6d**	-8.2	Tyr355	triazole (N=)	H-bond	2.86
		Arg513	4,5-dihydropyrazolyl (N=)	H-bond	2.83
		Val523	Central phenyl ring	Pi-sigma	-
		Ser353	Central phenyl ring	Pi-sigma	-
		Val116	triazolo[1,5-a]pyrimidine	Pi-alkyl	-
		Leu531	triazolo[1,5-a]pyrimidine	Pi-alkyl	-
		Ala527	triazolo[1,5-a]pyrimidine	Pi-alkyl	-
		Val349	triazolo[1,5-a]pyrimidine	Pi-sigma/ Pi-alkyl	-
		His90	nitrophenyl ring	Pi-cation	-

		Ala516	nitrophenyl ring	Pi-alkyl	-
		Gln192	4,5-dihydropyrazolyl (CH)	H-bond	3.29
**6e**	-6.6	Arg513	Central phenyl ring	Pi-cation	-
			bromophenyl ring	Pi-alkyl	-
		Ser353	Central phenyl ring	Pi- sigma	-
		Val523	Central phenyl ring	Pi-sigma	-
			triazolo[1,5-a]pyrimidine	Pi-alkyl	-
		Leu531	triazolo[1,5-a]pyrimidine	Pi-alkyl	-
		Ala527	triazolo[1,5-a]pyrimidine	Pi- sigma	-
		Val349	triazolo[1,5-a]pyrimidine	Pi-sigma	-
		Ser530	triazolo[1,5-a]pyrimidine (N=)	H-bond	3.56
		Trp387	pyrimidine (CH_3)	Pi-alkyl	-
		Met522	pyrimidine (CH_3)	Pi-alkyl	-
		Phe518	pyrimidine (CH_3)	Pi-alkyl	-
		His90	bromophenyl ring	Pi-cation	-
			bromine	Pi-alkyl	-
		His95	bromine	Pi-alkyl	-
		Ala516	bromophenyl ring	Pi-alkyl	-

				Pi-cation	
		Arg513	Central phenyl ring	H-bond	-
			4,5-dihydropyrazolyl (N=)	Pi- sigma	2.84
		Ser353	Central phenyl ring	Pi- sigma	-
		Val523	Central phenyl ring	Pi-alkyl	-
		Leu531	triazolo[1,5-a]pyrimidine	Pi-alkyl	-
		Ala527	triazolo[1,5-a]pyrimidine	Pi-sigma	-
**6f**	-7.5	Val349	triazolo[1,5-a]pyrimidine	Pi-alkyl	-
		Leu359	pyrimidine (CH_3)	Pi-alkyl	-
		Tyr355	pyrimidine (CH_3)	Pi-alkyl	-
		Val116	pyrimidine (CH_3)	Pi-cation	-
		His90	thienyl ring	Pi-alkyl	-
		Ala516	thienyl ring	H-bond	-
		Gln192	4,5-dihydropyrazolyl (CH)		3.29
		Arg513	Central phenyl ring	Pi-cation	-
**6g**	-7.9		naphthyl ring	Pi-amide	-
		Ser353	Central phenyl ring	Pi- sigma	-

		Val523	Central phenyl ring	Pi- sigma	-
			triazolo[1,5-a]pyrimidine	Pi-alkyl	-
		Val349	triazolo[1,5-a]pyrimidine	Pi- sigma	-
		Leu531	triazolo[1,5-a]pyrimidine	Pi-alkyl	-
		Ala527	triazolo[1,5-a]pyrimidine	Pi- sigma	-
		Ser530	triazolo[1,5-a]pyrimidine (N=)	H-bond	3.62
		Trp387	pyrimidine (CH_3)	Pi-alkyl	-
		His90	naphthyl ring	Pi-Pi	-
		Thr94	naphthyl ring	Pi-sigma	-
		Ala516	naphthyl ring	Pi-alkyl	-

Table (S2): Molecular docking details of target compounds with 6N2W					
**Code**	**Docking scores (Kcal/mol)**	**Amino acids interactions**	**Binding group**	**Type of bond**	**H- bond length (Å)**
**5a**	-7.8	Phe359	triazolo[1,5-a]pyrimidine	Pi-Pi	-
			triazolo[1,5-a]pyrimidine	Pi-alkyl	-
		Arg596	triazolo[1,5-a]pyrimidine	H-bond	2.98
		Trp599	triazolo[1,5-a]pyrimidine	Pi-alkyl	-
		Ala603	triazolo[1,5-a]pyrimidine	Pi-alkyl	-
		His600	triazolo[1,5-a]pyrimidine	Pi-alkyl	-
		Val604	triazolo[1,5-a]pyrimidine	Pi-alkyl	-
		Leu368	Central phenyl ring	Pi-alkyl	-
		Gln363	Central phenyl ring (CH)	H-bond	4.16
		Ala410	Chlorophenyl ring	Pi-sigma	-
		Lys409	Chlorophenyl ring	Pi- amide	-
			Chlorine	Pi-alkyl	-
**6c**	-7.9	Phe359		Pi-Pi	-
		Arg596	triazolo[1,5-a]pyrimidine	H-bond	3.03
		Trp599	triazolo[1,5-a]pyrimidine	Pi-Pi	-

			triazolo[1,5-a]pyrimidine	Pi-alkyl	-
		Ala603	triazolo[1,5-a]pyrimidine	Pi-alkyl	-
		His600	triazolo[1,5-a]pyrimidine	Pi-alkyl	-
		Val604	triazolo[1,5-a]pyrimidine	Pi-alkyl	-
		His432	triazolo[1,5-a]pyrimidine	H-bond	3.75
		Ile406	triazolo[1,5-a]pyrimidine(CH)	Pi-sigma	-
		Ala410	dichlorophenyl ring	Pi-alkyl	-
		Lys409	dichlorophenyl ring	Pi-alkyl	-
		Leu607	Chlorine	Pi-alkyl	-
			Chlorine		
**6d**	-8.0	Phe359	triazolo[1,5-a]pyrimidine	Pi-Pi	-
			triazolo[1,5-a]pyrimidine	Pi-alkyl	-
		Arg596	triazolo[1,5-a]pyrimidine	H-bond	3.00
		Trp599	triazolo[1,5-a]pyrimidine	Pi-alkyl	-
		Ala603	triazolo[1,5-a]pyrimidine	Pi-alkyl	-
		His600	triazolo[1,5-a]pyrimidine	Pi-alkyl	-
		Val604	triazolo[1,5-a]pyrimidine	Pi-alkyl	-

		Gln363	Central phenyl ring (CH)	H-bond	3.91
		Ile673	4,5-dihydropyrazolyl (NH)	H-bond	2.40
		Ala410	nitrophenyl ring	Pi-alkyl	-
		Ile406	nitrophenyl ring	Pi-sigma	-

Table (S3): The Drug-likeness, physicochemical properties of the most active derivatives (**5a, 6c,** and **6d)** and positive controls (celecoxib and diclofenac sodium) using the SwissADME web tool.

**Test items**	**The most active derivatives 5a, 6c, 6d, and positive controls (celecoxib and diclofenac sodium)**				
	**5a**	**6c**	**6d**	**celecoxib**	**Diclofenac sodium**
	**Molecular properties**				
**M. Wt. (≤ 500) [a]**	389.84	438.31	414.42	381.37	318.13
**C-Sp^3 (≥ 0.25)**	0.05	0.14	0.14	0.12	0.07
**NRB (≤ 10) [b]**	5	4	5	4	5
**TPSA (Å^2) (≤ 140) [b]**	72.18	79.50	125.32	86.36	38.33
**nHBA (≤ 10.0) [a]**	4	4	6	7	2
**nHBD (≤ 5.0) [a]**	1	2	2	1	1
**MLog P (≤ 4.15) [a]**	3.38	3.95	2.13	2.65	3.84
**Log S (ESOL) (-6 to 0)**	-5.49	-5.98	-4.85	-4.57	-4.76
**Water Solubility**	Moderately soluble	Moderately soluble	Moderately soluble	Moderately soluble	Moderately soluble
**XLogP3 (-0.7 to 5.0)**	4.77	5.03	3.61	3.40	4.49
	**Drug likeness and Medicinal Chemistry**				
**Lipinski Rule (violation) [a]**	Yes (0)	Yes (0)	Yes (0)	Yes (0)	Yes (0)
**Veber Rule (violation) [b]**	Yes	Yes	Yes	Yes	Yes
**PAINS**	0 alert	0 alert	0 alert	0 alert	0 alert
**Synthetic accessibility**	3.17	3.87	3.91	2.74	2.39
**Bioavailability Score**	0.55	0.55	0.55	0.55	0.55
	**Pharmacokinetics**				
**GI absorption**	High	High	High	High	High
**BBB permeant**	Yes	No	No	No	Yes
**p-gp substrate**	No	No	Yes	No	No

a = refer to criteria of Lipinski rule; **b** = refer to criteria of Veber rule

Table (S4): Toxicity prediction of the most active triazolopyrimidine derivatives (**5a, 6c, and 6d)** and positive controls (celecoxib and diclofenac sodium) using the Protox-3 web tool.

Oral toxicity prediction	**The most active derivatives 5a, 6c, 6d, and positive controls (celecoxib and diclofenac sodium)**				
	**5a**	**6c**	**6d**	**celecoxib**	**Diclofenac sodium**
**LD_{50} mg/kg**	300	1000	800	1400	56
**Toxicity Class**	3	4	4	4	3
	**Oral toxicity prediction/(Probability)**				
**Hepatotoxicity [a]**	Active (0.62)	Active (0.53)	Active (0.57)	**Inactive (0.60)**	Active (0.67)
**Nephrotoxicity [a]**	**Inactive (0.79)**	**Inactive (0.68)**	**Inactive (0.59)**	**Inactive (0.76)**	Active (0.52)
**Cardiotoxicity [a]**	**Inactive (0.85)**	**Inactive (0.88)**	**Inactive (0.79)**	**Inactive (0.83)**	**Inactive (0.63)**
**Immunotoxicity [b]**	Active (0.92)	**Inactive (0.97)**	**Inactive (0.97)**	**Inactive (0.99)**	**Inactive (0.93)**
**Mutagenicity [b]**	Active (0.56)	**Inactive (0.54)**	Active (0.86)	**Inactive (0.75)**	**Inactive (0.62)**
**Cytotoxicity [b]**	**Inactive (0.74)**	**Inactive (0.68)**	**Inactive (0.73)**	**Inactive (0.91)**	**Inactive (0.72)**
**Ecotoxicity [b]**	Active (0.73)	Active (0.51)	**Inactive (0.58)**	**Inactive (0.72)**	Active (0.69)
**Nutritional toxicity [b]**	**Inactive (0.54)**	**Inactive (0.54)**	**Inactive (0.63)**	**Inactive (0.61)**	**Inactive (0.72)**
**Androgen Receptor (AR) [c]**	**Inactive (0.99)**	**Inactive (0.97)**	**Inactive (0.98)**	**Inactive (1.0)**	**Inactive (0.96)**
**Aromatase [c]**	**Inactive (0.66)**	**Inactive (0.68)**	**Inactive (0.85)**	**Inactive (0.93)**	**Inactive (0.93)**
**Estrogen Receptor Alpha [c] (ER)**	**Inactive (0.74)**	**Inactive (0.84)**	**Inactive (0.82)**	Active (1.0)	**Inactive (0.82)**
**PPAR-Gamma [c]**	**Inactive (0.95)**	**Inactive (0.92)**	**Inactive (0.94)**	**Inactive (0.98)**	**Inactive (0.71)**
a = organ toxicity, b = toxicity endpoint, and c = Tox21 Nuclear receptor signaling pathways for organ					

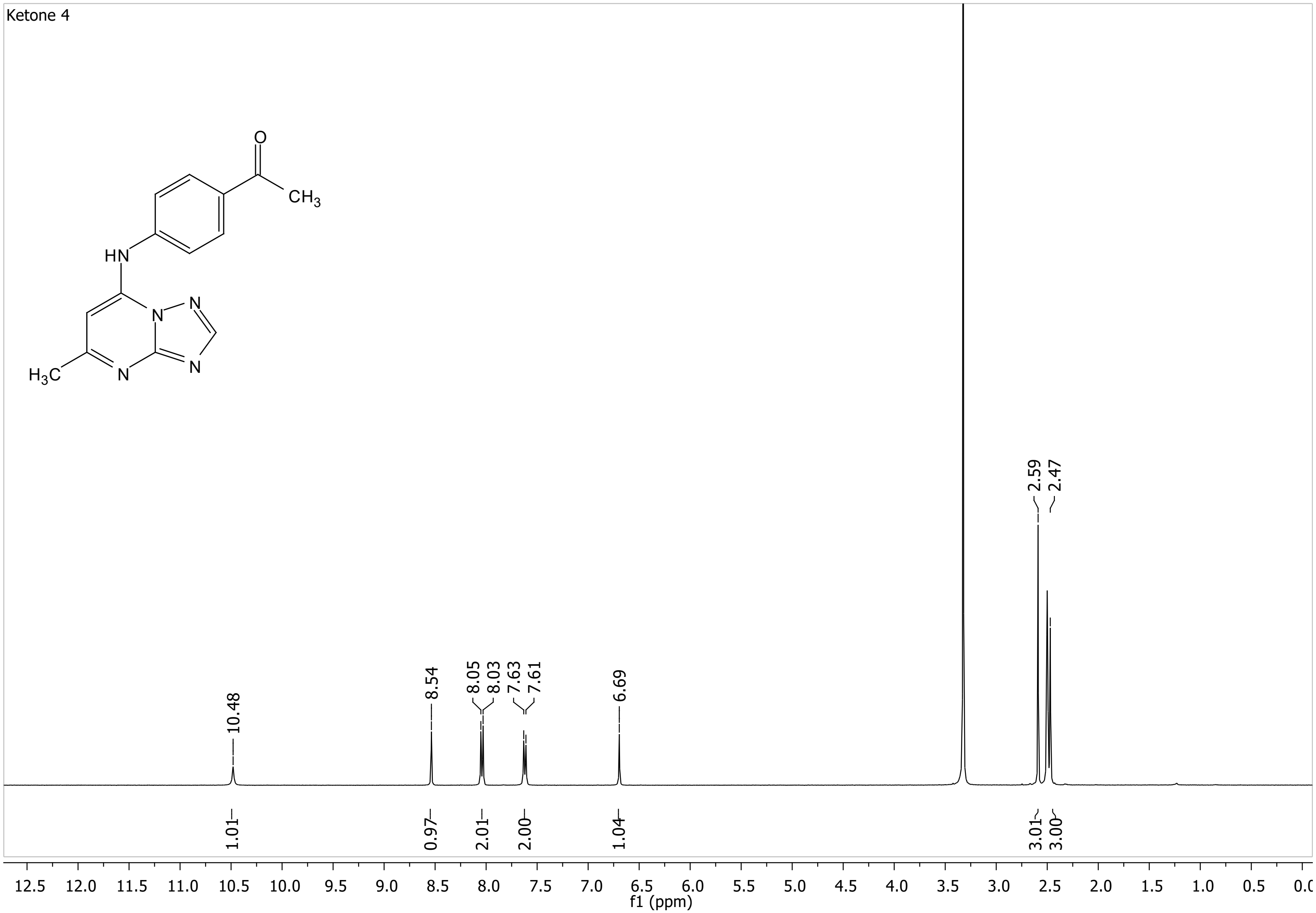

Ketone 4
O
CH3
HN
N
N
N
N
H3C
10.48
8.54
8.05
8.03
7.63
7.61
6.69
2.59
2.47
1.01
0.97
2.01
2.00
1.04
3.01
3.00
12.5 12.0 11.5 11.0 10.5 10.0 9.5 9.0 8.5 8.0 7.5 7.0 6.5 6.0 5.5 5.0 4.5 4.0 3.5 3.0 2.5 2.0 1.5 1.0 0.5
f1 (ppm)

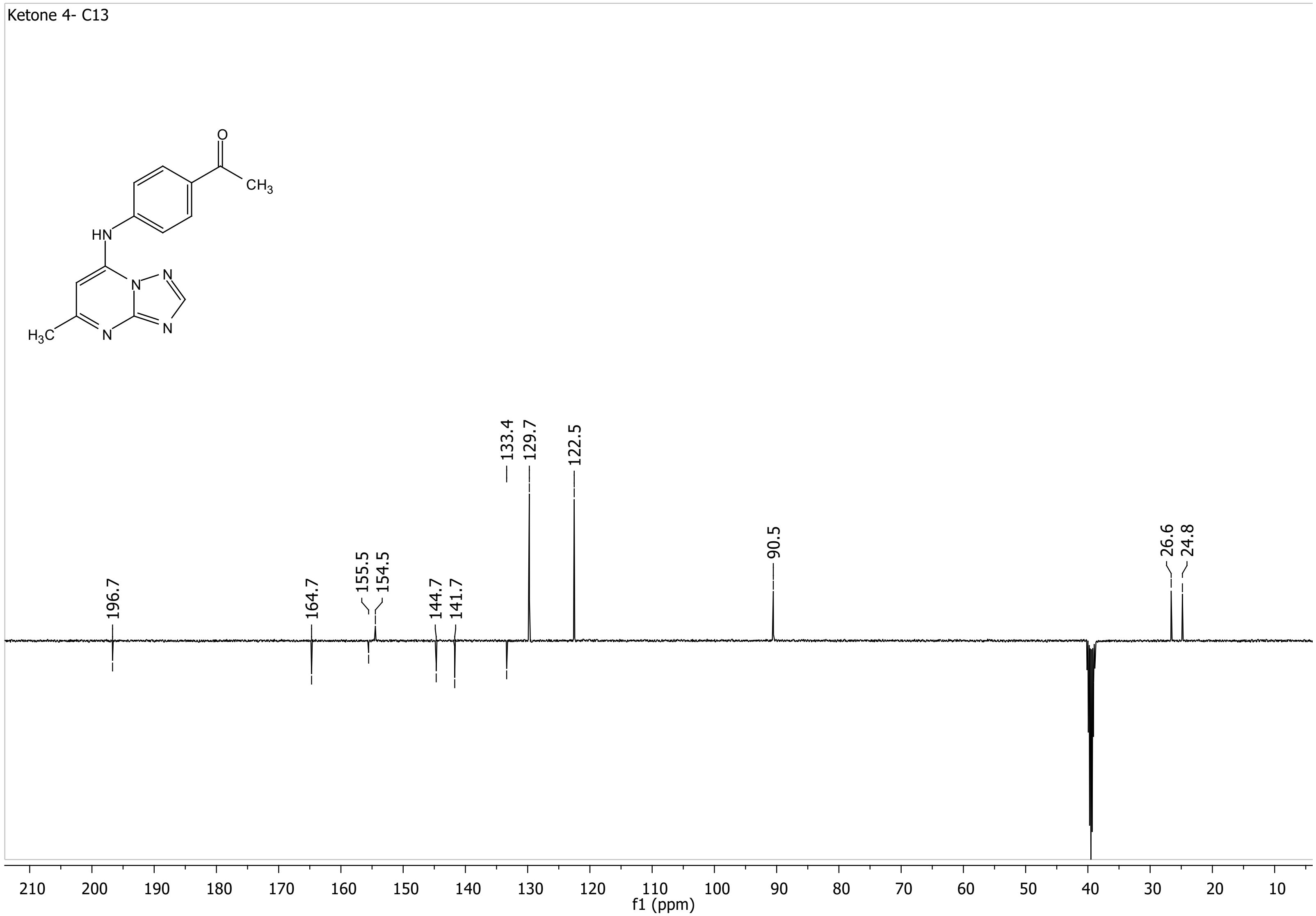

Ketone 4- C13
O
CH3
HN
N
N
N
N
H3C
196.7
164.7
155.5
154.5
144.7
141.7
133.4
129.7
122.5
90.5
26.6
24.8
210 200 190 180 170 160 150 140 130 120 110 100 90 80 70 60 50 40 30 20 10
f1 (ppm)

5a (4-Cl)

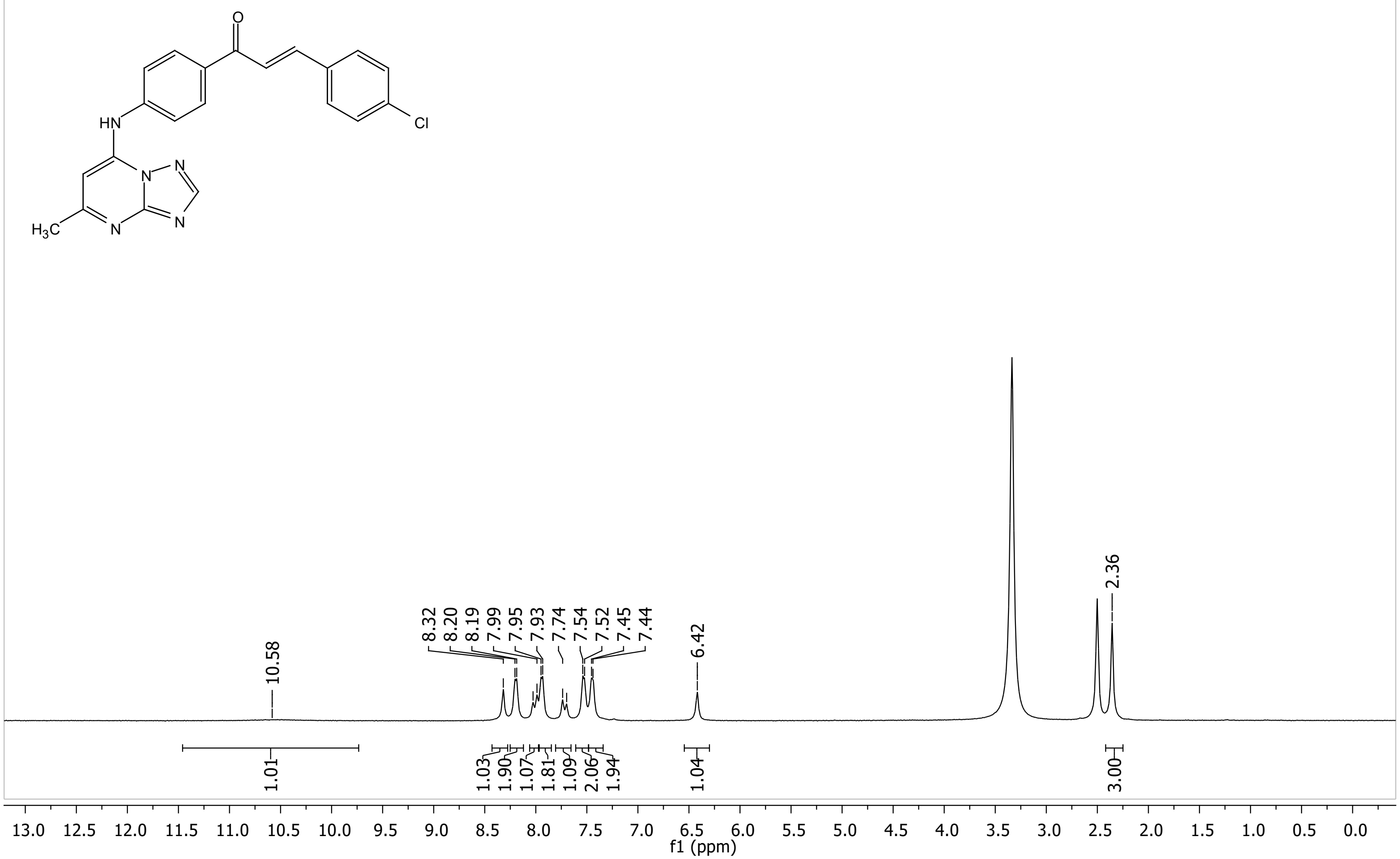

O
HN
Cl
N
N
N
N
H_3C
10.58
8.32
8.20
8.19
7.99
7.95
7.93
7.74
7.54
7.52
7.45
7.44
6.42
2.36
1.01
1.03
1.90
1.07
1.81
1.09
2.06
1.94
1.04
3.00
13.0 12.5 12.0 11.5 11.0 10.5 10.0 9.5 9.0 8.5 8.0 7.5 7.0 6.5 6.0 5.5 5.0 4.5 4.0 3.5 3.0 2.5 2.0 1.5 1.0 0.5 0.0
f1 (ppm)

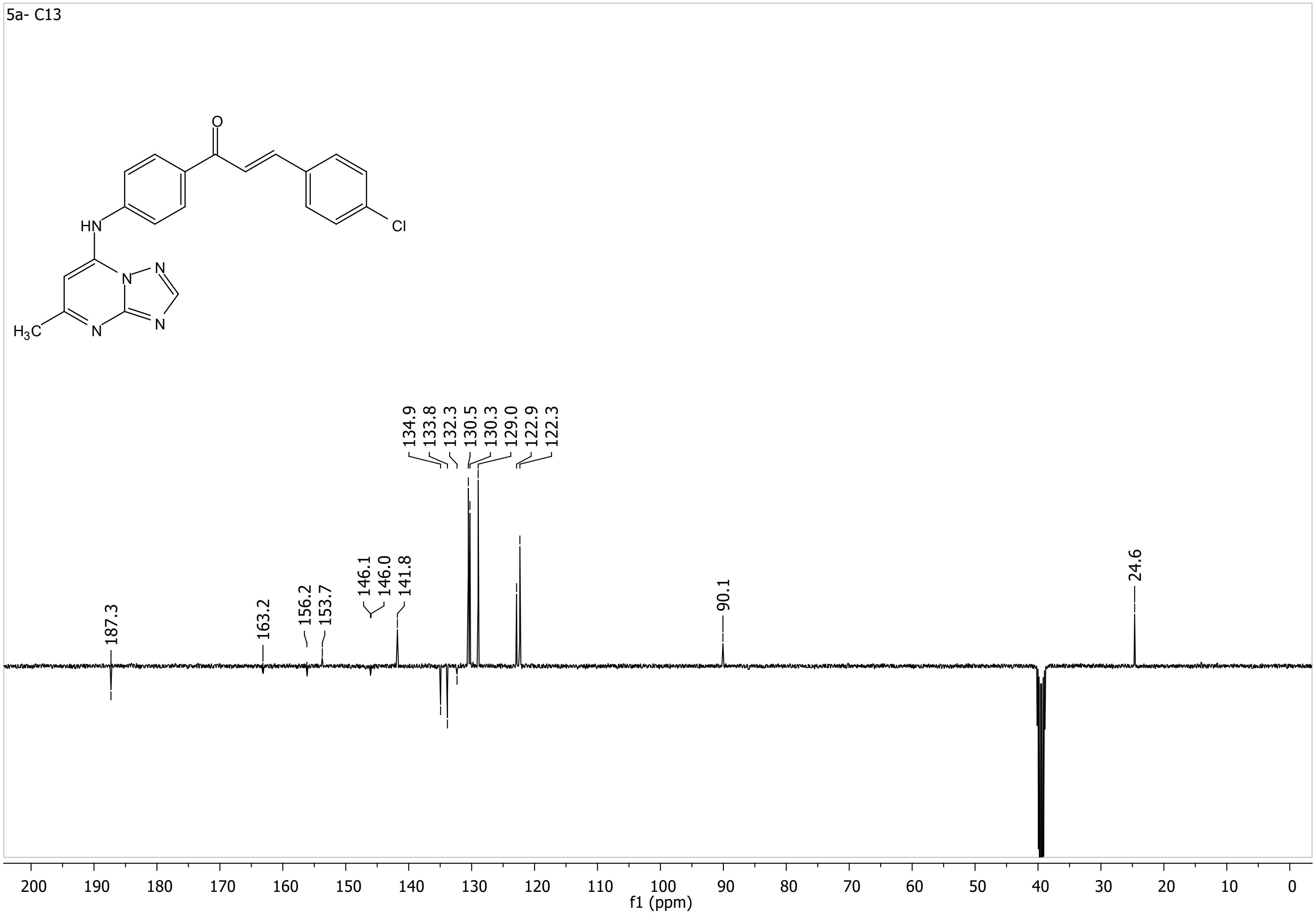
5a- C13
O
HN
Cl
N
N
N
N
H_3C
187.3
163.2
156.2
153.7
146.1
146.0
141.8
134.9
133.8
132.3
130.5
130.3
129.0
122.9
122.3
90.1
24.6
200 190 180 170 160 150 140 130 120 110 100 90 80 70 60 50 40 30 20 10 0
f1 (ppm)

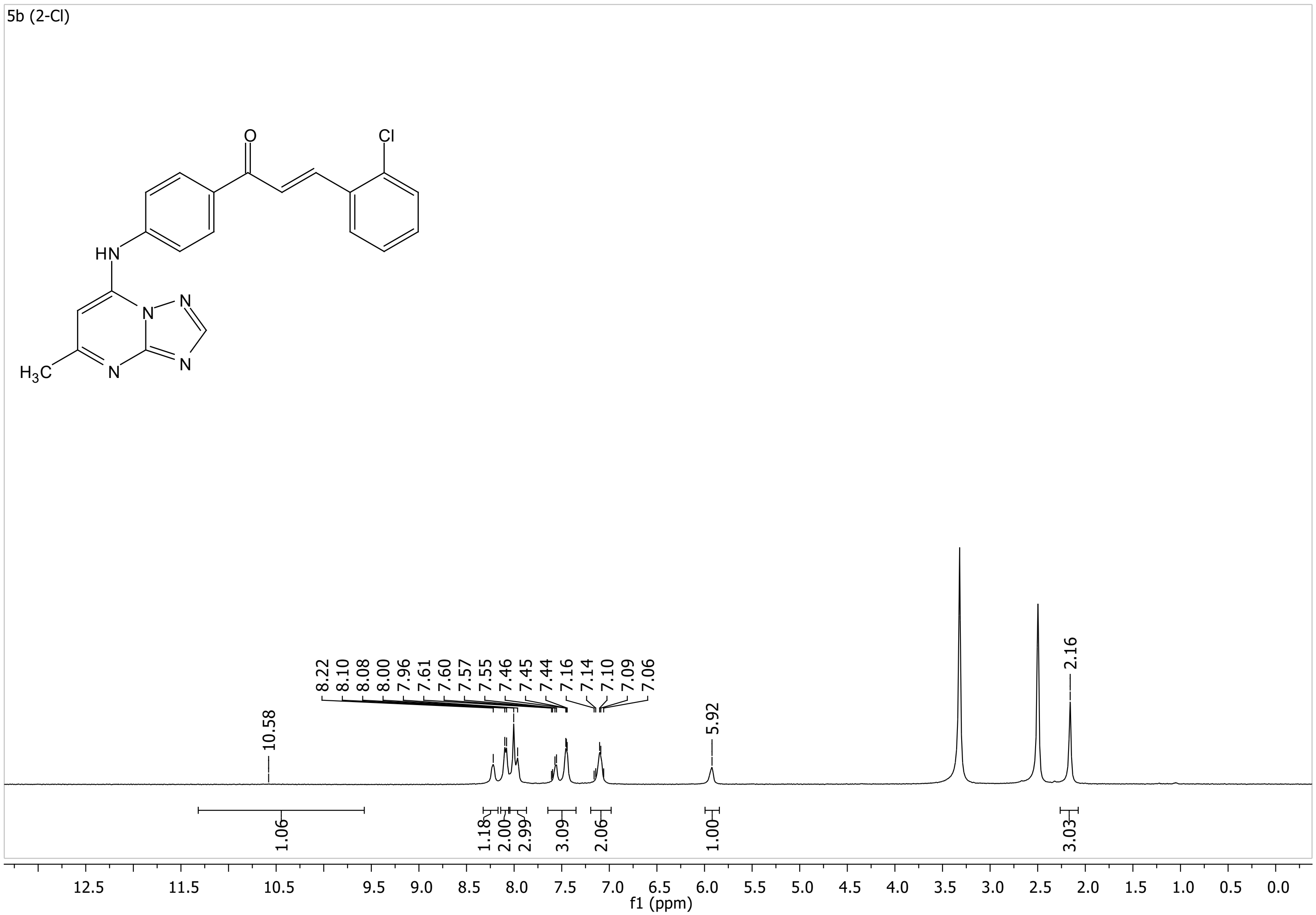
5b (2-Cl)
O
Cl
HN
N
N
N
N
H3C
10.58
8.22
8.10
8.08
8.00
7.96
7.61
7.60
7.57
7.55
7.46
7.45
7.44
7.16
7.14
7.10
7.09
7.06
5.92
2.16
1.06
1.18
2.00
2.99
3.09
2.06
1.00
3.03
12.5
11.5
10.5
9.5
9.0
8.5
8.0
7.5
7.0
6.5
6.0
5.5
5.0
4.5
4.0
3.5
3.0
2.5
2.0
1.5
1.0
0.5
0.0
f1 (ppm)

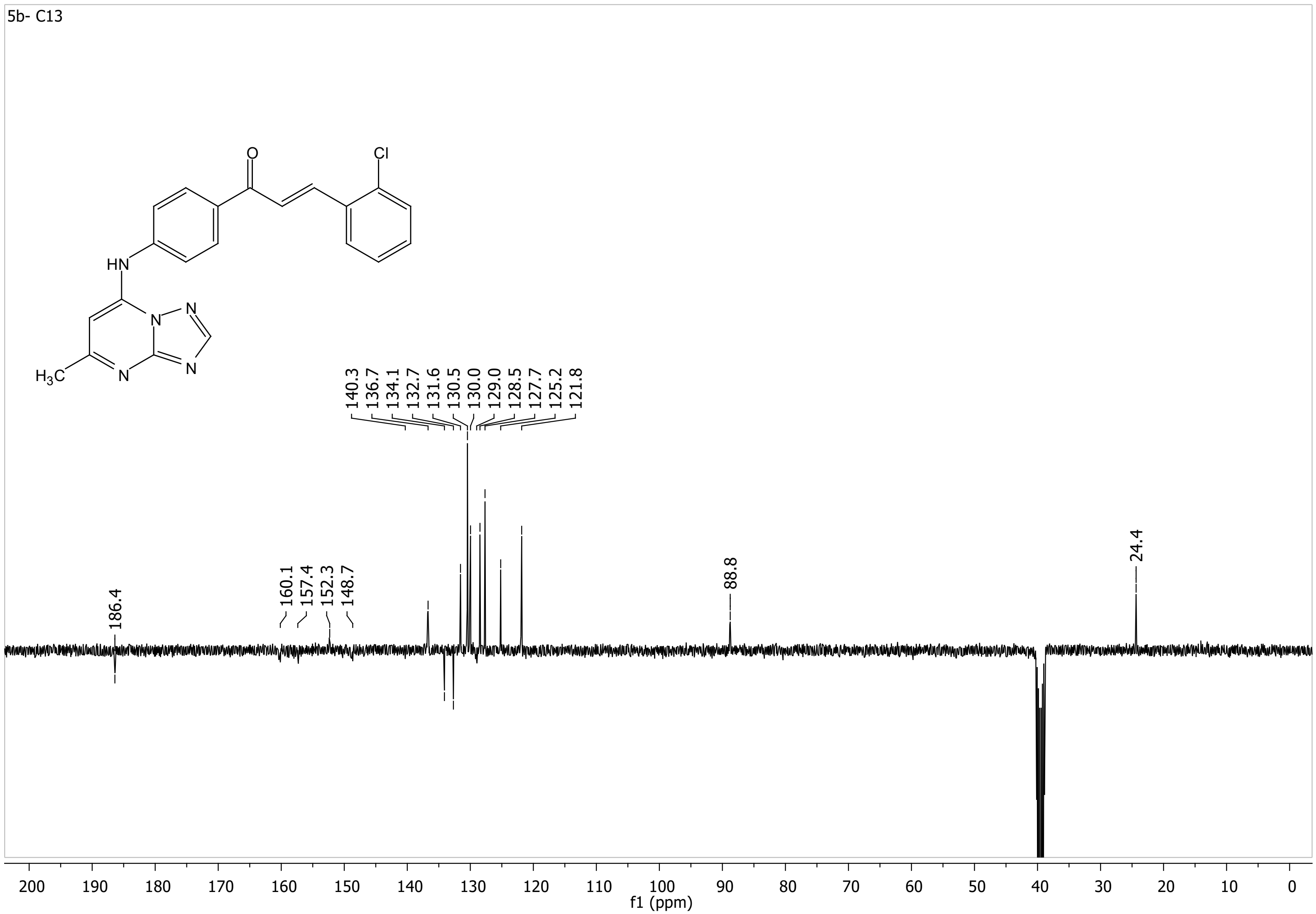
5b- C13
O
Cl
HN
N
N
N
N
H3C
140.3
136.7
134.1
132.7
131.6
130.5
130.0
129.0
128.5
127.7
125.2
121.8
186.4
160.1
157.4
152.3
148.7
88.8
24.4
200 190 180 170 160 150 140 130 120 110 100 90 80 70 60 50 40 30 20 10 0
f1 (ppm)

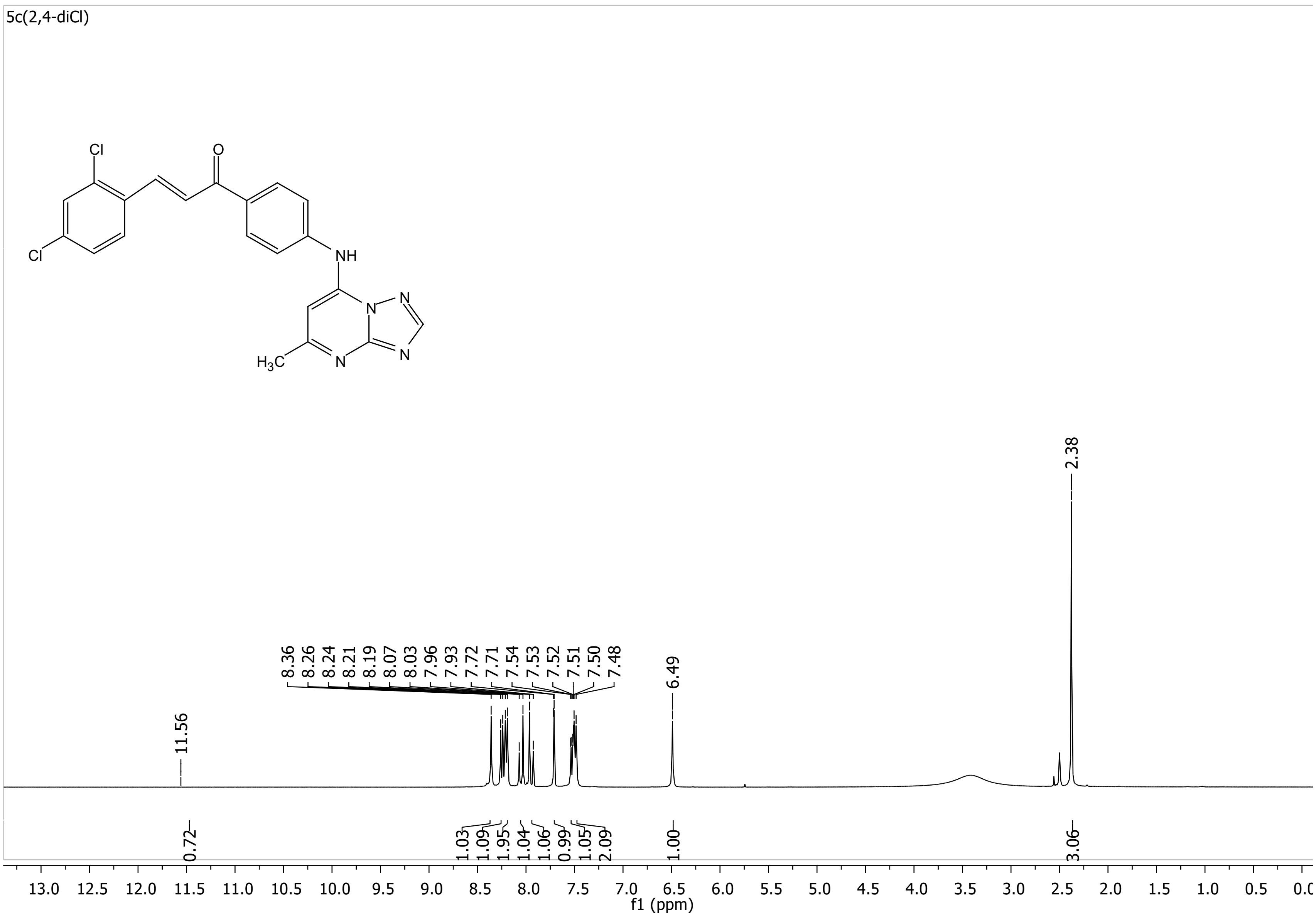
5c(2,4-diCl)
Cl
O
Cl
NH
N
N
N
N
H_3C
8.36 8.26 8.24 8.21 8.19 8.07 8.03 7.96 7.93 7.72 7.71 7.54 7.53 7.52 7.51 7.50 7.48
6.49
11.56
2.38
0.72
1.03 1.09 1.95 1.04 1.06 0.99 1.05 2.09
1.00
3.06
13.0 12.5 12.0 11.5 11.0 10.5 10.0 9.5 9.0 8.5 8.0 7.5 7.0 6.5 6.0 5.5 5.0 4.5 4.0 3.5 3.0 2.5 2.0 1.5 1.0 0.5 0.0
f1 (ppm)

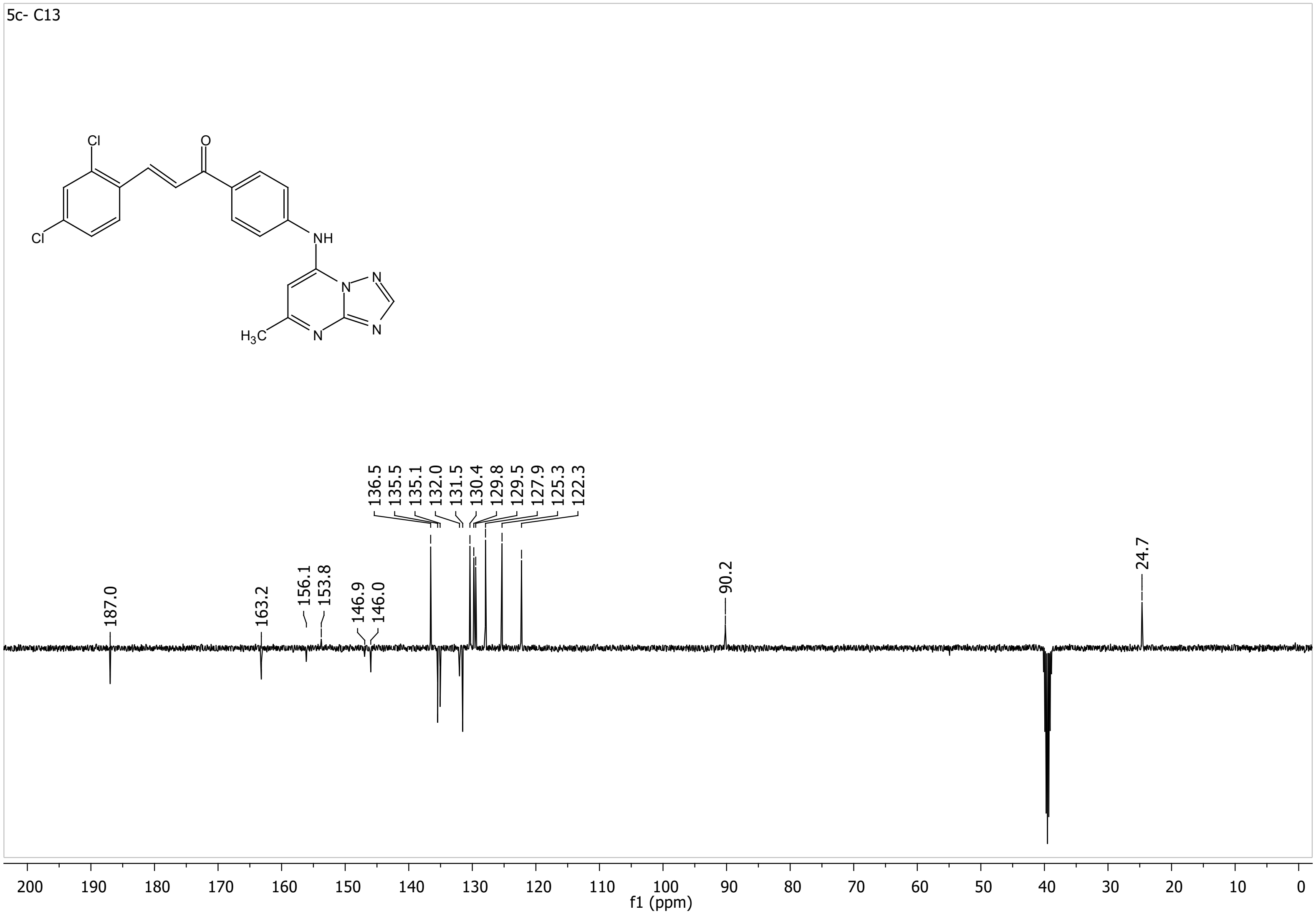
5c- C13
Cl
O
Cl
NH
N
N
N
N
H3C
136.5
135.5
135.1
132.0
131.5
130.4
129.8
129.5
127.9
125.3
122.3
187.0
163.2
156.1
153.8
146.9
146.0
90.2
24.7
200 190 180 170 160 150 140 130 120 110 100 90 80 70 60 50 40 30 20 10 0
f1 (ppm)

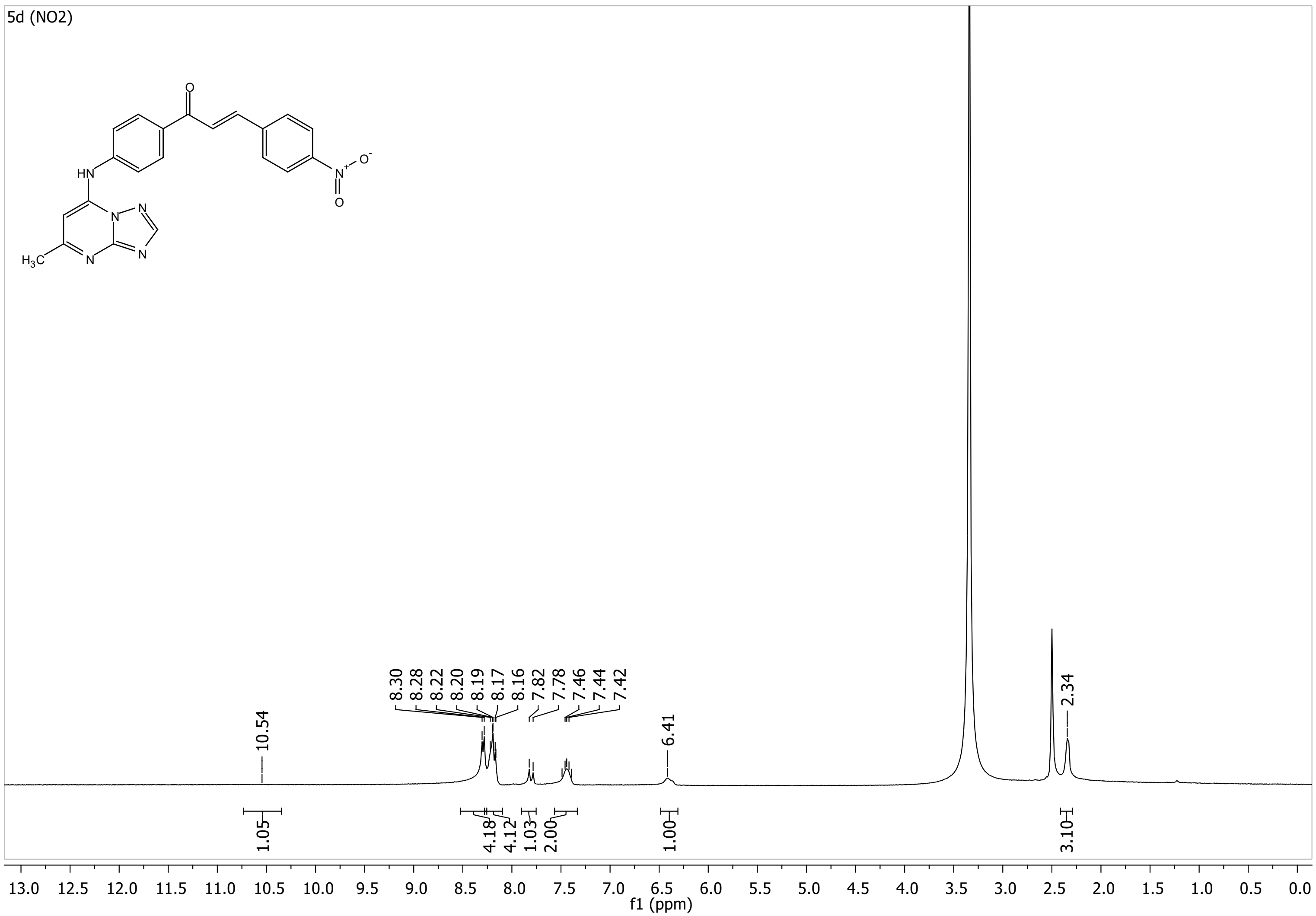

5d (NO2)
10.54
8.30
8.28
8.22
8.20
8.19
8.17
8.16
7.82
7.78
7.46
7.44
7.42
6.41
2.34
1.05
4.18
4.12
1.03
2.00
1.00
3.10
f1 (ppm)

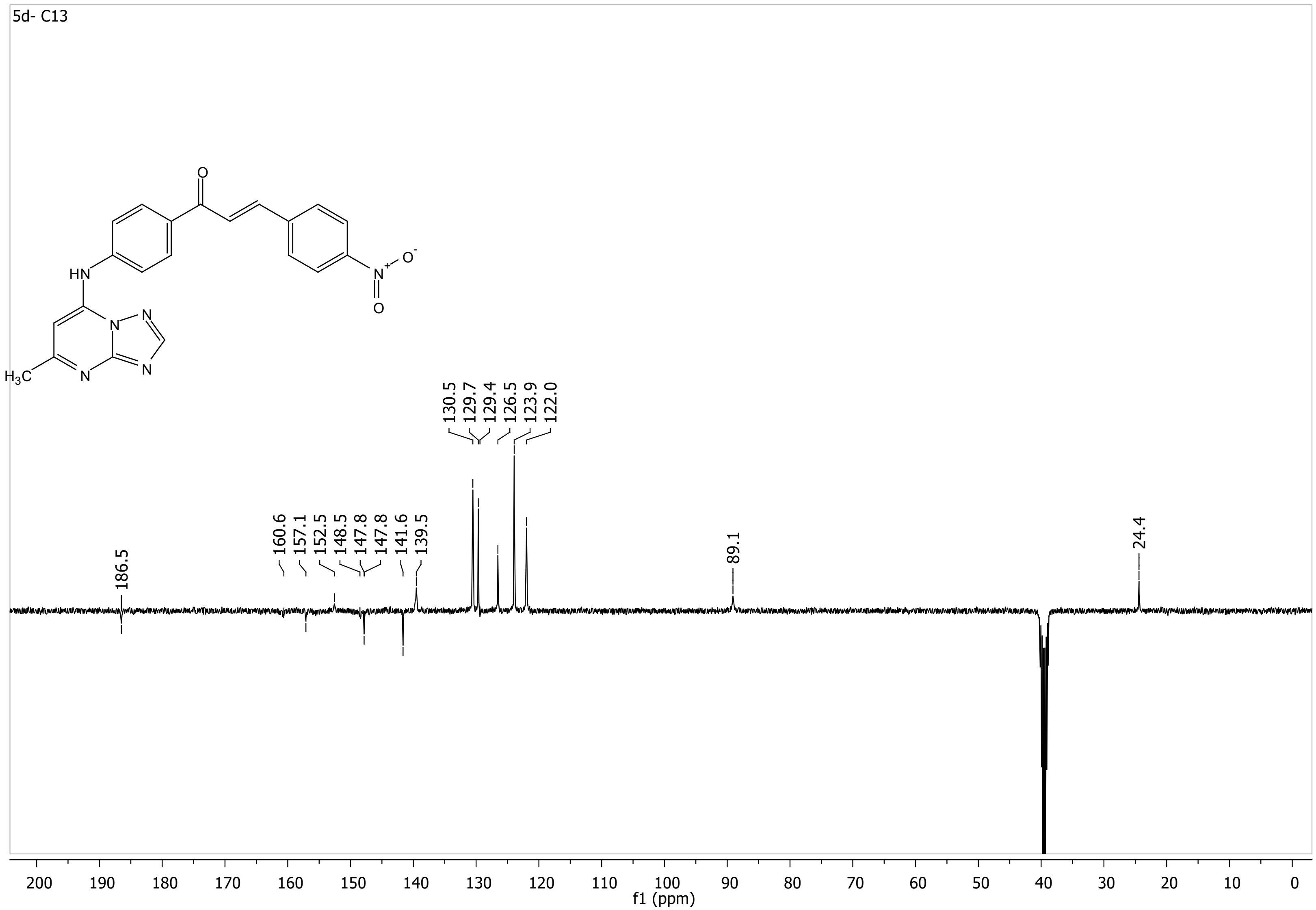
5d- C13
O
HN
N
N
N
N
H_3C
N^+
O^-
O
186.5
160.6
157.1
152.5
148.5
147.8
147.8
141.6
139.5
130.5
129.7
129.4
126.5
123.9
122.0
89.1
24.4
200 190 180 170 160 150 140 130 120 110 100 90 80 70 60 50 40 30 20 10 0
f1 (ppm)

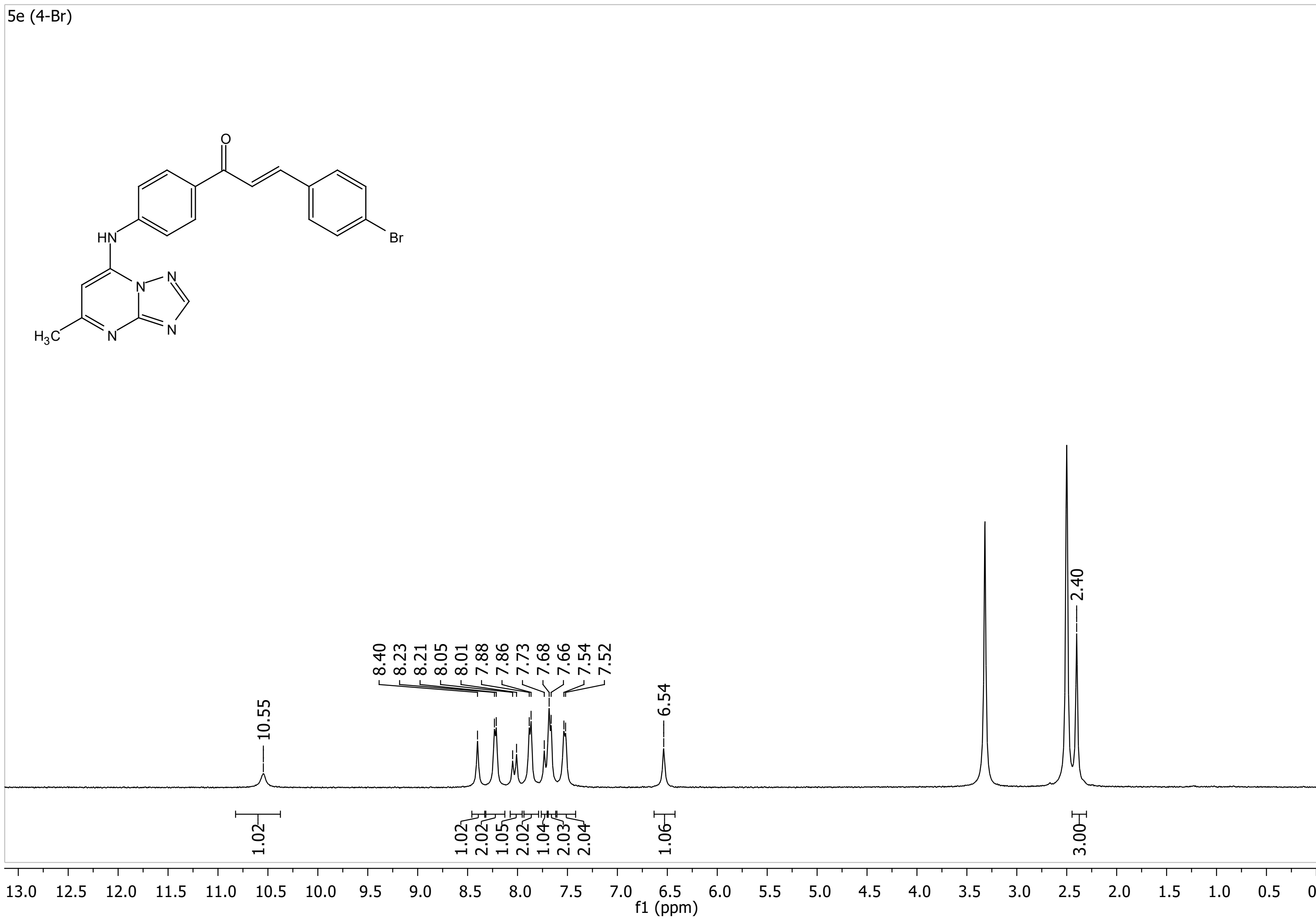
5e (4-Br)
O
HN
Br
N
N
N
N
H_3C
10.55
8.40
8.23
8.21
8.05
8.01
7.88
7.86
7.73
7.68
7.66
7.54
7.52
6.54
2.40
1.02
1.02
2.02
1.05
2.02
1.04
2.03
2.04
1.06
3.00
13.0 12.5 12.0 11.5 11.0 10.5 10.0 9.5 9.0 8.5 8.0 7.5 7.0 6.5 6.0 5.5 5.0 4.5 4.0 3.5 3.0 2.5 2.0 1.5 1.0 0.5 0.0
f1 (ppm)

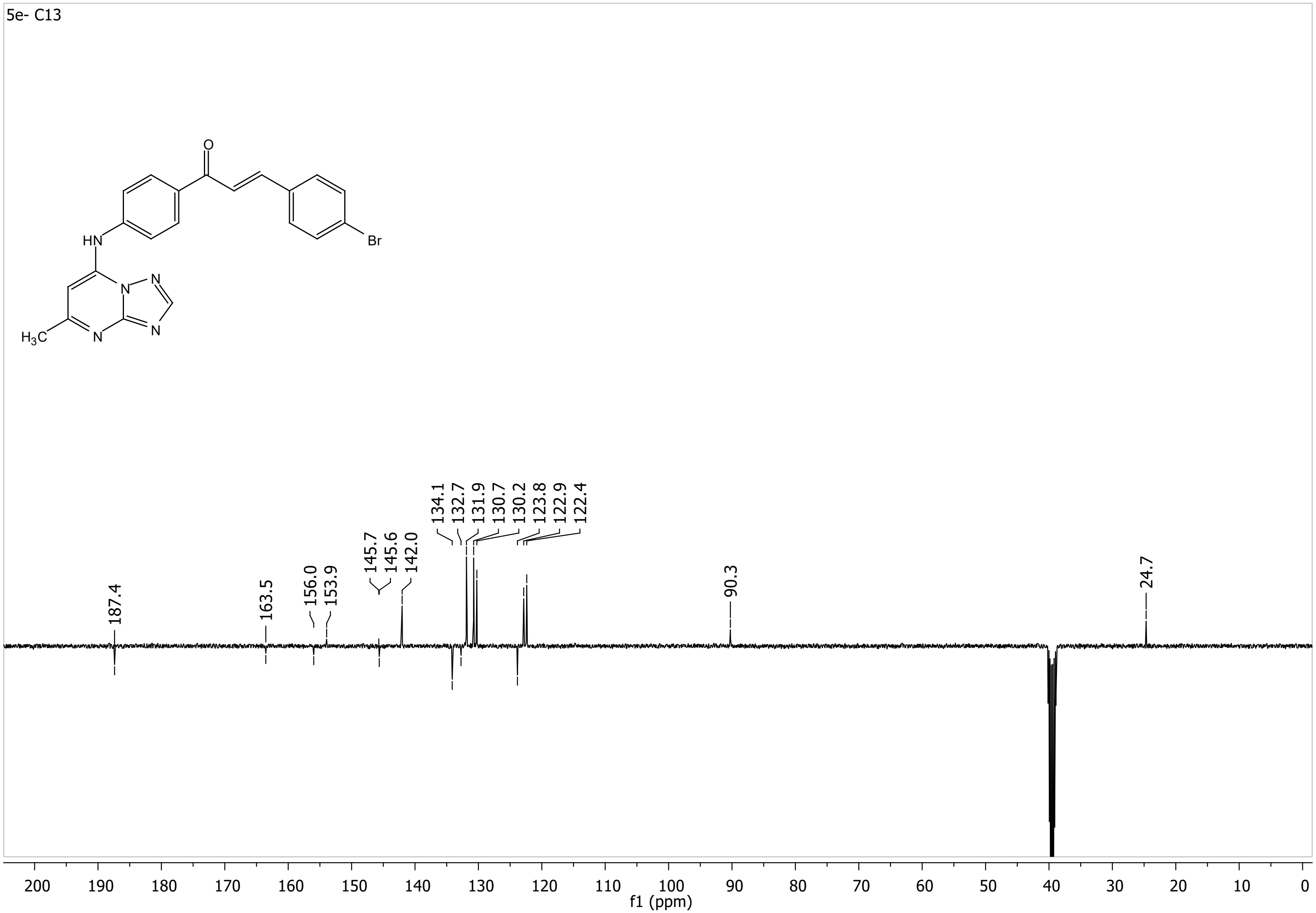
5e- C13
O
HN
Br
N
N
N
N
H3C
187.4
163.5
156.0
153.9
145.7
145.6
142.0
134.1
132.7
131.9
130.7
130.2
123.8
122.9
122.4
90.3
24.7
f1 (ppm)

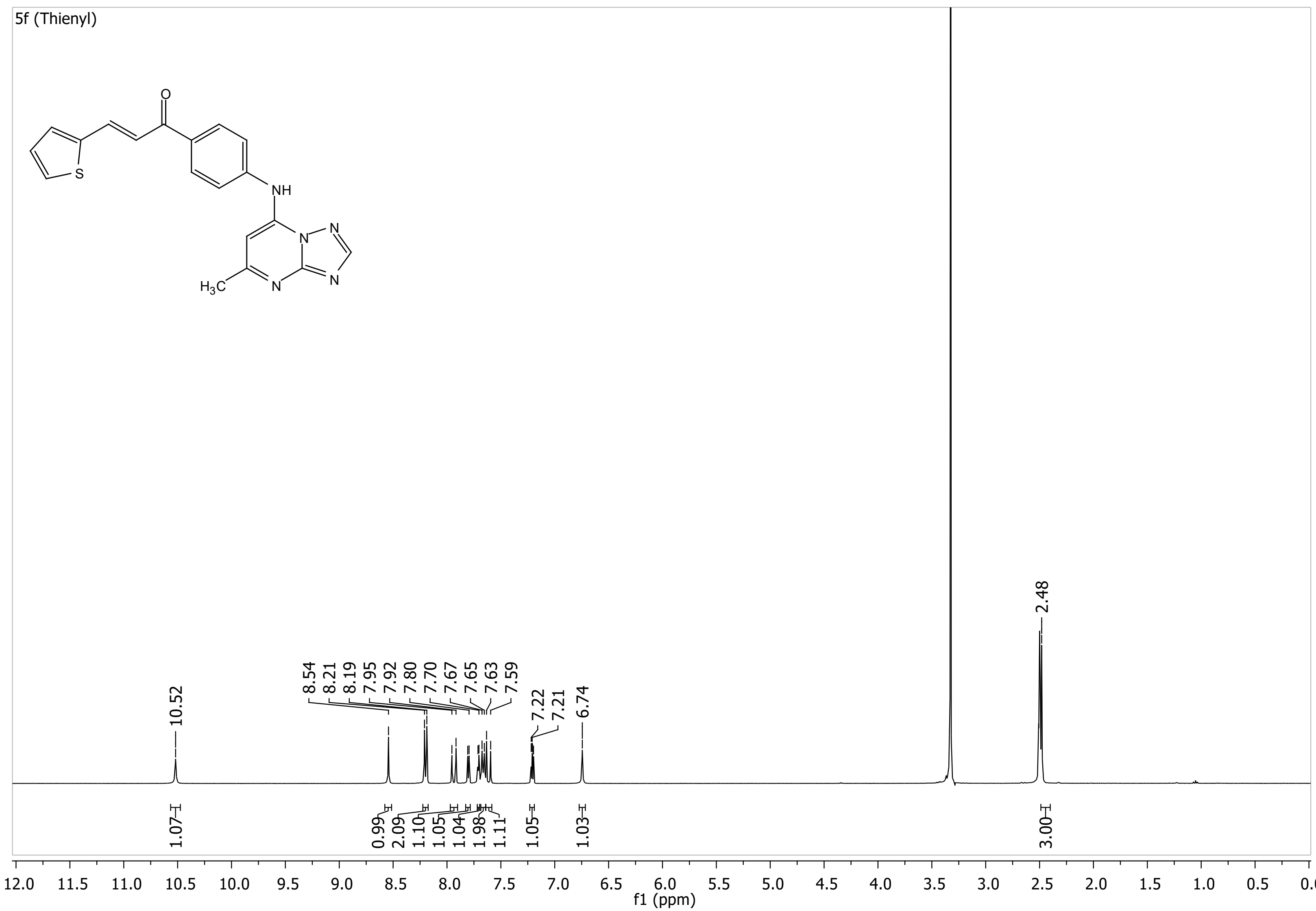
5f (Thienyl)
O
S
NH
N
N
N
N
H3C
10.52
8.54
8.21
8.19
7.95
7.92
7.80
7.70
7.67
7.65
7.63
7.59
7.22
7.21
6.74
2.48
1.07
0.99
2.09
1.10
1.05
1.04
1.98
1.11
1.05
1.03
3.00
f1 (ppm)

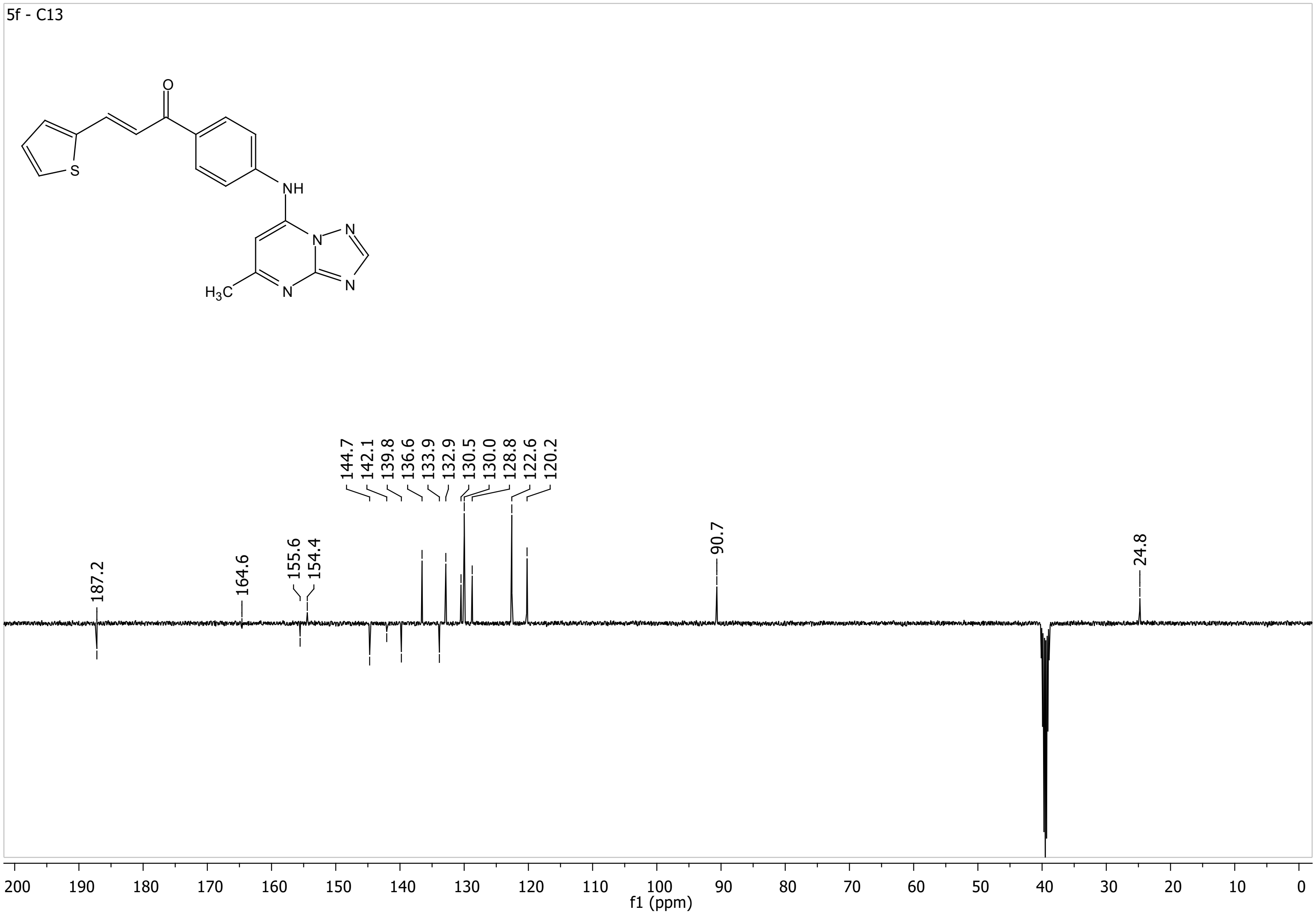
5f - C13
O
S
NH
N
N
N
N
H3C
187.2
164.6
155.6
154.4
144.7
142.1
139.8
136.6
133.9
132.9
130.5
130.0
128.8
122.6
120.2
90.7
24.8
200 190 180 170 160 150 140 130 120 110 100 90 80 70 60 50 40 30 20 10 0
f1 (ppm)

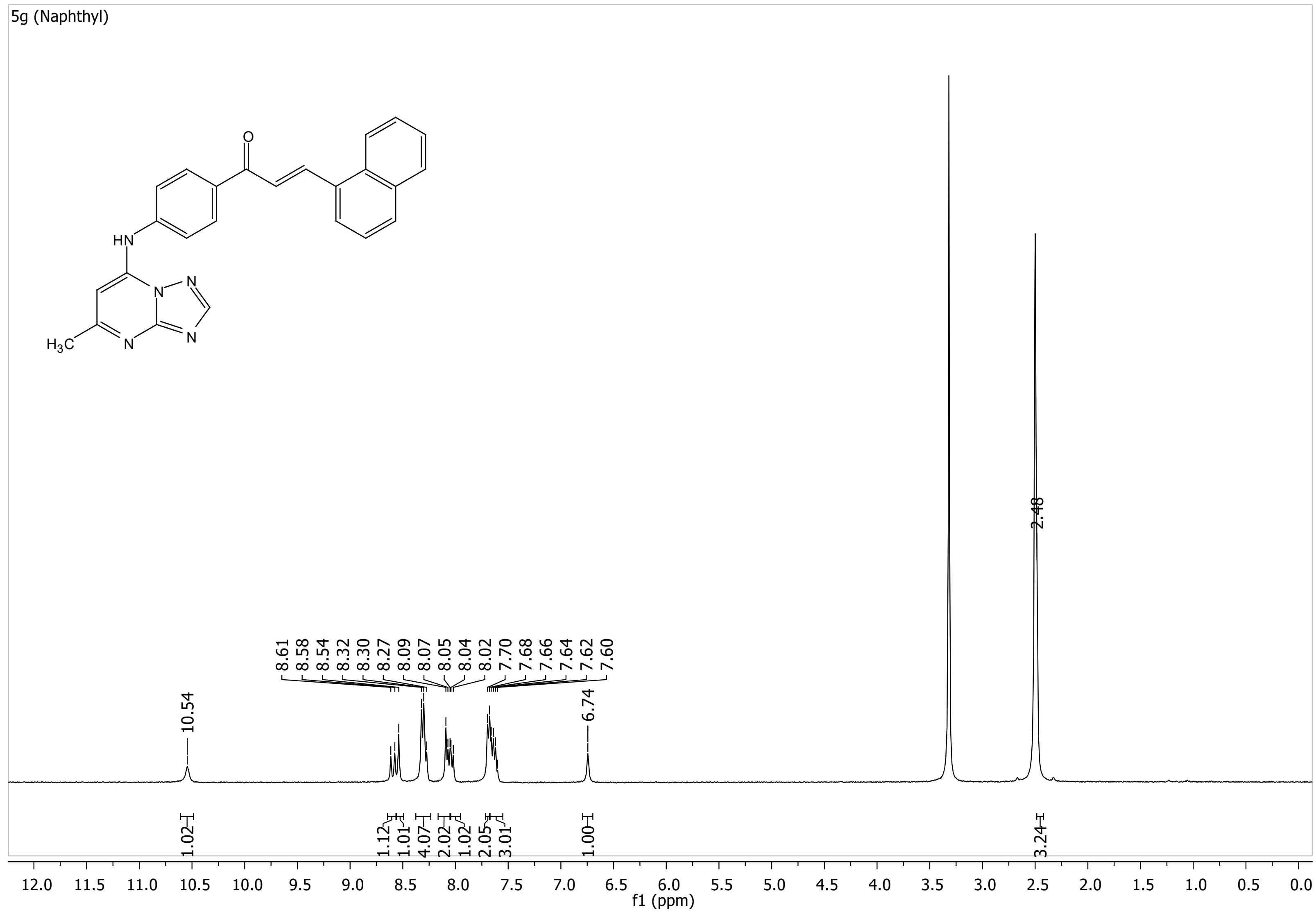
5g (Naphthyl)
O
HN
N
N
N
N
H3C
10.54
8.61
8.58
8.54
8.32
8.30
8.27
8.09
8.07
8.05
8.04
8.02
7.70
7.68
7.66
7.64
7.62
7.60
6.74
2.48
1.02
1.12
1.01
4.07
2.02
1.02
2.05
3.01
1.00
3.24
12.0 11.5 11.0 10.5 10.0 9.5 9.0 8.5 8.0 7.5 7.0 6.5 6.0 5.5 5.0 4.5 4.0 3.5 3.0 2.5 2.0 1.5 1.0 0.5 0.0
f1 (ppm)

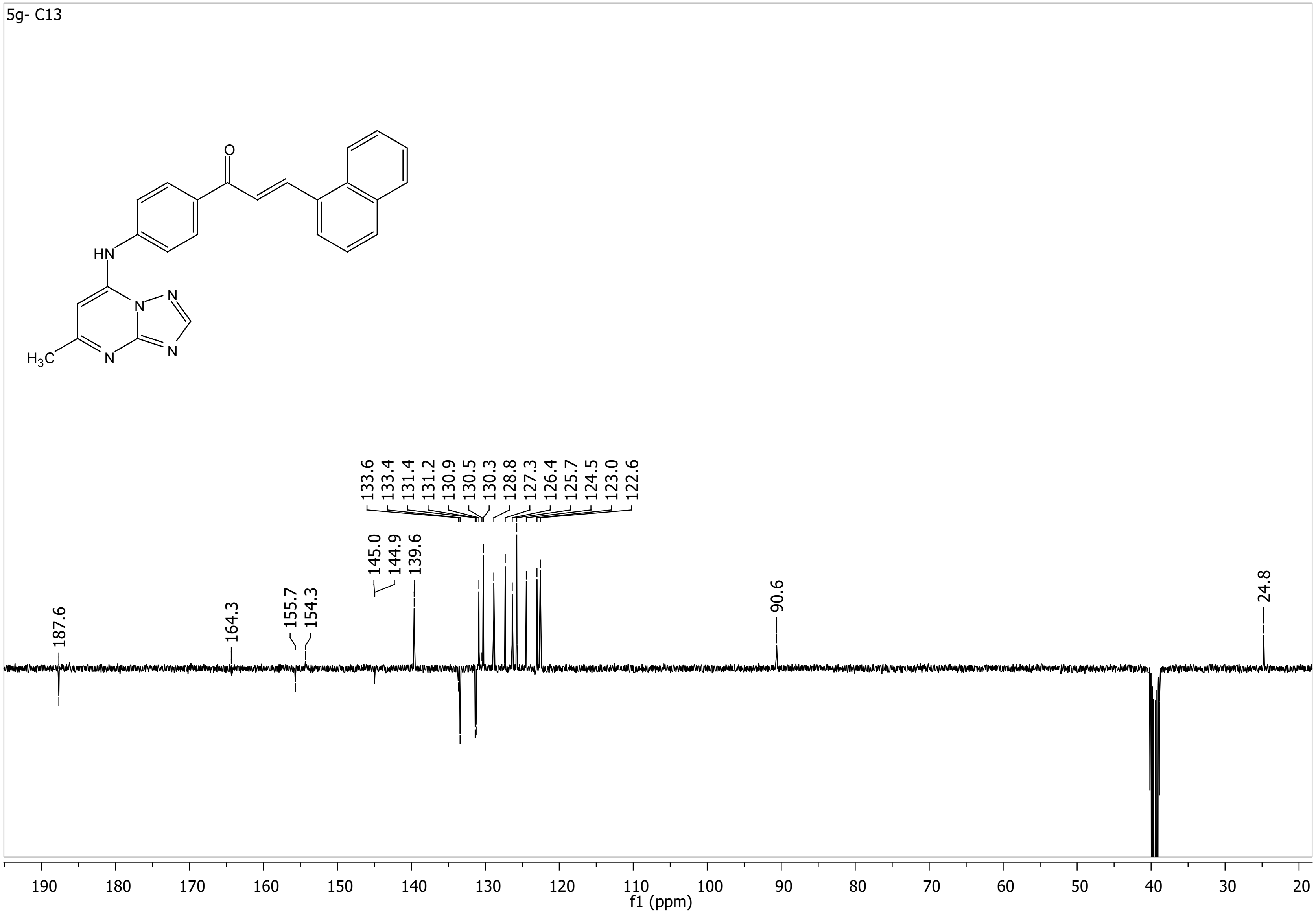
5g- C13
O
HN
N
N
N
N
H3C
187.6
164.3
155.7
154.3
145.0
144.9
139.6
133.6
133.4
131.4
131.2
130.9
130.5
130.3
128.8
127.3
126.4
125.7
124.5
123.0
122.6
90.6
24.8
190 180 170 160 150 140 130 120 110 100 90 80 70 60 50 40 30 20
f1 (ppm)

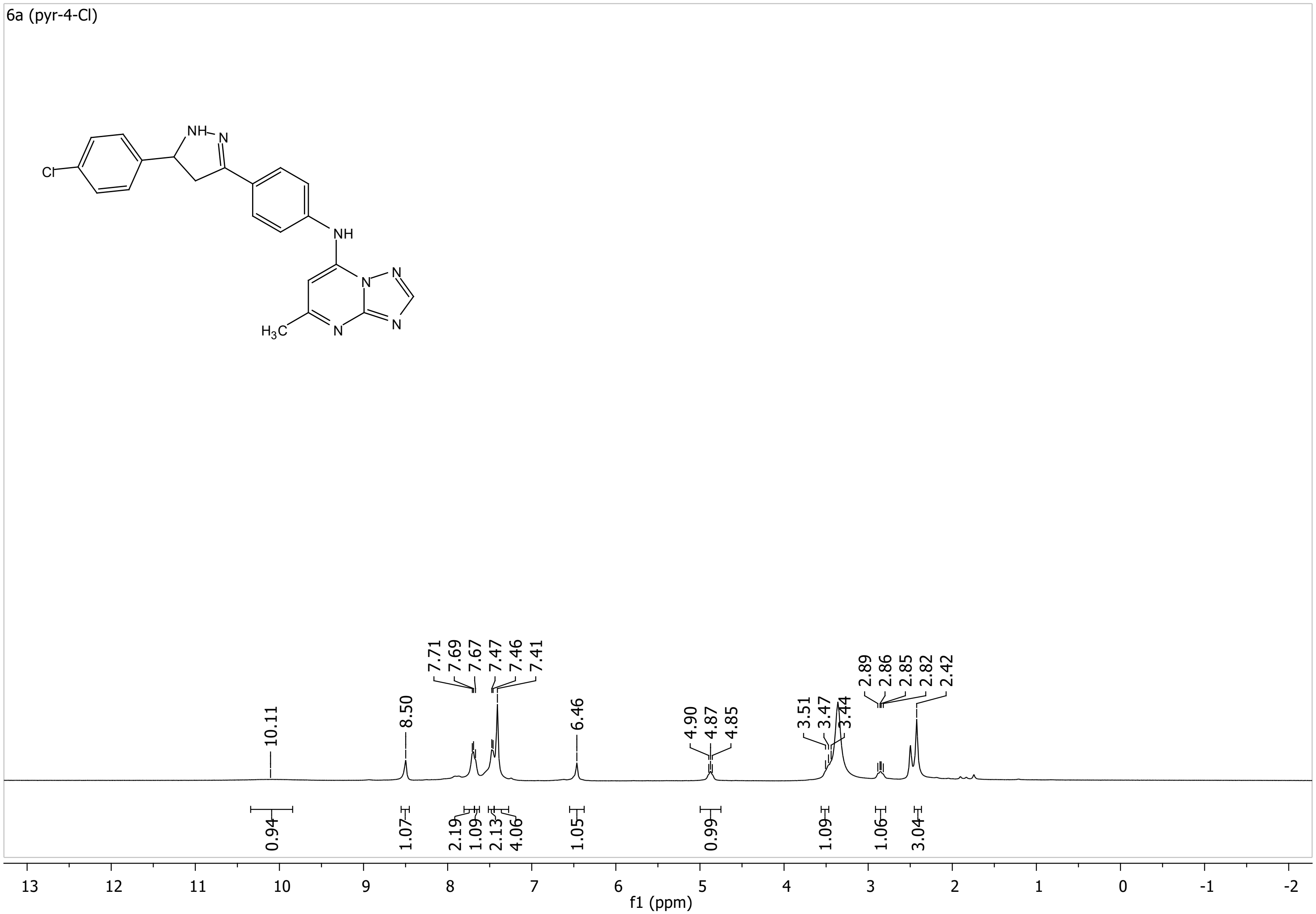
6a (pyr-4-Cl)
10.11
8.50
7.71
7.69
7.67
7.47
7.46
7.41
6.46
4.90
4.87
4.85
3.51
3.47
3.44
2.89
2.86
2.85
2.82
2.42
0.94
1.07
2.19
1.09
2.13
4.06
1.05
0.99
1.09
1.06
3.04
f1 (ppm)

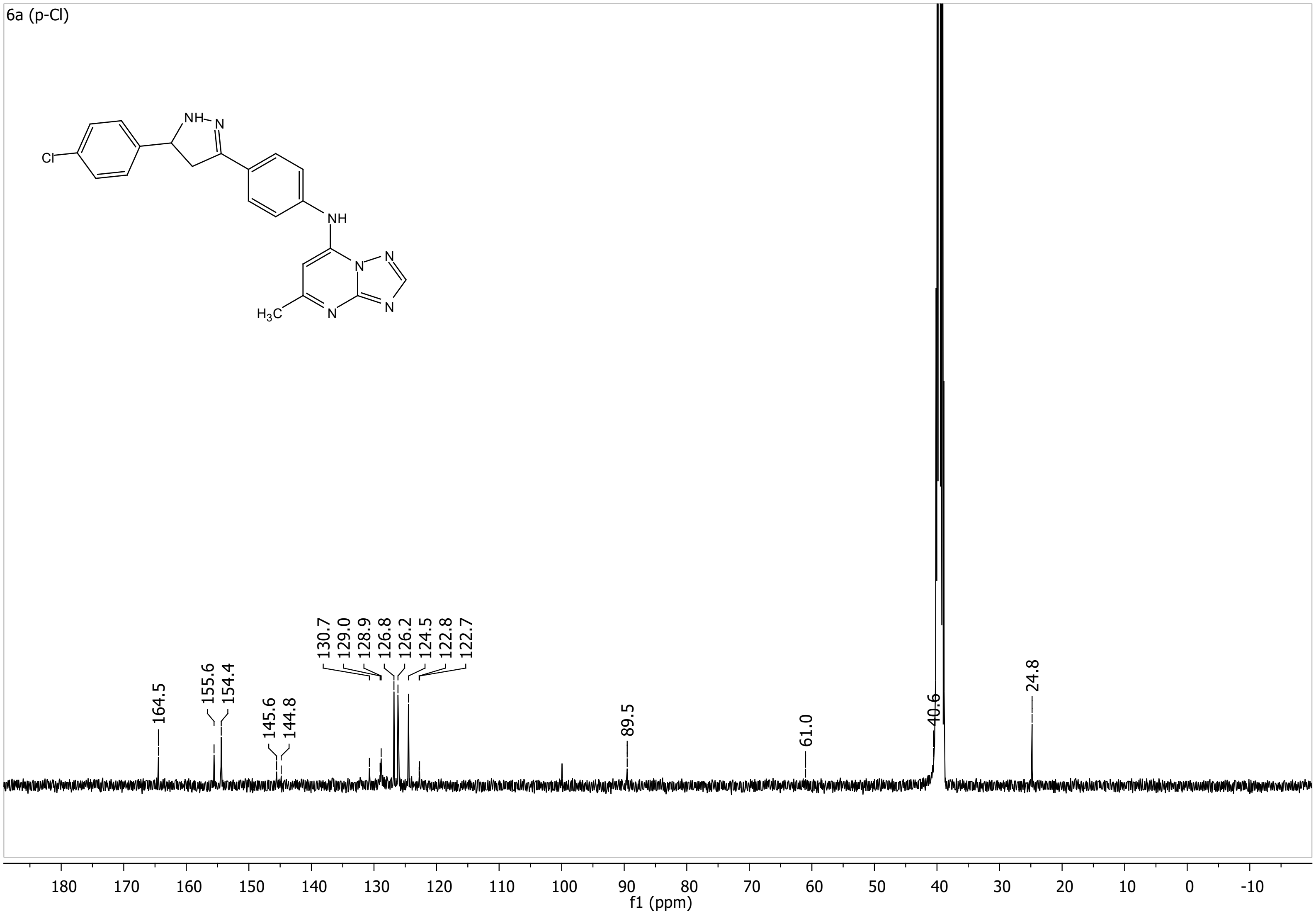
6a (p-Cl)
Cl
NH
N
NH
N
N
N
N
H3C
164.5
155.6
154.4
145.6
144.8
130.7
129.0
128.9
126.8
126.2
124.5
122.8
122.7
89.5
61.0
40.6
24.8
180 170 160 150 140 130 120 110 100 90 80 70 60 50 40 30 20 10 0 -10
f1 (ppm)

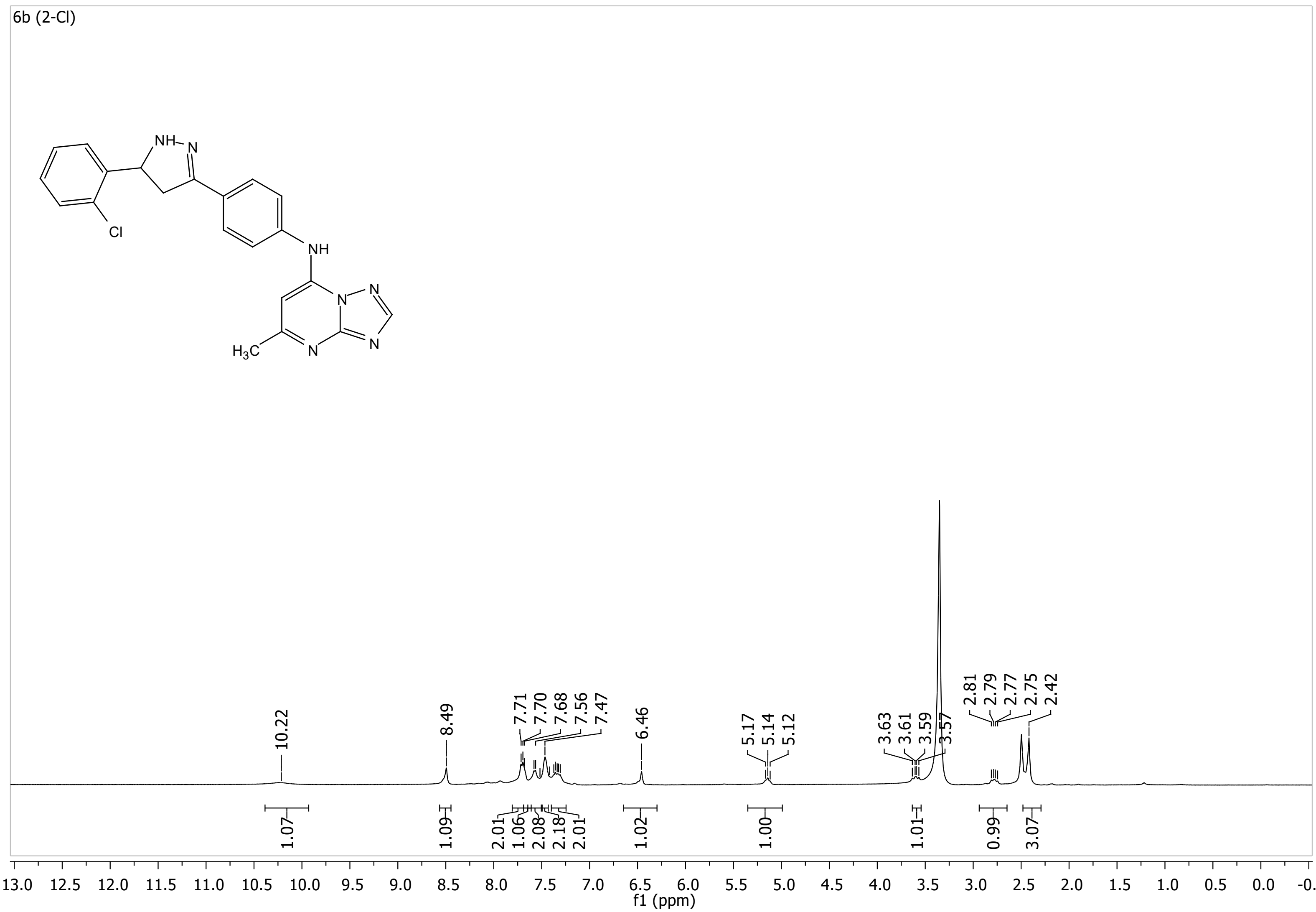

6b (2-Cl)
NH
N
Cl
NH
N
N
N
N
H3C
10.22
8.49
7.71
7.70
7.68
7.56
7.47
6.46
5.17
5.14
5.12
3.63
3.61
3.59
3.57
2.81
2.79
2.77
2.75
2.42
1.07
1.09
2.01
1.06
2.08
2.18
2.01
1.02
1.00
1.01
0.99
3.07
13.0 12.5 12.0 11.5 11.0 10.5 10.0 9.5 9.0 8.5 8.0 7.5 7.0 6.5 6.0 5.5 5.0 4.5 4.0 3.5 3.0 2.5 2.0 1.5 1.0 0.5 0.0 -0.5
f1 (ppm)

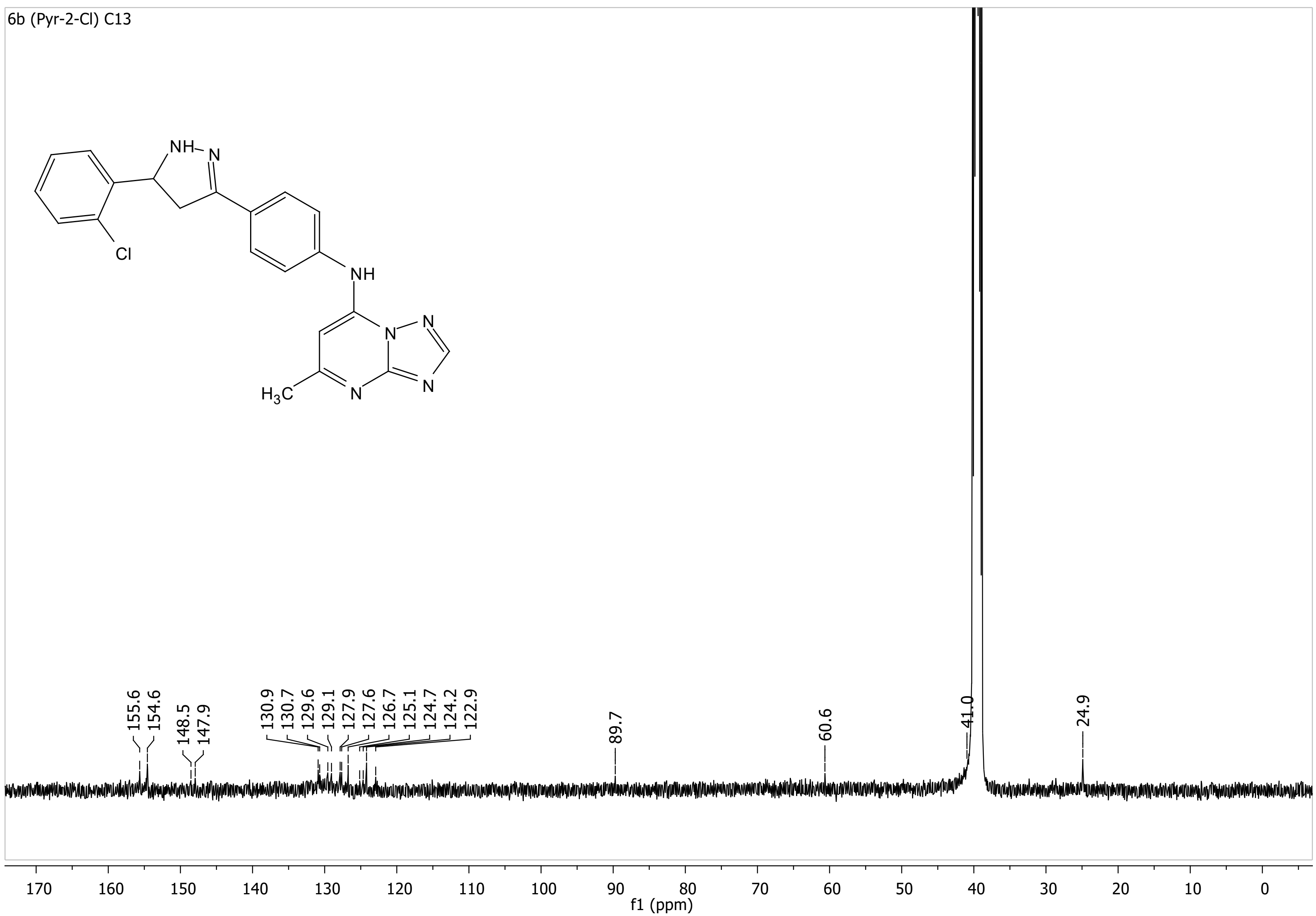
6b (Pyr-2-Cl) C13
NH
N
Cl
NH
N
N
N
N
H_3C
155.6
154.6
148.5
147.9
130.9
130.7
129.6
129.1
127.9
127.6
126.7
125.1
124.7
124.2
122.9
89.7
60.6
41.0
24.9
170 160 150 140 130 120 110 100 90 80 70 60 50 40 30 20 10 0
f1 (ppm)

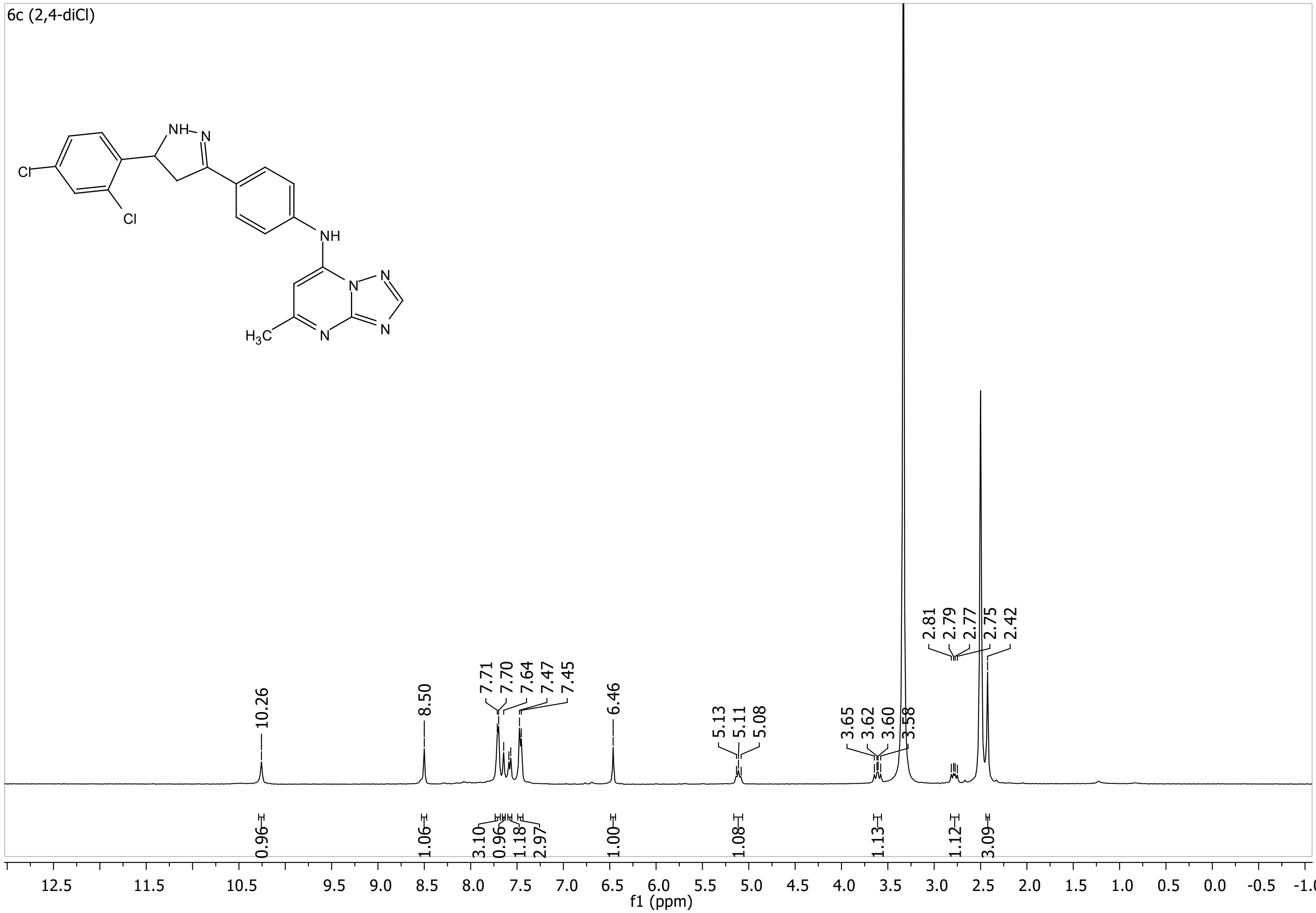
6c (2,4-diCl)
Cl
Cl
NH
N
NH
N
N
N
N
H3C
10.26
8.50
7.71
7.70
7.64
7.47
7.45
6.46
5.13
5.11
5.08
3.65
3.62
3.60
3.58
2.81
2.79
2.77
2.75
2.42
0.96
1.06
3.10
0.96
1.18
2.97
1.00
1.08
1.13
1.12
3.09
f1 (ppm)

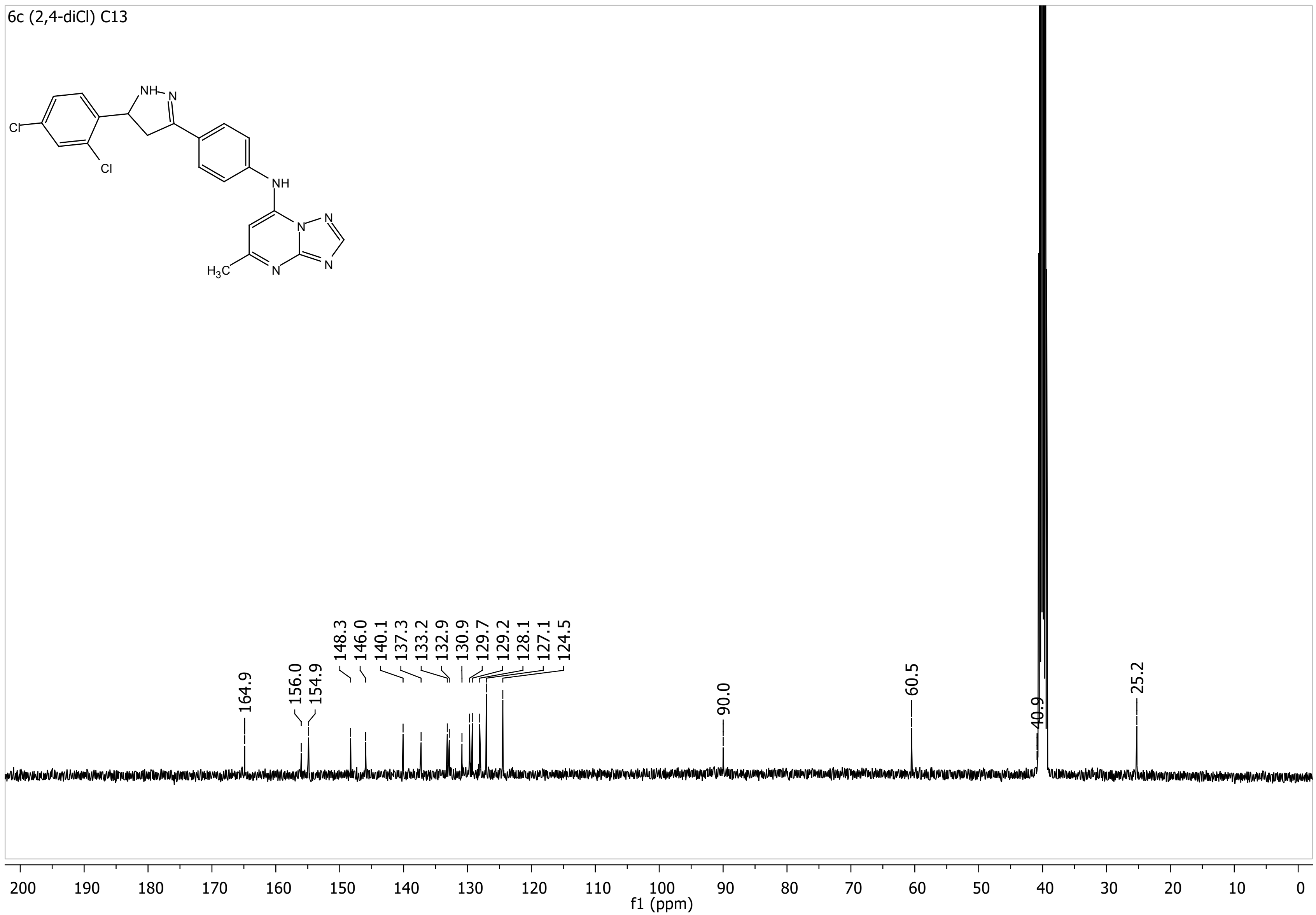
6c (2,4-diCl) C13
NH
N
Cl
Cl
NH
N
N
N
N
H3C
164.9
156.0
154.9
148.3
146.0
140.1
137.3
133.2
132.9
130.9
129.7
129.2
128.1
127.1
124.5
90.0
60.5
40.9
25.2
200 190 180 170 160 150 140 130 120 110 100 90 80 70 60 50 40 30 20 10 0
f1 (ppm)

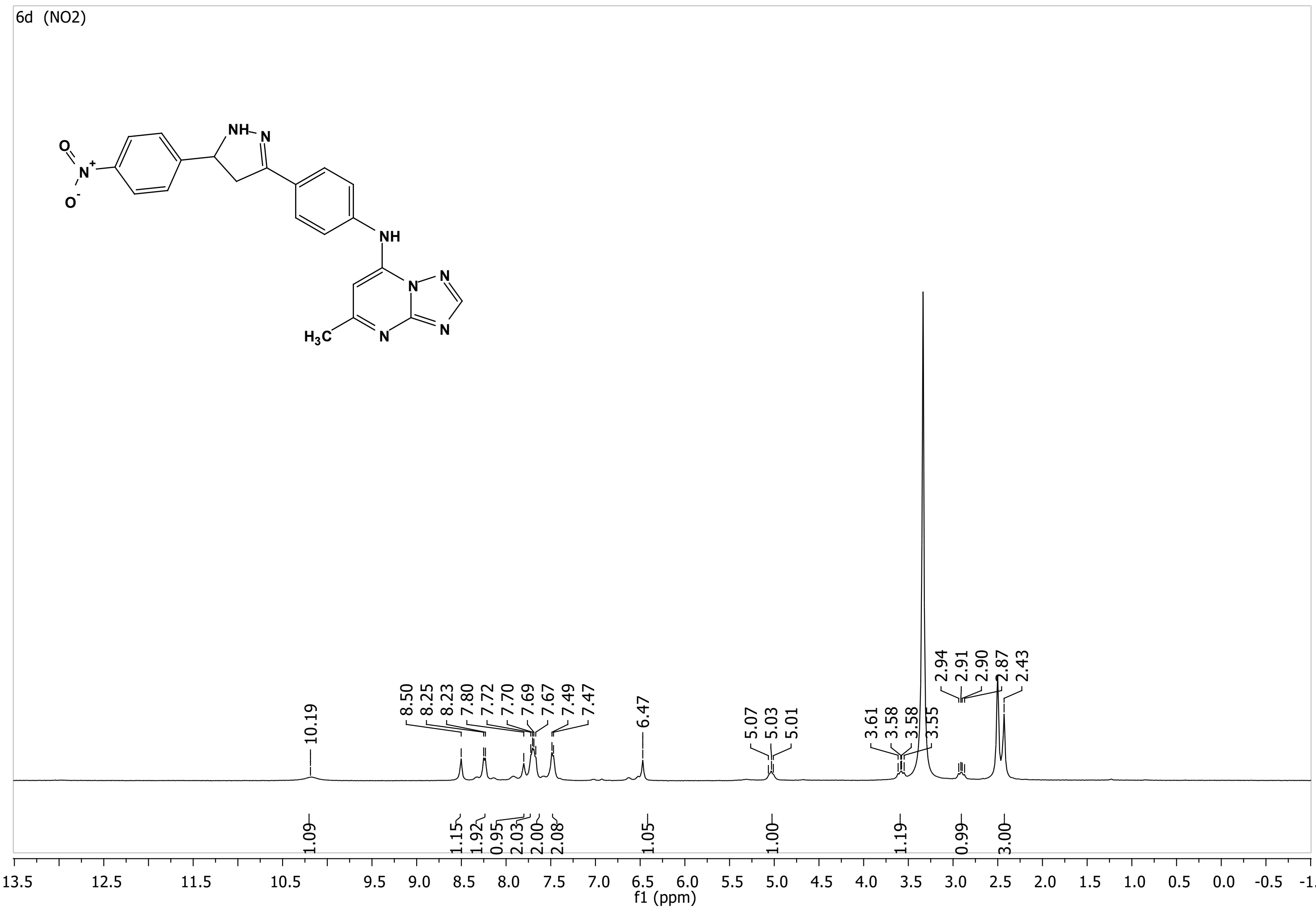
6d (NO2)
NH
N
O
N+
O-
NH
N
N
N
N
H3C
10.19
8.50
8.25
8.23
7.80
7.72
7.70
7.69
7.67
7.49
7.47
6.47
5.07
5.03
5.01
3.61
3.58
3.58
3.55
2.94
2.91
2.90
2.87
2.43
1.09
1.15
1.92
0.95
2.03
2.00
2.08
1.05
1.00
1.19
0.99
3.00
f1 (ppm)

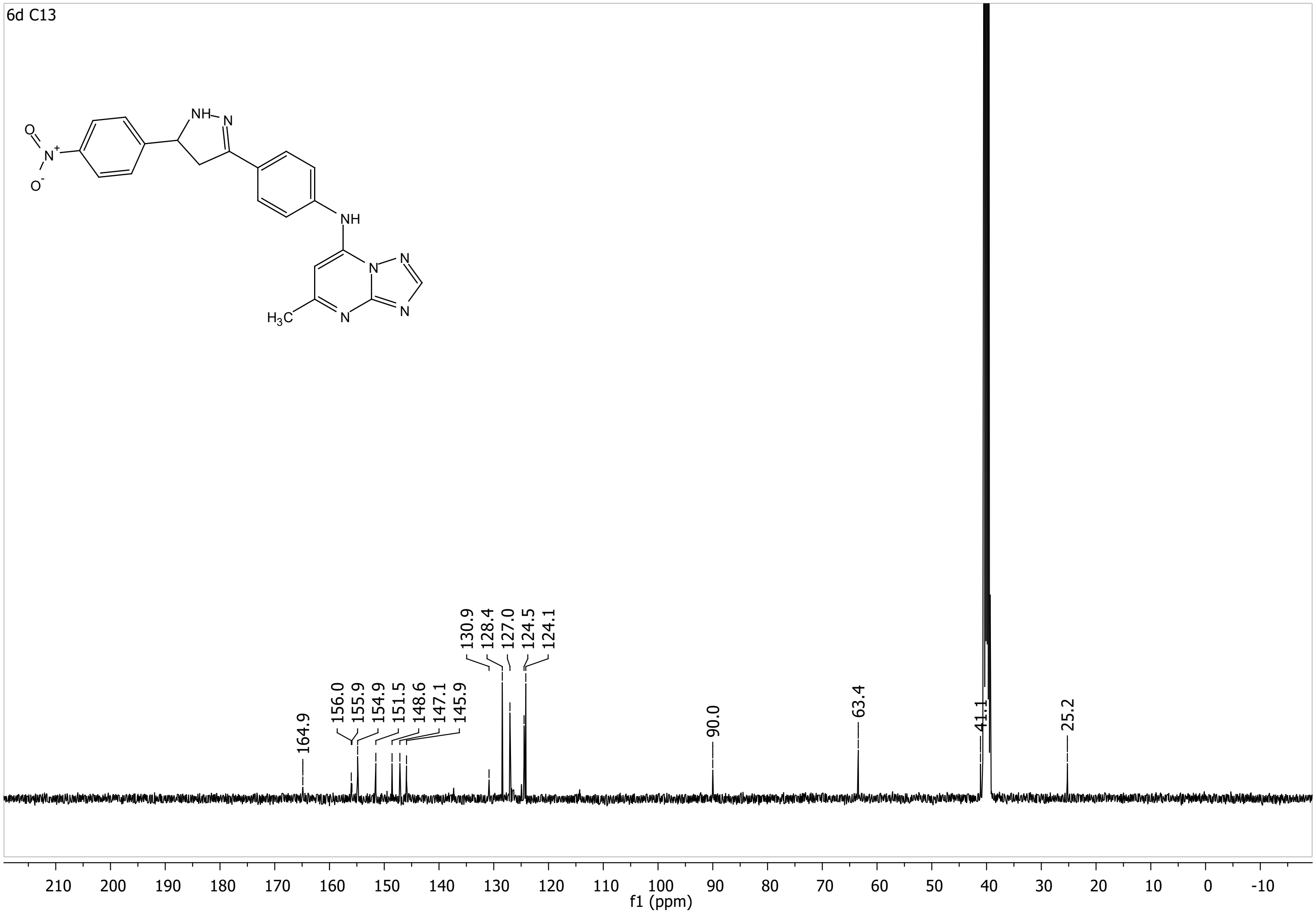
6d C13
164.9
156.0
155.9
154.9
151.5
148.6
147.1
145.9
130.9
128.4
127.0
124.5
124.1
90.0
63.4
41.1
25.2
210 200 190 180 170 160 150 140 130 120 110 100 90 80 70 60 50 40 30 20 10 0 -10
f1 (ppm)

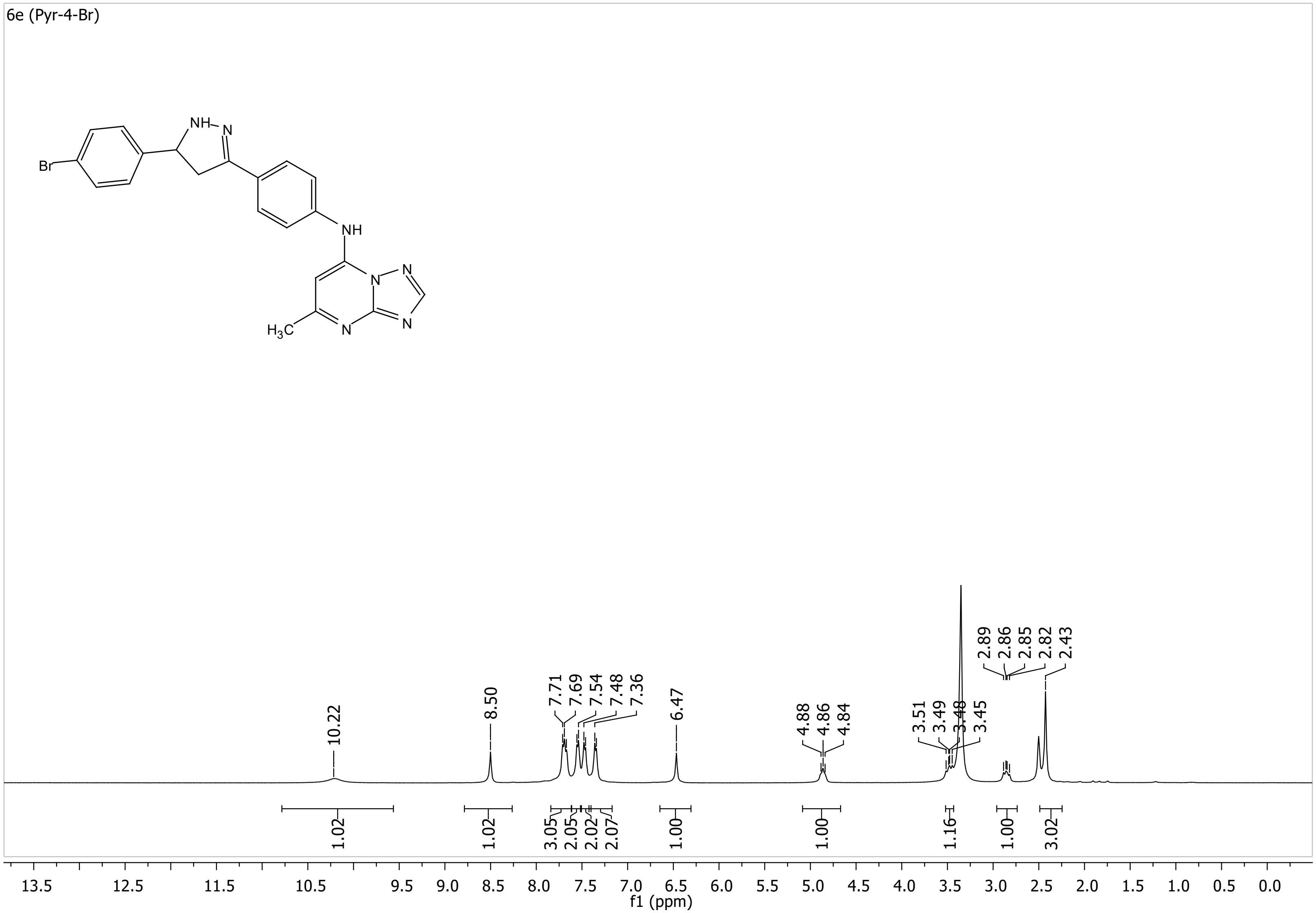
6e (Pyr-4-Br)
Br
NH
N
NH
N
N
N
N
H3C
10.22
8.50
7.71
7.69
7.54
7.48
7.36
6.47
4.88
4.86
4.84
3.51
3.49
3.48
3.45
2.89
2.86
2.85
2.82
2.43
1.02
1.02
3.05
2.05
2.02
2.07
1.00
1.00
1.16
1.00
3.02
13.5 12.5 11.5 10.5 9.5 9.0 8.5 8.0 7.5 7.0 6.5 6.0 5.5 5.0 4.5 4.0 3.5 3.0 2.5 2.0 1.5 1.0 0.5 0.0
f1 (ppm)

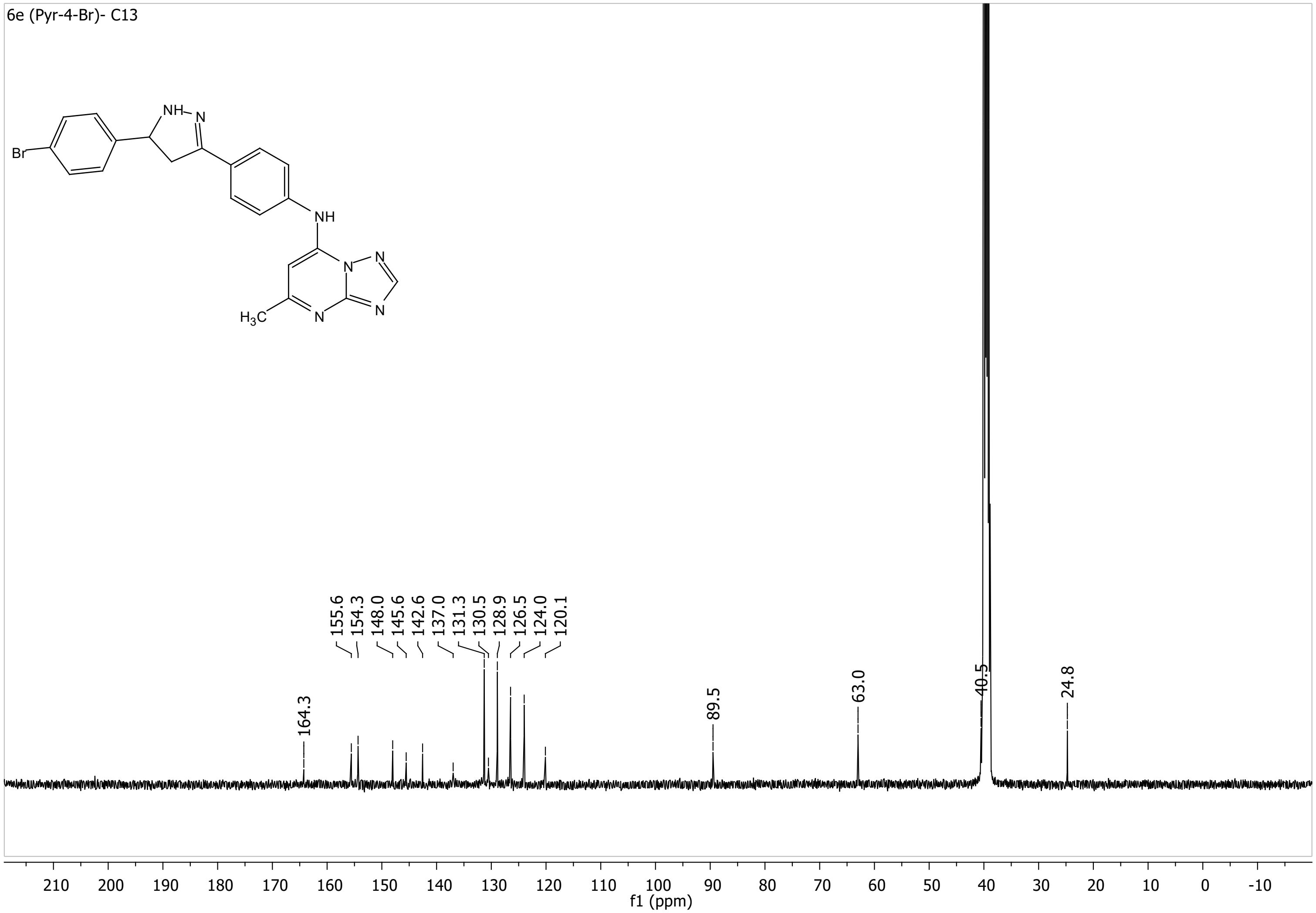
6e (Pyr-4-Br)- C13
Br
NH
N
NH
N
N
N
N
H3C
164.3
155.6
154.3
148.0
145.6
142.6
137.0
131.3
130.5
128.9
126.5
124.0
120.1
89.5
63.0
40.5
24.8
210 200 190 180 170 160 150 140 130 120 110 100 90 80 70 60 50 40 30 20 10 0 -10
f1 (ppm)

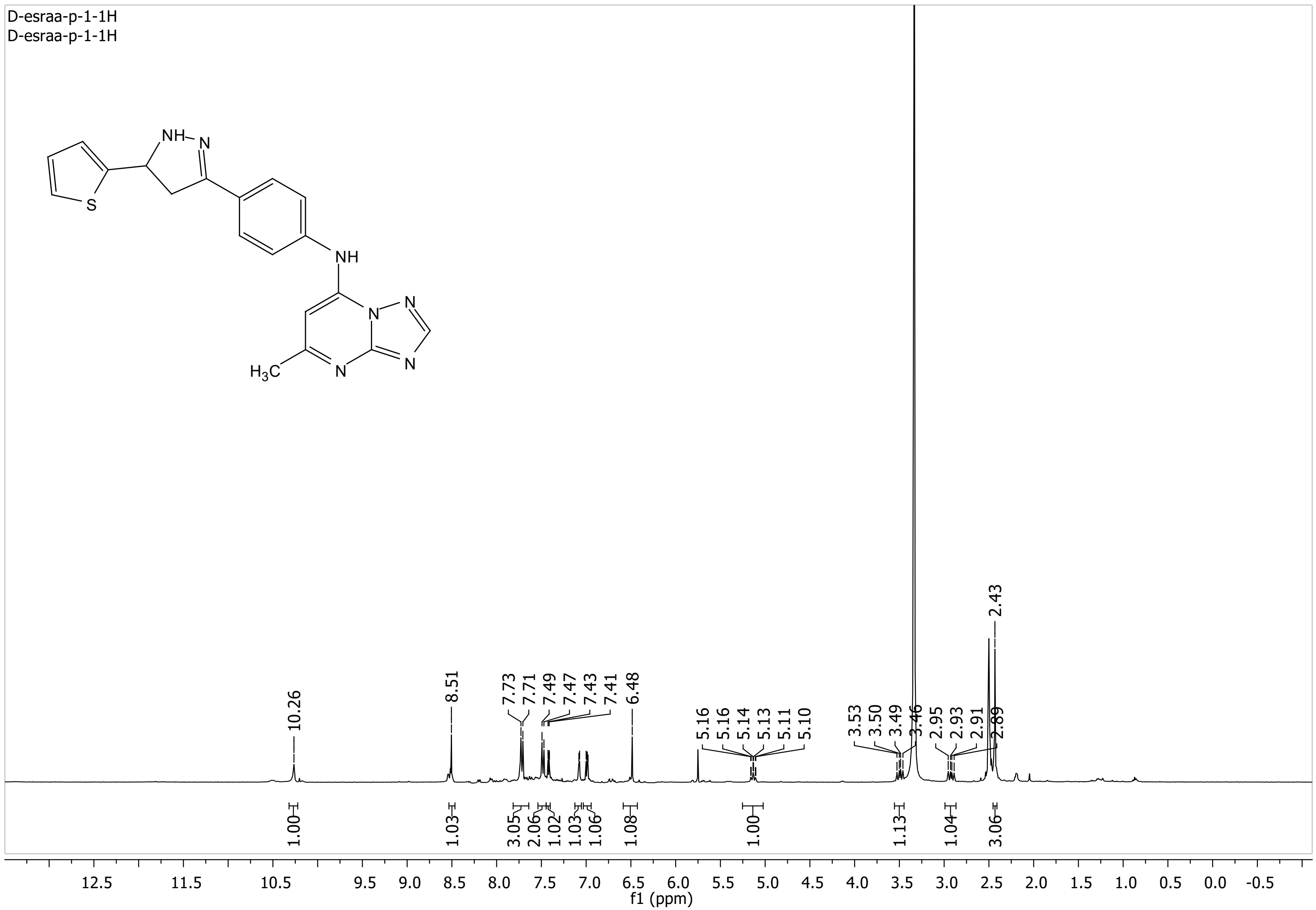
D-esraa-p-1-1H
D-esraa-p-1-1H
NH
N
S
NH
N
N
N
N
H3C
10.26
8.51
7.73
7.71
7.49
7.47
7.43
7.41
6.48
5.16
5.16
5.14
5.13
5.11
5.10
3.53
3.50
3.49
3.46
2.95
2.93
2.91
2.89
2.43
1.00
1.03
3.05
2.06
1.02
1.03
1.06
1.08
1.00
1.13
1.04
3.06
12.5
11.5
10.5
9.5
9.0
8.5
8.0
7.5
7.0
6.5
6.0
5.5
5.0
4.5
4.0
3.5
3.0
2.5
2.0
1.5
1.0
0.5
0.0
-0.5
f1 (ppm)

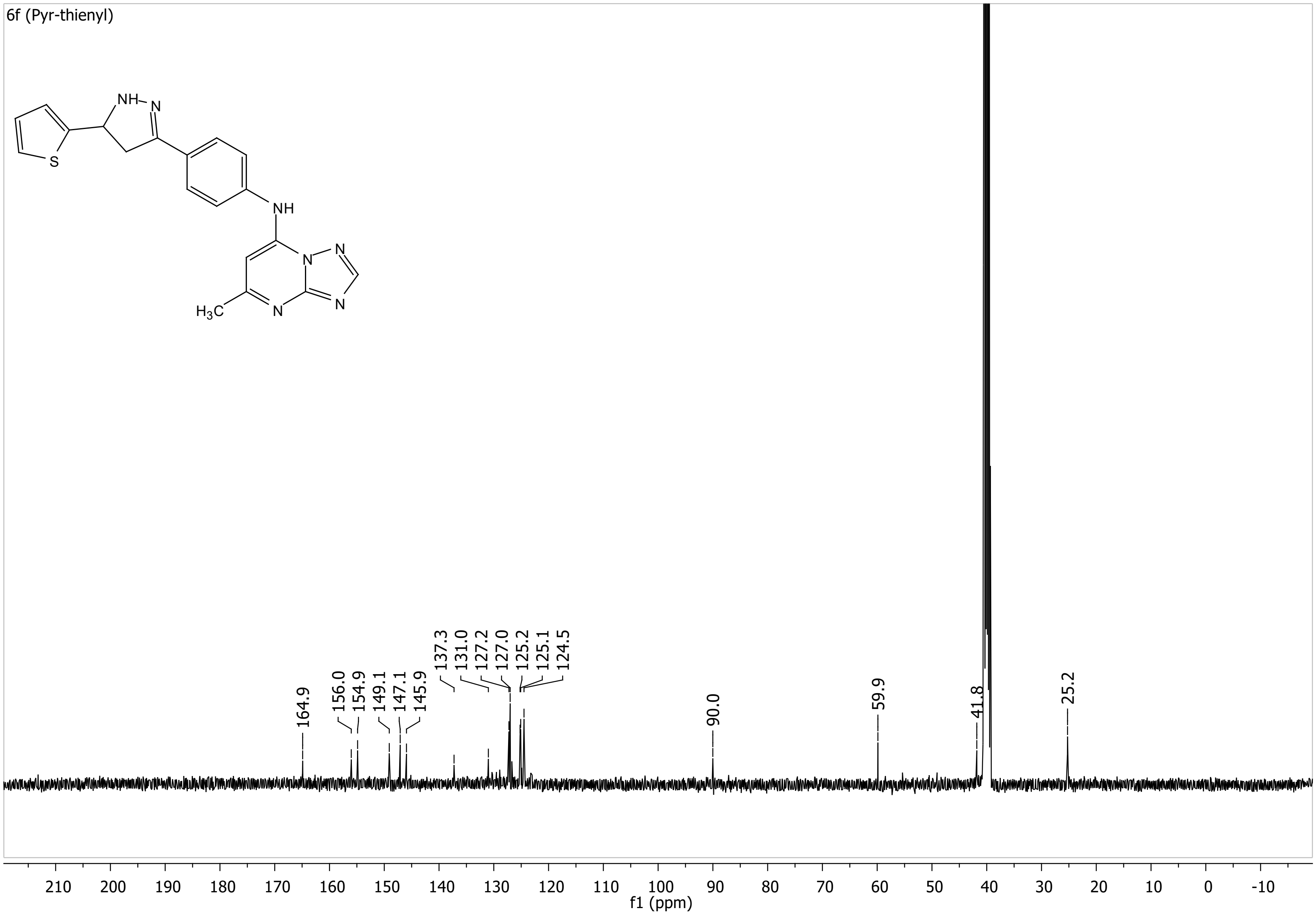
6f (Pyr-thienyl)
NH
N
S
NH
N
N
N
H3C
N
164.9
156.0
154.9
149.1
147.1
145.9
137.3
131.0
127.2
127.0
125.2
125.1
124.5
90.0
59.9
41.8
25.2
210 200 190 180 170 160 150 140 130 120 110 100 90 80 70 60 50 40 30 20 10 0 -10
f1 (ppm)

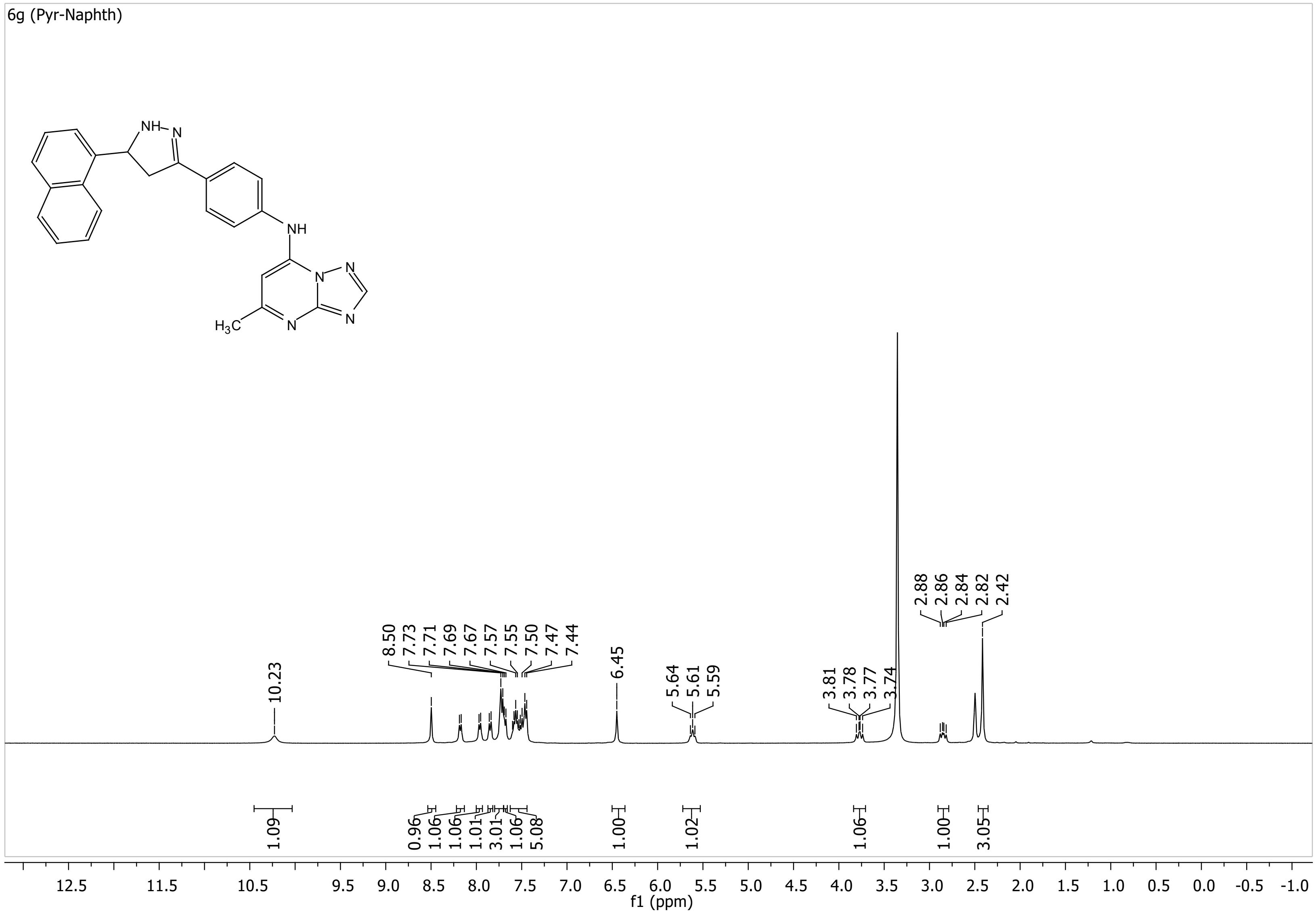
6g (Pyr-Naphth)
10.23
8.50
7.73
7.71
7.69
7.67
7.57
7.55
7.50
7.47
7.44
6.45
5.64
5.61
5.59
3.81
3.78
3.77
3.74
2.88
2.86
2.84
2.82
2.42
1.09
0.96
1.06
1.06
1.01
3.01
1.06
5.08
1.00
1.02
1.06
1.00
3.05
f1 (ppm)

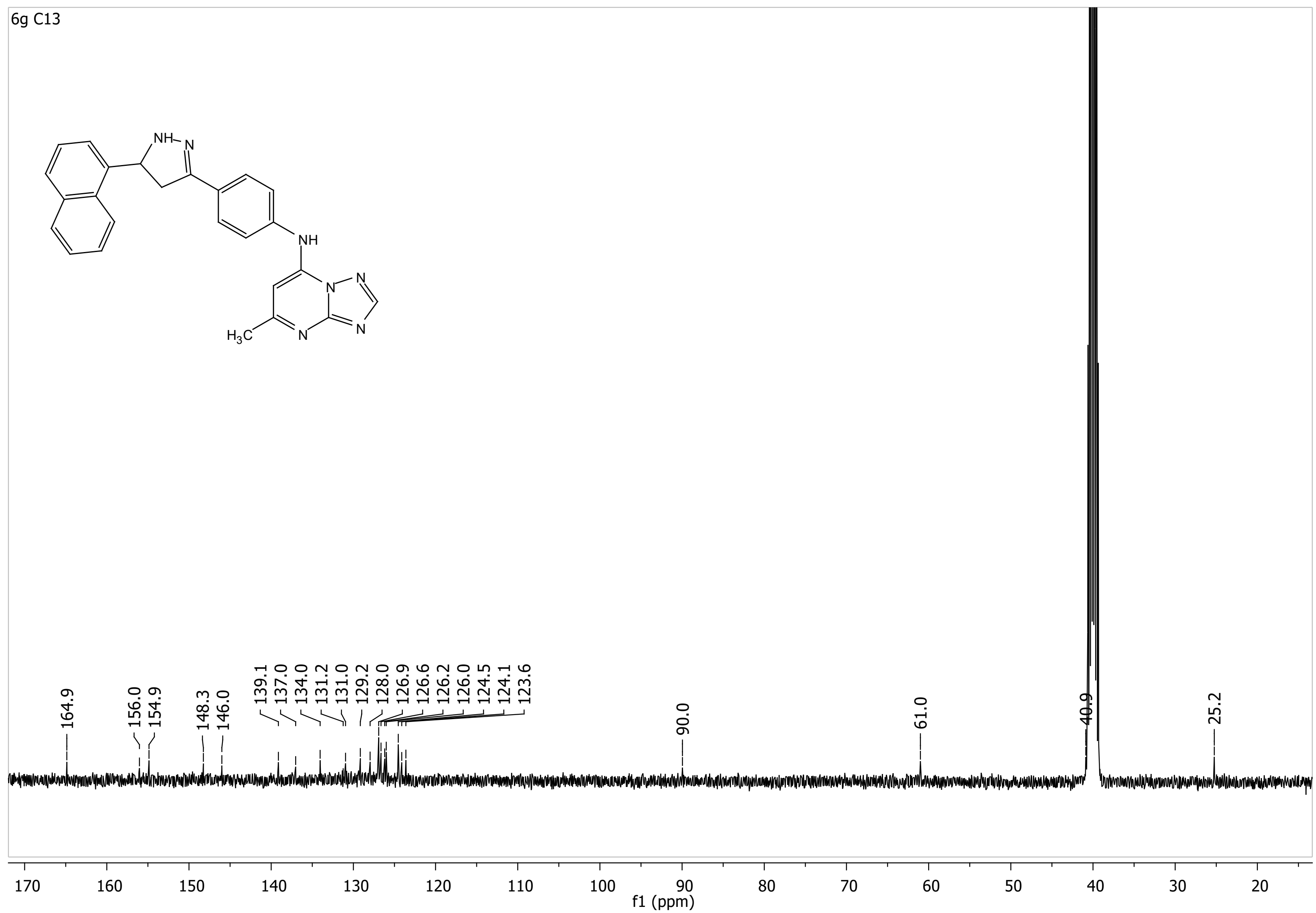

6g C13
NH
N
NH
N
N
N
N
H3C
164.9
156.0
154.9
148.3
146.0
139.1
137.0
134.0
131.2
131.0
129.2
128.0
126.9
126.6
126.2
126.0
124.5
124.1
123.6
90.0
61.0
40.9
25.2
170 160 150 140 130 120 110 100 90 80 70 60 50 40 30 20
f1 (ppm)
